# Supplementary material for: MicroRNA‐574 regulates FAM210A expression and influences pathological cardiac remodeling
Source: EMBO Mol Med. 2020 Dec 28;13(2):e12710. doi: 10.15252/emmm.202012710 (PMC7863409; doi:10.15252/emmm.202012710)
Supplement: Supplementary file 1 — Appendix [file EMMM-13-e12710-s001.pdf]

MicroRNA-574 Regulates FAM210A Expression and Influences Pathological Cardiac Remodeling

Jiangbin Wu[1,‡], Kadiam C Venkata Subbaiah[1,‡], Feng Jiang[1,2], Omar Hadaya[1,2], Amy Mohan[1],

Tingting Yang[3], Kevin Welle[4], Sina Ghaemmaghami[4], Wai Hong Wilson Tang[5],

Eric Small[1], Chen Yan[1], Peng Yao[1,2,6,7,*]

[1] Aab Cardiovascular Research Institute, Department of Medicine, University of Rochester School of Medicine & Dentistry, Rochester, New York 14642

[2] Department of Biochemistry & Biophysics, University of Rochester School of Medicine & Dentistry, Rochester, New York 14642

[3] Department of Ophthalmology, Columbia University, New York, NY 10032 USA

[4] Mass Spectrometry Resource Lab, University of Rochester School of Medicine & Dentistry, Rochester, New York 14642

[5] Department of Cardiovascular Medicine, Cleveland Clinic, Cleveland, Ohio 44195

[6] The Center for RNA Biology, University of Rochester School of Medicine & Dentistry, Rochester, New York 14642

[7] The Center for Biomedical Informatics, University of Rochester School of Medicine & Dentistry, Rochester, New York 14642

* To whom correspondence should be addressed. Tel: +1 585 276 7708; E-mail: peng_yao@urmc.rochester.edu; Address: 601 Elmwood Avenue, Box CVRI, Rochester, NY 14642

Appendix

- **Appendix Materials and Methods**
- **Appendix Figure S1.** Generation of miR-574 global knockout mice and phenotypic characterization.
- **Appendix Figure S2.** Downregulated genes in miR-574 KO versus WT mouse heart.
- **Appendix Figure S3.** Organ-specific expression and cellular localization of FAM210A.
- **Appendix Figure S4.** Quantitative analysis of Western blot data from Figure 7F, G.
- **Appendix Figure S5.** miR-574-5p/3p injection reduces the cardiac pathological remodelling in ISO-treated miR-574 null mice.
- **Appendix Table S1.** 34 upregulated and 49 downregulated genes in miR-574 KO vs. WT hearts
- **Appendix Table S2.** FAM210A interactome capture by IP-mass spectrometry
- **Appendix References**

Appendix Materials and Methods

Plasmids, antibodies, miRNAs, siRNAs, and qPCR primers

Full-length coding sequence (CDS) of human FAM210A was cloned into pcDNA3.1(+) with 3x Flag in the C-terminus of the genes. Full-length CDS of mouse Fam210a was cloned into pEGFP-N1 with EGFP in the C-terminus of the gene. The primer sequences for cloning FAM210A gene are as follows:

FAM210A-3xFlag-F (BamHI): 5' CGGGATCCCAAAATGCAATGGAATGTACCACG 3'
FAM210A-3xFlag-R (EcoRI): 5' CGGAATTCTTCCACTTTTTTCTTAAAGGAAAC 3'
Fam210a-EGFP-F (XhoI): 5' CCGCTCGAGCAAAATGCAATGGAATGTACCAC 3'
Fam210a-EGFP-R (EcoRI): 5' CGGAATTCGTTCCACTTTTTTCTTAAAGGAC 3'
The 2nd generation of lentivirus package vectors, psPAX2 and pMD2.G, were gifts from Dr. Guang Yang at City of Hope National Medical Center. pLV-CMV-EF1-GP vector was used for the construction of FAM210A overexpression plasmid. Lentivirus-based shRNA vectors against mouse (vector name: pLV[shRNA]-EGFP:T2A:Puro-U6>mFam210a[shRNA#1]) and human (vector name: pLV[shRNA]-EGFP:T2A:Puro-U6>hFAM210A[shRNA#1]) FAM210A were designed and cloned through VectorBuilder website, and purchased from the company. Primary antibodies used in this study are listed in the table in the last page.

The *mir*Vana® miRNA mimics are chemically modified double-stranded RNAs designed for *in* vivo injection studies in mouse models as well as *in vitro* transfection in cell culture studies (designed and synthesized by ThermoFisher Scientific). Scrambled *mir*Vana® miRNA mimics (Cat. No. 4464061) were used as a negative control. The mirVana miRNA mimics for mmu-miR-574-5p (Cat. No. 4464070), miR-574-3p (Cat. No. 4464066), and Silencer Select siRNA against mouse (Cat. No. 173500) or human (Cat. No. 129436) FAM210A were purchased from ThermoFisher Scientific. miRNAs were transfected into cells using lipofectamine 3000 (ThermoFisher Scientific) following the manufacturer's instructions. For AC16 cell transfection, 200 nM miRNA mimics (scrambled miRNA, miR-574-5p, or miR-574-3p) were transfected for 24 hrs before measuring hypertrophic growth of the cells. 100 nM scrambled siRNA or FAM210A siRNA and 1 μg of FAM210A overexpression plasmid were transfected for 24 hrs before measuring expression of ETC component proteins.

Total RNA was isolated from the mouse and human left ventricle samples using Trizol reagent (ThermoFisher Scientific). For RT-qPCR detection of miRNA expression, 1 μg of RNA was used for reverse transcription using the miScript II RT Kit (Qiagen, Cat. No. 218161). 1 μl of cDNA and miScript® SYBR® Green PCR kit (Cat. No. 218073) were used for quantitative PCR. qPCR primers were purchased from miScript Primer Assays (Qiagen): Hs_miR-574-5p_2 miScript Primer Assay (Cat. No. MS00043617), Hs_miR-574-3p_1 miScript Primer Assay (Cat. No.: MS00032025), Hs_SNORD68_11 miScript Primer Assay (Cat. No. MS00033712), Hs_RNU6_11 miScript Primer Assay (Cat. No. MS00033740). iScript™ cDNA Synthesis Kit (Bio-Rad, Cat. No. 1708891) was used for mRNA reverse transcription. iTaq™ Universal Probes Supermix (Bio-Rad, Cat. No.

1725134) and TaqMan Real-time PCR assays (ThermoFisher Scientific) were used for TaqMan qPCR assay. Taqman qPCR assay probes (mouse) were purchased from ThermoFisher Scientific, and the Cat. numbers are listed as follows: Fam114a1 (4351372, Mm00471781_m1), ANF (Nppa, Mm01255747_g1), BNP (Nppb, Mm01255770_g1), Myh6 (Mm00440359_m1), Myh7 (Mm01319006_g1), Col1a1 (Mm00801666_g1), Col1a2 (Mm00483911_g1), Col3a1 (Mm00802305_g1), Ctgf (Mm01192932_g1), Rpl30 (Mm01611464_g1), Gapdh (Mm99999915_g1). iTaq Universal SYBR Green Supermix (Bio-Rad, Cat. No. 1725125) and qPCR primers (synthesized from IDT) were used for SYBR green qPCR assay. The sequences of SYBR qPCR primers are listed as below (h: human, m: mouse, h/m: human and mouse).

qPCR procedure: 1) initial denaturation at 95°C for 60 sec. 2) 40 cycles of denaturation at 95°C for 10 sec and annealing/extension at 60°C for 45 sec. 3) melt curve analysis by 0.5°C increments at 5 sec/step between 65-95°C.

Gene	Forward Primer (5'-3')	Reverse Primer (5'-3')
hGAPDH	GGAGCGAGATCCCTCCAAAAT	GGCTGTTGTCATACTTCTCATGG
hFAM210A	AAAGTTCTGATTCCAGTGCAT	TGTTGCAATCTTAAACAAGGCAT
hMTND2	CCTCACTAAACGTAAGCCT	TTGTACGGTAGAACTGCTA
hMTND3	ATTTGATCTAGAAATTGCCCTC	TTGTAGTCACTCATAGGCCAG
hMTCYTB	TACACAATCAAAGACGCCCTC	AGGAAATATCATTCGGGCTT
hMTCO1	CAGGCTTCGGAATAATCTCCC	TATGCTCGTGTGTCTACGTCT
hMTATP6	CCTAGCCCACTTCTTACCAC	CGCTTCCAATTAGGTGCAT
hNDUFS1	TAACCTCTAAGCCCTATGCC	TGATGTCCTCATGCATACGTG
hSDHA	CAAACTCGCTCTTGGACCT	CAGTTCCGATGTTCTTATGCTT
hUQCRC2	GAATCCGCAGACTCATGTCA	AATCAAAGCCATTCTTGCACT
hCOX4i1	GCCTTGCTCTCTTCCGGTCG	AGTCTTCGCTCTTCACAACACT
hATP5B	GATCTGCTAGCTCCCTATGCC	CCAGCAAACACAGAGTAACCAC
mGapdh	AGGTCGGTGTGAACGGATTTG	TGTAGACCATGTAGTTGAGGTCA
mFam210a	ATCAGTCTGTATCAACGGTTT	TTTTCAGGATGTCCACGAT
mMtnd1	ACACTTATTACAACCCAAGAACACA	TCATATTATGGCTATGGGTCAGG
mMtnd2	TTTACCCGCTACTCAACTCT	TATTCATCCTATGTGGGCAAT
mMtnd5	GCCAACAACATATTTCAACTTTTC	ACCATCATCCAATTAGTAGAAAGGA
mMtcytb	ATAAAATTCCATTTCACCCCTA	TAGGAAATATCATTCGGGTT
mMtco1	CAGACCGCAACCTAAACACA	TTCTGGGTGCCCAAAGAAT
mMtatp6	ATTATTGAAACAATTAGCCTA	TTATTAATACTAGAGTAGCTCC
mNdufs1	TAGTTGGTACAAATCCACGTT	ATCCTTTAAGACCTCGCAGA
mSdha	CGCCTAAACATGCAGAAGTCG	CCAAACCATTCCCCTGTCG
mHibadh	CATCCTCTATGACGTGTTCCC	TGCTCCAGAATAAACTTCTACCG
mLdhb	CTTGTTCCTCCAGACTCCGAA	TTTCTCTGCACCAGGTTGAGC
mUqcrc2	TTATATGCCACCTTCTACCG	CGCATGTGTTATAACTCGTC
mCox4i1	CCGCATCCAGTTTAACGAGAGC	GCCACCCAGTCACGATCGAA
mAtp5b	ATGGAGCTAATCAACAATGTCG	CATCTGTCCATATACCAACGCTA

RN18S	TGACTCAACACGGGAAACCTC	CATGCCAGAGTCTCGTTCGTT
mCox1	CAGACCGCAACCTAAACACA	TTCTGGGTGCCCAAAGAAT
Mt-RNR1	GGATTAGATACCCCACTATGCT	CTGAAGATGGCGGTATATAGGC
mChr11	ATGGAAAGCCTGCCATCATG	TCCTTGTTGTTCAGCATCAC
hChr15	TGCTGTCTCCATGTTTGATGTATCT	TCTCTGCTCCCCACCTCTAAGT

Human specimens

All human samples of frozen cardiac tissues (n=23), including 17 samples from explanted failing hearts and 6 samples from non-failing donor hearts, were acquired from the Cleveland Clinic (Wu *et al*, 2020). This study was approved by the Material Transfer Agreement between the URMC and the Cleveland Clinic. All human samples were picked up randomly based on the presence or absence of heart failure by our collaborator, Dr. Waihong Wilson Tang at Cleveland Clinic. We have been blinded from any clinical data. Total RNA was extracted from human heart tissues using TRIzol reagent (ThermoFisher Scientific) following instructions in their manual. For the mRNA detection, 1 μg of total RNA was used as a template for reverse transcription assay using the iScript cDNA Synthesis Kit (Bio-Rad); cDNA was used for detecting FAM210A and GAPDH expression. For the expression of miRNA, the total RNA (containing small RNAs) was subjected to miRNA reverse transcription using the miScript II RT Kit (Qiagen), following the manual. The RT product was used for detection of miR-574-5p/3p and control RNA Snord68 using the miScript Primer Assay (Qiagen).

Mice

Three Sanger MirKO ES cell lines for Mir574 (produced by Wellcome Trust Sanger Institute)(Prosser *et al*, 2011) were purchased from the mutant mouse regional resource center (MMRRC). miR-574$^{-/-}$ global knockout chimera founder mice were generated in the Case Western Reserve University Transgenic Core Facility (Dr. Ronald Conlon). We generated a miR-574 targeted male chimera mouse in the C57BL/6J background, performed germline transmission, and backcrossed the mice to C57BL6J mice (purchased from Jackson Laboratory) for more than 10 generations. A 182-bp DNA region was deleted including the microRNA574 gene (96-bp for pre-miR-574 sequence) in the intron 1 of the host gene Fam114a1. The puromycin resistance cassette was removed by breeding with C57BL/6-Tg(Zp3-cre)93Knw/J mouse (ZP3-Cre transgene), which expresses Cre recombinase in oocytes, resulting in a null allele. For experiments with miR-574$^{-/-}$ mice, control mice of the same age and gender from littermates or sibling mating were used. All animal procedures were performed in accordance with the National Institutes of Health (NIH) and the University of Rochester Institutional guidelines. This miR-574$^{-/-}$ mouse line has been deposited in JAX laboratory (https://www.jax.org/strain/031919).

Genotyping PCR and electrophoresis protocols are shown as below (miR-574 KO mice and genotyping protocols were donated to MMRRC):

Reagent/Constituent	Volume (μL)
Water	17. 5
10x Buffer	2.5
$MgCl_2$ (stock concentration is 25 mM)	1
dNTPs (stock concentration is 10 mM)	1
Primer 1 (stock concentration is 20 μM)	0.25
Primer 2 (stock concentration is 20 μM)	0.25
Taq Polymerase 5 Units/μL	0.5
DNA (50-200 ng/μL)	2
**TOTAL VOLUME OF REACTION:**	**25 μL**

Steps	Temp (°C)	Time (m:ss)	# of Cycles
1. Initiation/Melting	94	5:00	1
2. Denaturation	94	0:30	
3. Annealing	56	0:30	34x
4. Elongation	68	0:30	
5. Amplification	68	5:00	1
6. Finish	4	∞	n/a

Name	Nucleotide Sequence (5' - 3')	Agarose: 2% V: 125		
1. Mir574_F1	GGGAACCGTCCCGAGAAAGCAACTT	Estimated Running Time: 45-50 min		
2. Mir574_R1	AACTTTCCAGACTGGCTTGTGCCTA	**Primer Combination**	**Band**	**Genotype**
3. n/a	n/a	F1/R1	230 bp	WT
4. n/a	n/a	F1/R1	230 & 203 bp	HET
5. n/a	n/a	F1/R1	203 bp	HOMO

Isoproterenol (ISO) injection and infusion model

Experimental mice are siblings generated from intercrosses of miR-574$^{+/-}$ mice. Age and background matched WT and miR-574$^{-/-}$ female mice at 10-12 weeks of age were subjected to vehicle (saline) or ISO treatment. Procedure for ISO injection for the ISO treatment model (Fig 2): ISO or vehicle saline were administered to WT and miR-574$^{-/-}$ mice daily for 4 weeks using subcutaneous injection (30 mg/Kg/day). Excised mouse hearts were flushed with saline to remove the blood, fixed in 10% formalin, and used for histological and immunoblotting analyses. Procedure for mini-osmotic pump implantation for the rescue model (Appendix Fig S5): Mouse was anesthetized using 2.0% isoflurane and placed on a heated surgical board. A side/upper back area skin incision was made, and a mini-osmotic pump was inserted subcutaneously set to deliver ISO or vehicle. The incision was then closed with 6-0 coated vicryl sutures in a subcuticular manner, and the animals were allowed to recover. The pumps were not removed and will remain for a time period of 4 weeks, after which the animals were euthanized. The sutures were removed after 2 weeks since the pumps were transplanted.

Transverse aortic constriction (TAC) surgical model

Experimental mice were siblings generated from intercrosses of miR-574$^{+/-}$ mice. Age and background matched WT and miR-574$^{-/-}$ male mice at 10-12 weeks of age were subjected to Sham or TAC surgery. Each mouse was anesthetized using 2.0% isoflurane, placed on a surgical board with a heating pad (half inch plexiglass between the animal

and the heating pad), and given buprenorphine SQ. A midline cervical incision was made to expose the trachea for visualizing oral intubation using a 22-gauge (PE90) plastic catheter. The catheter was connected to a volume-cycled ventilator supplying supplemental oxygen with a tidal volume of 225-250 μl and a respiratory rate of 120-130 strokes/min. Surgical plane anesthesia is subsequently maintained with 1-1.5% isoflurane. Procedure for left thoracotomy: Skin was incised, and the chest cavity was opened at the level of the 2nd intercostal space. Transverse section of the aorta was isolated. Transverse aortic constriction was created by placing a (6-0 silk) ligature securely around the trans-aorta and a 27-gauge needle, causing complete occlusion of the aorta. The needle was removed, restoring a lumen with severe stenosis. Lungs were reinflated and the chest was closed using Vicryl 6-0 suture. Muscle and skin were sutured using a Vicryl 6-0 suture in a running subcuticular pattern. Once the mouse was breathing on its own, it was removed from the ventilator and allowed to recover in a clean cage on a heated pad.

The mice were randomized for experiments using simple randomization with a specific ID number before animal procedures. All animal operations, including ISO infusion, TAC surgery, and echocardiography measurement, were performed by the Microsurgical Core surgeons. Sections and histology analysis were done by the Histology Core. The technicians from both Microsurgical Core and Histology Core were all blinded to the genotypes of the mice and tissue samples. For group size justification, we performed the power analysis using both G*power (Faul *et al*, 2007) version 3.1.9.6 and the function of power.anova.test in R version 3.5.3 (R Foundation for Statistical Computing, Vienna, Austria). The assumptions include the same standard variance in each study group, effect size=$\frac{\text{Difference of the means between study groups}}{\text{common standard deviation}}$, alpha level=0.05, power=0.9 and number of study groups. The effect size for specific experiments is assumed based on previous similar studies or literature. In previous experiences from our Microsurgical Core, we have observed a survival rate of ~90% after the TAC procedure. To offset the possible loss of mice per treatment, we added at least one mouse per treatment group.

In vivo therapeutic model and rescue model using miRNA mimics

Based on recommendations from the nanoparticle user instruction, 75-100 μg miRNA with chemical modification (resistant to nuclease degradation *in vivo*) needs to be used per injection. In our experiments, miRNA mimics were used at the dose of 5 mg/Kg body weight in the volume of 150-200 μl for injections in male WT C57BL/6J mice (TAC model) or miR-574 KO mice (ISO model) (10-12 weeks old). The *mir*Vana® miRNA mimics for miR-574-5p/3p and scrambled *mir*Vana® miRNA mimics (negative control) (~100 μg) were dissolved in ~150-200 μl RNase-free water. The diluted miRNA mimics were incubated with 50 μl of nanoparticle-based in vivo transfection reagent (Altogen Biosystems, Cat. No. 5031) in sterile tubes for 20 mins at RT. Transfection enhancer (10 μl, Altogen Biosystems, Cat. No. 1799) was added to the mixture, vortexed gently, and incubated for 5 mins at RT. The nanoparticle-miRNA mimics complex was mixed with an appropriate volume of the sterile solution of 5% glucose (w/v), and delivered into the murine heart by intravenous tail vein injections (after mice are anesthetized using 2.0% isoflurane) once a week after TAC surgery following the manufacturer's instruction and a previous report (Wang *et al*, 2016).

In the rescue model, miR-574 KO mice (2 months old) were subcutaneously infused with Azlet osmotic minipumps filled with either saline or ISO (20 mg/Kg body weight) for 28 days. After 3 days of infusion, mice were injected with nanoparticle carrying miR-574-3p, miR-574-5p, both combined, or scrambled miRNA mimics (5 mg/Kg body weight) through the tail vein on day 4, 10, 16, and 22 using a 1 mm BD syringe. After the treatment period, mice were euthanized, and tissues were flash frozen and stored at -80°C until further analysis.

Echocardiography

Echocardiographic image collection was performed using a Vevo2100 echocardiography machine (VisualSonics, Toronto, Canada) and a linear-array 40 MHz transducer (MS-550D). Image capture was performed in mice under general isoflurane anesthesia with heart rate maintained at 500-550 beats/min. LV systolic and diastolic measurements were captured in M-mode from the parasternal short axis. Fraction shortening (FS) was assessed as follows: % FS = (end diastolic diameter - end systolic diameter) / (end diastolic diameter) x 100%. Left ventricular ejection fraction (EF) was measured and averaged in both the parasternal short axis (M-Mode) using the tracing of the end diastolic dimension (EDD) and end systolic dimension (ESD) in the parasternal long axis: % EF=(EDD-ESD)/EDD. Hearts were harvested at multiple endpoints depending on the study.

Langendorff procedure for adult cardiomyocyte/fibroblast isolation:

Preparation: 10x perfusion buffer (pH 7.4) was prepared and stored at 4°C the day before the procedure. Coverslips were sterilized with 70% EtOH for 15 mins, then rinsed with 100% EtOH, and air-dried overnight. On the day of operation, TC plates were coated with laminin for cardiomyocytes (CMs) for >2 hrs at 37°C. The perfusion apparatus was turned on, warmed up, washed by Milli Q H_2O, 70% EtOH, Milli Q H_2O, and rinsed with 200 ml Milli Q H_2O before the first Langendorff of the day. Fresh perfusion buffer (PB) and digestion buffer (DB) was made and sterilized through 0.2 μm filters. The perfusion apparatus was filled with 1x PB, and a 10 cm petri dish was filled with 1x PB to place the heart before cannulation. Then, the empty 15-ml Falcon with 100 μm mesh was prepared.

Protocol: Mice were anesthetized with anesthetic solution (0.5 ml ketamine [100 mg/Kg]/ xylazine [10 mg/Kg]/PBS + 0.2 ml 1000 U/ml heparin), and sacrificed by cervical dislocation. Hearts were removed, cannulated and perfused with 1x PB for 4 mins, switched to DB for 3 mins, and then perfused again with 32 ml DB with $CaCl_2$ for 8 mins. Hearts were then released from cannula and atria, and the great vessels were removed. Ventricles were placed in sterile 35 mm dish with 2.5 ml DB and shredded into several pieces with forceps. 5 ml stopping buffer (SB) was added and pipetted several times until the tissues disperse readily and the solution turned cloudy. The cell solution was transferred to a conical tube with 100 μm mesh to filter the cells from tissue. Dishes were rinsed with 2.5 ml SB and added to a tube through a mesh (final volume is 10 ml). CMs were settled by incubating the tube at 37°C for 15 mins. The supernatant (containing cardiac fibroblasts) was transferred to a fresh tube (~8 ml), and 10 ml SB was added to the pellet of CMs. CMs were treated with Ca^{2+} ramp, swirled to mix every 2-3 mins, followed by adding 10 μl 100 mM Ca^{2+} for 2 mins, 40 μl 100 mM Ca^{2+} for 4 mins, and 90

μl 100 mM Ca^{2+} for 7 mins. The laminin was removed from plates and CMs were added in final SB with Ca^{2+}. Plates were centrifuged for 5 mins at 1,000 rpm at 4°C. CMs sat at 37°C for ~1 hr in SB, and then were washed 3 times with 2 ml 1x PBS. CMs were washed 3 wells at a time by swirling 3 times followed by tilting, aspirating, and replacing with 1x PBS. One adult heart should yield at least 2-3 full 6 well plates of CMs. We aimed for less than 10% dead and round CMs. Cardiac fibroblasts (CFs) from the supernatant were pelleted for 5 mins at 1000 rpm at 4°C. CFs were plated in 4-5 ml CF-specific media (DMEM with 10% FBS and 1% penicillin/streptomycin) in 60 mm plate. CFs were washed vigorously 3-5 times with 2 ml 1x PBS after 2-3 hrs, and replaced with fresh CF media.

Solutions for Langendorff operations:

10x perfusion buffer (stored at 4°C up to 1 month)

	500 ml
NaCl	35.15 g
KCl	5.5 g
KH_2PO_4	0.41 g
Na_2HPO_4	0.425 g
$MgSO_4•7H_2O$	1.5 g
HEPES (1M)	50 ml
pH to 7.4 with 1N NaOH	~3 ml
Milli Q H_2O	to 500 ml

1x perfusion buffer (PB) (make fresh)

	500 ml
10x perfusion buffer	50 ml
$NaHCO_3$	0.195 g
Taurine	1.875 g
BDM (butadione monoxime)	0.5 g
Glucose	0.5 g
pH to 7.4 with NaOH	
Milli Q H_2O	to 500 ml
Filter through 0.2 μm	

Stopping buffer (SB) (20 mL/heart, make fresh, heat to 37°C before use)

	x1	x2	x5
1x PB	9 ml	18 ml	45 ml
FBS	1 ml	2 ml	5 ml
100 mM $CaCl_2$	1.25 μl	2.5 μl	6.25 μl

Digestion buffer (DB)

1x PB	50 ml
305 U/mg collagenase II	50 mg
100 mM $CaCl_2$	15 μl

Cardiac fibroblast culture medium

DMEM	445 ml
Penicillin/Streptomycin	5 ml (1% final)
FBS	50 ml (10% final)

Cardiomyocyte culture Medium

MEM + Earle's salts + L-glutamine (Corning MT 10-010CV)	99 ml
0.2% BSA (Sigma A9647)	0.1 g
10 mM HEPES (HEPES, not Na-HEPES, Santa Cruz sc29097)	0.24 g
4 mM $NaHCO_3$	0.084 g
10 mM creatine monohydrate (Sigma C3630)	0.149 g
1% Penicillin/Streptomycin	1 ml
TOTAL..	100 ml
pH to 7.4 with 10N NaOH, then filter sterilize through 0.2 μm filter	
0.5% Insulin-Selenium-Transferrin (1:200 dilution; add before use)	50 μl per 10 ml CM media
Blebbistatin	10 μl per 10 ml CM media

Neonatal mouse cardiac myocyte isolation, cell culture and treatment

Neonatal primary cardiomyocytes were isolated from C57BL6J mouse pups (1-2 days old). Briefly, hearts were excised, and ventricles were separated and rinsed in phosphate-buffered solution (PBS) before digestion with multiple steps of collagenase type II digestion buffer. 50 ml digestion solution was prepared in PBS pH 7.4. 40 mg collagenase (Collagenase Type II from Worthington #4176, 305 U/mg) was added to 50 ml PBS (244 U/ml) and filter sterilized (0.22 μm). 2 ml digestion buffer was aliquoted into a 15-ml conical and stored on ice for isolation. The cells were collected by centrifugation and re-suspended in Dulbecco's Modified Eagle Medium (DMEM, high glucose) containing 10% fetal bovine serum (FBS), 5% of penicillin-streptomycin, 1% L-Gln. Non-myocytes, which largely consist of fibroblasts were removed by plating the cells at 37°C for 1 hr. The medium was then carefully transferred to another 10 cm dish for the fibroblast attachment for 1 hr. After that, the medium was collected and centrifuged at 1000 rpm for 5 mins, and the pellet was suspended in fresh DMEM medium and transferred to 0.1% gelatin-coated plates (pre-coated for 1 hr at 37°C). After 24 hrs of culturing, cells were transfected with miR-574-5p/3p mimics and negative control miRNA using Lipofectamine 3000 followed by ISO treatment (10 mm) for 24 hrs. After that, cells were fixed with 4% paraformaldehyde (PFA) for 10 mins at room temperature, washed with PBS (3x 5 mins), and permeabilized using 0.05% Triton X-100 in PBS for 5 mins. The permeabilized neonatal cardiomyocytes were stained with Phalloidin 484 FITC (1:1000 dilution) for 1 hr at room temperature, washed with PBS (3x 5 mins), and finally incubated with DAPI to stain the nuclei. The images were captured using confocal microscopy. A yield of ~3 million CMs for ~8 pups was expected.

Detailed procedure of neonatal primary cardiomyocytes isolation: Pups were placed on ice and incubated for 5-10 mins, and then decapitated with the midline carefully cut down. The body was pinched to expose the heart. Forceps were used to remove and transfer the heart to a conical with 10 ml DMEM(-). The hearts were mixed gently by decanting ~5

ml media to waste, and then poured into a 6-cm plate. The atria/AV canals were removed using Weckel scissors. The media was removed using a 5-ml serological pipet. 5 ml fresh DMEM(-) was added to rinse and remove. 2 ml digestion solution was added to rinse and removed, and 2 ml of fresh digestion media was added. The hearts were diced/minced using Weckel scissors. 6 ml digestion solution was added to the minced hearts in a cell culture hood. The digestion was transferred to a sterile glass vial (i.e., small scintillation vials) with a small stir bar, and incubated for 10 mins at 37°C with stirring. The 1st digestion was discarded. 10 ml digestion solution was added to stir for 10 mins at 37°C, and was allowed to settle. The digestion debris was transferred to 10 ml of culture medium on ice followed by centrifugation at 1,500 rpm for 3 mins at 4°C. The supernatant was removed, and the pellet was re-suspended in 5 ml media on ice. The digestion step was repeated 3 times. Each digestion was pooled and filtered through a 70 μm strainer into a new conical and spun at 1,500 rpm for 3 mins at 4°C. The supernatant was removed and 5 ml culture medium was added to suspend cells. A 10-cm culture dish was pre-plated with suspended cells for 2 hrs. Cardiac fibroblasts will adhere to the uncoated cell culture dish. The adherent cells (fibroblast fraction) can be saved for culture, RNA, or protein analysis. The supernatant (myocyte fraction) was transferred to a new 15 ml sterile conical Falcon centrifuge tube. The plate was washed once with 5 ml culture medium, and the wash was added to the supernatant. Cardiomyocyte cells were counted and plated on 0.1% gelatin-coated dishes or plates (gelatin coating for 2 hrs; 12-well plate: 4 x 10^5 cells/well).

Cell culture

AC16 adult human ventricle cardiomyocyte cells, primary mouse neonatal and adult cardiomyocytes, primary mouse adult cardiac fibroblasts, and HEK293T cells were used to address questions at the cellular and molecular level. Both cells were cultured following the manual (Sigma, Cat. No. SCC109 for AC16, SCC065 for HL-1). AC16 cells were cultured in DMEM/F12 containing 2 mM L-Glutamine, 12.5% FBS, and 1x Penicillin/Streptomycin Solution. For myocyte hypertrophy experiment, AC16 cells were treated with 10 μM ISO for 24 hrs. HEK293T cells were cultured in DMEM containing 2 mM L-Glutamine, 10% FBS, 1x Penicillin/Streptomycin Solution. The cells were tested for mycoplasma contamination and shown negative signals. Cell culture methods are described above for primary mouse neonatal and adult cardiomyocytes, and primary mouse adult cardiac fibroblasts.

Cardiac fibroblast cell proliferation and activation assay

Primary adult mouse cardiac fibroblast (ACF) cells were isolated as described above in the Langendorff procedure (Wu *et al.*, 2020). The P1 generation of ACF was used for the cell proliferation and activation assay to avoid auto-activation during cell culture. Cyquant Cell Proliferation Assay kit (ThermoFisher Scientific, C7026) was used to measure the ACF cell proliferation rate following the manual. Briefly, the same number of ACF cells were seeded in the 24-well plate and transfected with 100 nM negative control (NC) miRNA, miR-574-5p or miR-574-3p mimics. After miRNA transfection, the cells were serum starved for 12 hrs followed by treatment with 10 ng/mL mouse TGF-β for 24 hrs. The wells were washed with PBS buffer and frozen at -80 °C for 1 hr. The plate was then thawed at room temperature and 200 μl of the CyQUANT GR dye/cell-lysis buffer was

added into each well. The samples were incubated at room temperature for 5 mins. The cell number was determined by fluorescence microplate reader with 480 nm excitation and 520 nm emission. For the activation assay, the ACF cells were treated the same as cell proliferation assay. The cells were lysed in RIPA buffer after TGF-β treatment and the samples were subjected to immunoblot to detect α-SMA expression.

Luciferase activity assay

Reporter vectors were generated by inserting the Fam210a 3'UTR fragments containing wild-type or mutated seed sequence into the miRNA reporter vector pmirGlo (Promega). All the primers used to generate the reporter vector were listed as below. The wild-type 3'UTR fragment was amplified and ligated to pmirGlo. Then, 100 ng of wild-type or mutant reporter vectors were co-transfected with 20 nM miRNA (miR-574-5p or miR-574-3p) into HEK293T cells cultured in 24-well plate using lipofectamine 3000 (ThermoFisher Scientific) following the manufacturer's instruction. Cells were collected 36 hrs after transfection. Additionally, firefly and renilla luciferase activity were detected by the Dual-Luciferase Reporter Assay System (Promega).

Fam210a-UTR-3p-seed-F: 5' CTAGCTAGCAGTCTCAGAGCATGGCTCCTTA 3'
Fam210a-UTR-3p-seed-R: 5' ACGCGTCGACGGATGATGCAGAGATGCTGTTG 3'
Fam210a-UTR-3p-seed-Mu-F: 5' CCAACTTCCGAGGGCCTGAGGCTTCC 3'
Fam210a-UTR-3p-seed-Mu-R: 5' GAGCGTGAGAGTTTGCACGCTTGACC 3'
Fam210a-UTR-5p-seed1-F: 5' CTAGCTAGC ATCTTTAAGGCAAGAAACAGCT 3'
Fam210a-UTR-5p-seed1-R: 5' ACGCGTCGAC CCCTTTGGTGAAACTGAACAGC 3'
Fam210a-UTR-5p-seed1-Mu-F: 5' GTGATGTCCTAAACGGAGCTGTGACT 3'
Fam210a-UTR-5p-seed1-Mu-R: 5' AGAAACTTTCAAAGGATGGATATGCT 3'
Fam210a-UTR-5p-seed2-F: 5' CTAGCTAGCCCTGTGTTAGAGCTCAGGGTTG 3'
Fam210a-UTR-5p-seed2-R: 5' ACGCGTCGACAATAACCTGTTCCCTGGGGTTG 3'
Fam210a-UTR-5p-seed2-Mu-F: 5' GTGTGACCAATATTTTAAAGCCAGTA 3'
Fam210a-UTR-5p-seed2-Mu-R: 5' AGACTGCACACGCACACACATGTGTG 3'

Mitochondria isolation and mitoplast purification

Mitochondria isolation and mitoplast purification from AC16 cells and murine hearts were performed as previously described (Pallotti & Lenaz, 2007). The cells were collected by centrifugation and re-suspended in ice-cold mitochondria isolation buffer (3 mM HEPES-KOH pH7.4, 210 mM mannitol, 70 mM sucrose, 0.2 mM EGTA with protease inhibitor cocktail). Then, the cells were homogenized by a pre-chilled Micro-Tube Homogenizer (Bertin Technologies) for 40-50 strokes. The homogenized cell samples were centrifuged at 500 g for 10 mins to remove the nuclei and unbroken cells. The mitochondria were sedimented by centrifugation at 10,000 g from the clear supernatant. To isolate the mitoplast and outer membrane (OM), we used mitochondria isolation buffer containing 0.5 mg/ml digitonin (Sigma) to re-suspend the mitochondria pellet and incubate at 4°C in microtube mixer for 15 mins. The mitoplast was sedimented by 20,000 g centrifugation for 10 mins after adding an equal volume of mitochondria isolation buffer. The pellet contained the matrix and inner membrane (IM) while the supernatant was the OM fraction

of the mitochondria. Western blot analysis of OM (TOM20) or IM ETC (ATP5B) protein marker indicates the quality of purification.

Interactome capture by Immunoprecipitation-Mass spectrometry (IP-MS)

1. Immunoprecipitation: The total mitochondria were isolated from the whole heart of wild-type mice following the manual using the Mitochondria Isolation Kit for Tissue (Pierce). The mitochondria pellets were re-suspended in NP-40 lysis buffer (50 mM HEPES, pH 7.5, 150 mM KCl, 0.5% NP-40, 2 mM EDTA, 1 mM NaF, 0.5 mM DTT with proteinase inhibitor cocktail), and incubated on ice for 15 mins. The mitochondrial lysate was centrifuged at 12,000 rpm for 10 mins. The suspension was equally divided into two parts and incubated with 1 μg rabbit pre-immune IgG or anti-FAM210A rabbit polyclonal antibody at 4°C with rotation overnight, respectively. The protein-antibody complex was pulled down by incubation with Dynabeads Protein G (ThermoFisher Scientific) for 4 hrs and eluted using 1x SDS loading buffer. For the mass spectrometry assay, the elution was loaded onto 4-12% gradient SDS-PAGE gel and ran for 10 mins. The whole lane was subjected for mass spectrometry analysis by the Mass Spectrometry Resource Lab of University of Rochester Medical Center. The interaction of FAM210A and EF-Tu or ATAD3A was confirmed by Western blot following IP using indicated antibodies.

2. Sample Preparation: For mass spectrometry experiments, samples were run into a 4-12% SDS-PAGE gel for a short period of time to remove contaminants and create a ~10 mm length region, allowing the total protein to be evaluated in a single gel digest. After staining with SimplyBlue SafeStain (Invitrogen), these regions were excised, cut into 1 mm cubes, de-stained, then reduced and alkylated with DTT and IAA, respectively (Sigma). Gel pieces were dehydrated with acetonitrile. Aliquots of trypsin (Promega) were reconstituted to 10 ng/μl in 50 mM ammonium bicarbonate and added so that the solution was just covering the dehydrated gel pieces. After 0.5 hr at room temperature (RT), additional ammonium bicarbonate was added until the gel pieces were completely submerged and placed at 37°C overnight. Peptides were extracted the next day by adding 0.1% TFA, 50% acetonitrile, then dried down in a CentriVap concentrator (Labconco). Peptides were desalted with homemade C18 spin columns, dried again, and reconstituted in 0.1% TFA.

3. FAM210A-IP LC-MS/MS: Peptides were injected onto a homemade 30 cm C18 column with 1.8 μm beads (Sepax), with an Easy nLC-1000 HPLC (ThermoFisher Scientific), connected to a Q Exactive Plus mass spectrometer (ThermoFisher Scientific). Solvent A was 0.1% formic acid in water, while solvent B was 0.1% formic acid in acetonitrile. Ions were introduced to the mass spectrometer using a Nanospray Flex source operating at 2 kV. The gradient began at 3% B and held for 2 mins, increased to 30% B over 41 mins, increased to 70% over 3 mins and held for 4 mins, then returned to 3% B in 2 mins and re-equilibrated for 8 mins, for a total run time of 60 mins. The Q Exactive Plus was operated in a data-dependent mode, with a full MS1 scan followed by 10 data-dependent MS2 scans. The full scan was done over a range of 400-1400 m/z, with a resolution of 70,000 at m/z of 200, an AGC target of 1e6, and a maximum injection time of 50 ms. Ions with a charge state between 2-5 were picked for fragmentation. The MS2 scans were performed at 17,500 resolution, with an AGC target of 5e4 and a maximum injection time

of 120 ms. The isolation width was 1.5 m/z, with an offset of 0.3 m/z, and a normalized collision energy of 27. After fragmentation, ions were put on an exclusion list for 15 seconds to allow the mass spectrometer to fragment lower abundant peptides.

4. Data Analysis: Raw data from MS experiments was searched using the SEQUEST search engine within the Proteome Discoverer software platform, version 2.2 (ThermoFisher Scientific), using the SwissProt human database. Trypsin was selected as the enzyme allowing up to 2 missed cleavages, with an MS1 mass tolerance of 10 ppm. Samples run on the Q Exactive Plus used an MS2 mass tolerance of 25 mmu. Carbamidomethyl was set as a fixed modification, while oxidation of methionine was set as a variable modification. The Minora node was used to determine relative protein abundance between samples using the default settings. Percolator was used as the FDR calculator, filtering out peptides which had a q-value greater than 0.01.

Lentivirus preparation and transfection

The lentivirus was produced using the 2nd generation system following an established protocol.[6] Briefly, the three vectors were transfected into HEK293T cells on the first day, and collected two rounds of the supernatant at 48 hrs and 72 hrs. The supernatant was centrifuged at 2,000 g for 15 mins to remove the cell debris. The lentivirus was concentrated by centrifugation at 19,000 rpm for 2 hrs, and the pellet was dissolved in DMEM medium. For the infection, the lentivirus was added to the medium with 8 μg/ml polybrene (Santa Cruz Biotechnology) and changed back to normal cell culture medium after 24 hrs. The cells were harvested after 48 hrs and knock-down or overexpression efficiency was measured.

RNA Northern blot analysis

RNA Northern blot analysis was performed following a previous protocol (Jafarifar *et al*, 2011). Total RNA (200 μg) was mixed with an equal volume of 2x RNA gel loading buffer (Ambion), heated at 80°C for 5 mins, ice-cooled, and ran through 15% denaturing acrylamide gel electrophoresis. Total RNA was transferred to Hybond N+ (GE Healthcare) by capillary blotting using 20x SSC buffer for overnight and then fixed using a UV crosslinker. miRCURY LNA detection probes (10 pmol, Exiqon) complementary to miR-574-5p or miR-574-3p were radio-labeled with T4 polynucleotide kinase (NEB) and 1 μl [γ-^{32}P] ATP for 1 hr at 37°C. Labeled LNA probes were heated at 95°C for 2 mins, ice-cooled, diluted in 50°C pre-warmed PerfectHyb Plus hybridization solution (Sigma) with denatured 20 μg/ml salmon sperm DNA (Ambion), and then added to the UV crosslinked pre-hybridized membrane at 50°C for 4 hrs. Membranes were washed 3 times in 2x SSC, 0.1% SDS at 50°C. The Decade Marker was used to indicate the molecular weight (Ambion). The membranes were stripped with boiled 0.1% SDS, 5 mM EDTA for 30 mins, and re-probed with radio-labeled LNA-modified U6 as a loading control.

Immunofluorescence and confocal microscopy

Immunostaining of cells grown on coverglass or chambered slides: HEK293T, AC16 cells, primary CMs were grown on the coverslips, and incubated with 100 nM MitoTracker Red CMXRos (ThermoFisher Scientific) for 30 mins at 37°C before being fixed for 10 mins

with 4% paraformaldehyde in PBS. Cells were washed with PBS for 3x 5 mins and permeabilized using ice-cold 0.5% Triton X-100 in PBS for 5 mins. After blocking with 1% BSA in PBS, the coverslips were incubated with indicated primary antibodies (anti-FAM210A 1:1000; anti-Flag 1:2000) in blocking solution (2% BSA in PBS) for 1 hr at RT and then washed with PBS for 3x 5 mins. The coverslips were incubated with the Alex Fluor-488 conjugated secondary antibodies (ThermoFisher Scientific, 1:1000) in PBS and washed with PBS for 3x 5 mins. Coverslips were air dried and placed on slides with antifade mounting medium (containing DAPI). The slides were imaged using an Olympus FV1000 confocal microscope. A negative control using the same amount of secondary antibody only was performed for IF or IHC to confirm the fluorescence signals as genuine target staining signals and not non-specific background.

Wheat germ agglutinin (WGA) and phalloidin staining

WGA (5 mg) was dissolved in 5 ml of PBS (pH 7.4). We performed deparaffinization by following steps: **a.** Xylene (100%) for 2x 5 mins; **b.** Ethanol (100%) for 2x 5 mins; **c.** Ethanol (95%) for 1x 5 mins; **d.** ddH_2O for 2x 5 mins. The slides were kept in a pressure cooker for 10 mins along with citrate buffer (10 mM, pH 6.0) for antigen retrieval. We quenched the slides with 0.1 M glycine in phosphate buffer (pH 7.4) for 1 hr at RT. Circles were made with a Dako pen, and slides were blocked with goat normal serum for 30 mins. 10 μg/ml of WGA-Alexa Fluor 488 (Sigma Aldrich) was applied to the slides for incubation for 1 hr at RT. Slides were rinsed in PBST wash buffer 3x 5 mins followed by PBS for 5 mins. A coverslip was placed on the slides with an antifade solution (containing DAPI) for imaging. Five different cross-sectional areas were selected, and the cell size of at least 500 CM cells were measured per area.

For primary murine CM cell culture, Alexa Fluor™ 594 Phalloidin (ThermoFisher Scientific, Cat. No. A12381) was used to measure the cell size following the instruction from the manual. Primary CM cells were treated with 10 mM ISO for 24 hrs for measuring cell size using Phalloidin. Additionally, primary CM cells were treated with 10 μM ISO for 48 hrs for the Trypan blue staining and TUNEL assay. Cultured CMs were fixed using a 4% paraformaldehyde in PBS for 10 mins, washed with PBS, and permeabilized using 0.2% Triton X-100 for 10 mins. Cells were blocked in 2% BSA/PBS for 1 hr and stained with Alexa Fluor™ 594 Phalloidin in 1:1000 dilution for 30 mins at RT. The stained cells were gently washed with PBS for 3x 5 mins, and the slides were mounted using a mounting medium with DAPI.

Picrosirius red staining

Paraffin-embedded tissue sections were deparaffinized and incubated in a picrosirius red solution (Abcam, Cat. No. ab150681) at RT for 1 hr. Then, slides were subjected to 2 washes of 1% acetic acid and 100% of ethyl alcohol, and then were mounted in a resinous medium. Images were captured using the PrimeHisto XE Histology Slide Scanner (Carolina). Six images were selected from each group for analysis. Total collagen content was determined for the whole heart images using an Image J software.

Trypan blue staining

Isolated primary adult CMs from mice were cultured in 40-mm glass dishes, treated with 10 μM of ISO for 24 hrs, and stained with Trypan blue for the assessment of cell viability of CMs. CM viability was analyzed by trypan blue dye exclusion assay. In brief, CM cultured medium was removed, and 0.04% (w/v) of trypan blue solution (VWR) was added for incubation at RT for 3-4 mins. The dead CMs appeared blue in color. CMs were visualized under the microscope. For each experiment, a total of 200 CMs were analyzed from different fields and dishes. CMs excluded from the trypan blue dye were considered to be viable cells. The percentage of viability was calculated.

Terminal deoxynucleotidyl transferase dUTP nick end labeling (TUNEL) staining

The tissue sections were washed with PBS twice and fixed using 4% paraformaldehyde for 20 mins. Cells were permeabilized using 0.5% Triton X-100 for 5 mins, and incubated in TUNEL reaction mixture (In Situ Cell Death Detection Kit; Sigma, 11684795910) for 1 hr at 37°C in the dark. Finally, cells were washed with PBS for 3x 5 mins, air dried, and mounted with DAPI-containing antifade medium. Images were captured using a BX51 microscope (Olympus).

Determination of cellular ATP content

The cellular ATP content was determined using a Molecular Probes ATP Determination Kit (ThermoFisher Scientific, A22066) according to the manufacturer's instructions. Briefly, cells (or heart lysates) were incubated in 1% (w/v) Trichloroacetic acid and centrifuged at 10,000 rpm for 5 mins at 4°C. The supernatant was collected and neutralized by Tris-HCl (0.1 M, pH 9.0), then mixed with the bioluminescent reagent. After incubation, bioluminescent signals were read using the HTX microplate reader (BioTek Instruments). ATP concentrations were determined in all samples based on a standard curve and normalized by the total protein mass.

Measurement of mitochondrial membrane potential

The mitochondrial membrane potential was measured using TMRE (Tetramethylrhodamine ethyl ester, ThermoFisher Scientific, Cat. No. T669) according to the manufacturer's protocol. Isolated adult mouse CMs were loaded with TMRE at 25 nM concentration for 30 mins at RT. After incubation, cells were washed with 1x PBS for 3 times, and submerged in live cell imaging solution. Fluorescence images were captured using a laser scanning confocal microscope (Olympus).

Dihydroethidium (DHE) staining

DHE staining was performed in both isolated live murine cardiomyocyte cells and frozen heart tissue sections for detection of intracellular reactive oxygen species (ROS). Primary adult CMs were isolated from WT and miR-574$^{-/-}$ mice and cultured using the standard procedure. Also, heart sections were prepared by the frozen section procedure from WT and miR-574$^{-/-}$ mice. In situ superoxide radical's production in live CMs and frozen sections was measured with the oxidative fluorescent dye called Dihydroethidium (DHE, Thermofisher Scientific). Live CM cells were plated and then treated with vehicle (0.5% DMSO) and ISO for 24 hrs. The cell or heart tissue sections were incubated with 10 μM DHE for 20 mins at 37°C in the dark and washed with PBS for 3x 15 mins. Sections were

air-dried and mounted with a coverslip. Images were captured using an Upright digital immunofluorescence microscope (BX51, Olympus). Four images were taken from each section. Fluorescence signal intensities were quantified with an Image J software.

Transmission electron microscopy

Murine hearts from 8-10 week old male mice (treated with vehicle or ISO injection at 30 mg/Kg/day for 4 weeks) were flushed with saline containing heparin for 10 seconds. Heart slices were cut and immediately fixed in 2.5% glutaraldehyde buffered with 0.1 M sodium cacodylate in a flat bottom tube. The fixative solution needs to be stored at 4ºC and balanced to RT before use. Samples were post-fixed in 1% osmium tetroxide, dehydrated in ethanol, transitioned into propylene oxide, and then transferred into Epon/Araldite resin. Tissues were embedded into molds containing fresh resin and polymerized for 2 days at 65°C. 70 nm sections were placed onto carbon-coated Formvar slot grids and stained with aqueous uranyl acetate and lead citrate. The grids were examined using a Gatan 11 megapixel Erlansheng digital camera and Digital Micrograph software. After obtaining the TEM images, mitochondria surface area and number of cristae were calculated using NIH Image J software.

Measurement of enzymatic activities of Electron Transport Chain complexes

The enzyme activities of the ETC complex were measured in the heart tissue lysates of WT and miR-574 KO mice after Sham or TAC surgeries using a spectrophotometer (Beckman, DU 640B). Activities of complex I (Rotenone sensitive), complex II (Malonate sensitive), complex III (Antimycin A sensitive), and complex IV (potassium cyanide sensitive) were determined using isolated cardiac mitochondria according to the methods as previously described (Choksi *et al*, 2007; Spinazzi *et al*, 2012). Cardiac mitochondria were isolated from WT and miR-574 KO mouse hearts. The heart tissues were placed in 1 mL of ice-cold mitochondria isolation buffer (D-Mannitol 225 mM, Sucrose 75 mM, K_2HPO_4 2 mM, EDTA 1 mM (pH 7.4)) and homogenized using Bertin tissue homogenizer (Bertin Technologies). The tissue homogenate was centrifuged at 1,300 g for 5 mins at 4°C. The supernatant was then layered on 15% percoll and centrifuged at 34,000 g for 10 mins. After centrifugation, the tissue homogenate was re-suspended in the mitochondria isolation buffer and incubated with 0.02% digitonin and centrifuged at 8,000 g for 10 mins. The pellet was shed twice in 1.5 mL of mitochondria isolation buffer and centrifuged again at 8,000 g for 10 mins. The final pellet was suspended in the isolation buffer to be used as cardiac mitochondria. A total protein concentration was determined using Bradford protein assay (Bio-Rad Laboratory).

The complex I activity was measured at 340 nm in 1 mL of reaction mixture containing (50 mM potassium phosphate (pH 7.4), 5 mM $MgCl_2$, 1 mg/mL of BSA followed by 10 μg of total mitochondria proteins isolated from heart tissues as described above, 2 mM KCN, 3.7 μM Antimycin A and 100 μM co-enzyme Q1. The reaction was started by the addition of 140 μM NADH and after 3 mins 20 μM Rotenone was added to the reaction to inhibit the enzyme activity. The enzyme activity was calculated by subtracting the Rotenone insensitive rate from an initial rate. The complex II enzymatic activity was measured at 600 nm in 1 mL of reaction mixture before the reaction started. Mixture was incubated at

37°C for 10 mins containing the reaction buffer (50 mM potassium phosphate (pH 7.4), 5 mM $MgCl_2$, 1 mg/mL BSA, 10 μg of total mitochondria proteins, 20 mM succinate, and 0.2 mM ATP. The reaction was started by the addition of 20 μM Rotenone, 2 mM KCN, 3.7 μM Antimycin A, 50 μM 2,6-Dichlorophenolindophenol (DCPIP), and 100 μM co-enzyme Q1. After 3 mins of reaction time, 10 mM Malonate was added to stop the reaction. The enzyme activity was measured by subtracting the Malonate insensitive rate from an initial rate. The complex III activity was measured at 500 nm in 1 mL of reaction mixture containing the reaction buffer (50 mM potassium phosphate (pH 7.4), 5 mM $MgCl_2$, 1 mg/mL BSA, 10 μg of total mitochondria proteins, 20 μM Rotenone, 2 mM KCN, 0.2 mM ATP, and 40 μM cytochrome C. The reaction was started by the addition of 100 μM decyl benzoquinone with or without 7.4 μM Antimycin A. The enzyme activity was measured by subtracting the Antimycin A insensitive rate from the rate before adding the inhibitor. The complex IV activity was measured at 550 nm in 1 mL of reaction buffer containing (10 mM Tris-HCl (pH 7.4), 20 mM KCl and 1 mg/mL BSA), 5 μg of total mitochondria proteins, and 1 mM dodecyl-β-D-maltoside. The reaction was initiated by the addition of 11 μM of ferrocytochrome C with or without KCN. The enzyme activity was measured by subtracting KCN insensitive rate from the rate before adding the inhibitor.

Formula for ETC enzyme activity calculation (moles of substrate converted per unit time):

$$\text{Enzyme activity} = \frac{\Delta\ \text{Absorbance/min} \times 1000}{\text{Extinction coefficient } (\varepsilon) \times \text{Volume of sample} \times \text{protein concentration}}$$

Complex-I: $\varepsilon = 6.2\ mM^{-1}\ cm^{-1}$; Complex-II: $\varepsilon = 19.1\ mM^{-1}\ cm^{-1}$; Complex-III: $\varepsilon = 18.5\ mM^{-1}cm^{-1}$; Complex-IV: $\varepsilon = 18.5\ mM^{-1}\ cm^{-1}$.

Determination of mitochondrial DNA copy number

AC16 cells were used to determine the effect of miR-574 overexpression on mitochondrial copy number with or without FAM210A overexpression. AC16 cells stably expressing control empty or FAM210A-overexprssing plasmid were transfected with 100 nM negative control (NC) miRNA, miR-574-5p or miR-574-3p mimics, respectively. The cells were lysed in Trizol reagent 48 hrs after transfection and DNA was extracted following the manual. Finally, 5 ng of total DNA containing nuclear and mitochondrial genomic DNA was loaded in each quantitative PCR reaction. 12S rRNA was used to determine mitochondrial DNA copy number and a primer set targeting human chromosome 15 was used to determine nuclear DNA copy number as a normalizer.

Mitochondrial copy number was also detected in the hearts of WT and miR-574 KO mice after 3 days post Sham or TAC surgery. The heart tissues were lysed in Trizol reagent and DNA was isolated according to the manual instructions. 5 ng of total DNA was used for qPCR. 12S rRNA was used to determine the mitochondrial DNA copy number and a primer set that targets the mouse chromosome 11 was used to determine the nuclear genomic DNA copy number and served as the loading control.

Measurement of creatine kinase (CK) activity

The creatine kinase activity was measured using an ELISA based spectrophotometric Creatine Kinase Assay kit according to the manufacturer's instructions (Abcam, Cat. No. ab155901). Isolated tissues were homogenized in the assay buffer, centrifuged at 10,000

rpm for 5 mins, and then the supernatant was used for the assay. Protein concentrations were determined by the Bradford assay. Diluted supernatants (150-200 ng) were mixed with ATP, creatine kinase substrate, CK enzyme mix, and developed in a 96-well ELISA plate. The absorbance was read at 450 nm for 10 mins using the microplate reader. NADH was used as a standard. The CK activity was represented as U/mg/min.

Measurement of alanine aminotransferase (ALT) activity

The alanine aminotransaminase activity was measured using the ALT assay kit (Cayman chemicals, Cat. No. 700260), based on a colorimetric method. Isolated tissues were homogenized in 100 mM Tris-HCl buffer (pH 7.8) and centrifuged at 10,000 g for 15 mins. The ALT substrate and cofactors were added to the diluted supernatant in the 96-well ELISA plate and incubated at 37 °C for 15 mins. Then the reaction was started by adding the ALT initiator and measured at 340 nm. The CK activity was represented as U/L.

Statistical Analysis

All quantitative data were presented as mean ± SEM, and analyzed using Prism 8.3.0 software (GraphPad). We used a Kolmogorov-Smirnov test to assess if the data was normally distributed. For a comparison between 2 groups, an unpaired Mann-Whitney test for not normally distributed data and an unpaired two-tailed Student t-test for normally distributed data were performed. For multiple comparisons among ≥3 groups, one-way or two-way ANOVA with Tukey's method for post hoc comparisons and non-parametric Kruskal-Wallis test with Dunn's multiple comparisons were performed. Two-sided *P* values <0.05 were considered to indicate statistical significance. Specific statistical methods and post hoc tests were described in the figure legends.

Bioinformatic analysis for RNA-sequencing

Software summary:

DATA FORMATTING (Illumina -> FastQ) & DEMULTIPLEXING - bcltofastq-1.8.4

CLEANING - Trimmomatic-0.32 "SLIDINGWINDOW:4:20 TRAILING:13 LEADING:13 ILLUMINACLIP:adapters.fasta:2:30:10 MINLEN:15"

MAPPING - STAR_2.4.2a "--twopassMode Basic --runMode alignReads --genomeDir $(Bartel) --readFilesIn $(Aguayo *et al*) --outSAMtype BAM SortedByCoordinate --outSAMstrandField intronMotif --outFilterIntronMotifs RemoveNoncanonical"

REFERENCE - GRCm38.p4 + Gencode-M6 Annotation

READ COUNTING - htseq-count v0.6.1 "-q -f bam -s no -r pos -i gene_name"

DIFFERENTIAL EXP. - DESeq2_1.12.4

R version 3.3.0 (2016-05-03)

Platform: x86_64-pc-linux-gnu (64-bit)

Running under: Red Hat Enterprise Linux Server release 6.6 (Santiago)

locale:

```
 [1] LC_CTYPE=en_US.UTF-8       LC_NUMERIC=C
 [3] LC_TIME=en_US.UTF-8        LC_COLLATE=en_US.UTF-8
 [5] LC_MONETARY=en_US.UTF-8    LC_MESSAGES=en_US.UTF-8
 [7] LC_PAPER=en_US.UTF-8       LC_NAME=C
 [9] LC_ADDRESS=C               LC_TELEPHONE=C
[11] LC_MEASUREMENT=en_US.UTF-8 LC_IDENTIFICATION=C

attached base packages:
[1] parallel  stats4    stats     graphics  grDevices utils     datasets
[8] methods   base
other attached packages:
 [1] calibrate_1.7.2            MASS_7.3-45
 [3] genefilter_1.54.2          RColorBrewer_1.1-2
 [5] pheatmap_1.0.8             BiocParallel_1.6.6
 [7] DESeq2_1.12.4              SummarizedExperiment_1.2.3
 [9] Biobase_2.32.0             GenomicRanges_1.24.3
[11] GenomeInfoDb_1.8.7         IRanges_2.6.1
[13] S4Vectors_0.10.3           BiocGenerics_0.18.0
loaded via a namespace (and not attached):
 [1] Rcpp_0.12.7          plyr_1.8.4           XVector_0.12.1
 [4] zlibbioc_1.18.0      rpart_4.1-10         annotate_1.50.0
 [7] RSQLite_1.0.0        gtable_0.2.0         lattice_0.20-34
[10] Matrix_1.2-7.1       DBI_0.5-1            gridExtra_2.2.1
[13] cluster_2.0.4        locfit_1.5-9.1       grid_3.3.0
[16] nnet_7.3-12          data.table_1.9.6     AnnotationDbi_1.34.4
[19] XML_3.98-1.4         survival_2.39-5      foreign_0.8-67
[22] latticeExtra_0.6-28  Formula_1.2-1        geneplotter_1.50.0
[25] ggplot2_2.1.0        Hmisc_3.17-4         scales_0.4.0
[28] splines_3.3.0        colorspace_1.2-6     xtable_1.8-2
```

a. [31] acepack_1.3-3.3 munsell_0.4.3 chron_2.3-47

Target antigen	Vendor or Source	Catalog #	Working concentration	Persistent ID / URL of antibodies
FAM210A	Sigma-Aldrich	HPA014324	IB: 1:1000<br>IF: 1:300	https://www.sigmaaldrich.com/catalog/product/sigma/hpa014324?lang=en&region=US
VDAC	Santa Cruz	sc-390996	IB: 1:500	https://www.scbt.com/p/vdac1-antibody-b-6?requestFrom=search
ATP5B	Santa Cruz	sc-74549	IB: 1:500<br>IF: 1:200	https://www.scbt.com/p/atp5b-antibody-a-8?requestFrom=search
TOM20	Santa Cruz	sc-17764	IB: 1:500	https://www.scbt.com/p/tom20-antibody-f-10?requestFrom=search
EF-Tu	Santa Cruz	sc-393924	IB: 1:500<br>IF: 1:100	https://www.scbt.com/p/ef-tu-antibody-a-5?requestFrom=search
NDUFS1	Santa Cruz	sc-271510	IB: 1:500<br>IF: 1:100	https://www.scbt.com/p/ndufs1-antibody-e-8?requestFrom=search
COX2	Santa Cruz	sc-514489	IB: 1:500<br>IF: 1:200	https://www.scbt.com/p/cox2-antibody-d-5?requestFrom=search
UQCRC2	Santa Cruz	sc-390161	IB: 1:500<br>IF:1:200	https://www.scbt.com/p/uqcrc2-antibody-g-4?requestFrom=search
COX4	ProteinTech	66110-1-Ig	IB: 1:1000<br>IF: 1:300	https://www.ptglab.com/products/COXIV-Antibody-66110-1-Ig.htm
SDHA	ProteinTech	66588-1-Ig	IB: 1:1000<br>IF: 1:300	https://www.ptglab.com/products/SDHA-Antibody-66588-1-Ig.htm
CYTB	ThermoFisher Scientific	PA5-43533	IB: 1:1000<br>IF: 1:300	https://www.thermofisher.com/antibody/product/MT-CYB-Antibody-Polyclonal/PA5-43533
ATP8	ThermoFisher Scientific	PA5-68103	IB: 1:1000<br>IF: 1:300	https://www.thermofisher.com/antibody/product/MT-ATP8-Antibody-Polyclonal/PA5-68103
ATP6	ThermoFisher Scientific	PA5-37129	IB: 1:1000	https://www.thermofisher.com/antibody/product/MT-ATP6-Antibody-Polyclonal/PA5-37129
ND1	ProteinTech	19703-1-AP	IB: 1:1000<br>IF: 1:200	https://www.ptglab.com/products/ND1-Antibody-19703-1-AP.htm
FLAG	Sigma-Aldrich	F1804	IF: 1:250<br>IB: 1:2000	https://www.sigmaaldrich.com/catalog/product/sigma/f1804?lang=en&region=US
FLAG	ProteinTech	20543-1-AP	IF: 1:250<br>IB: 1:2000	https://www.ptglab.com/products/Flag-Tag-Antibody-20543-1-AP.htm
α-Tubulin	ProteinTech	11224-1-AP	IB: 1:2000	https://www.ptglab.com/products/TUBA1B-Antibody-11224-1-AP.htm
GAPDH	ProteinTech	60004-1-Ig	IB: 1:5000	https://www.ptglab.com/products/GAPDH-Antibody-60004-1-Ig.htm
Alexa Flour ™ 488 Phalloidin	ThermoFisher Scientific	A12379	IF:1:1000	https://www.thermofisher.com/order/catalog/product/A12379#/A12379

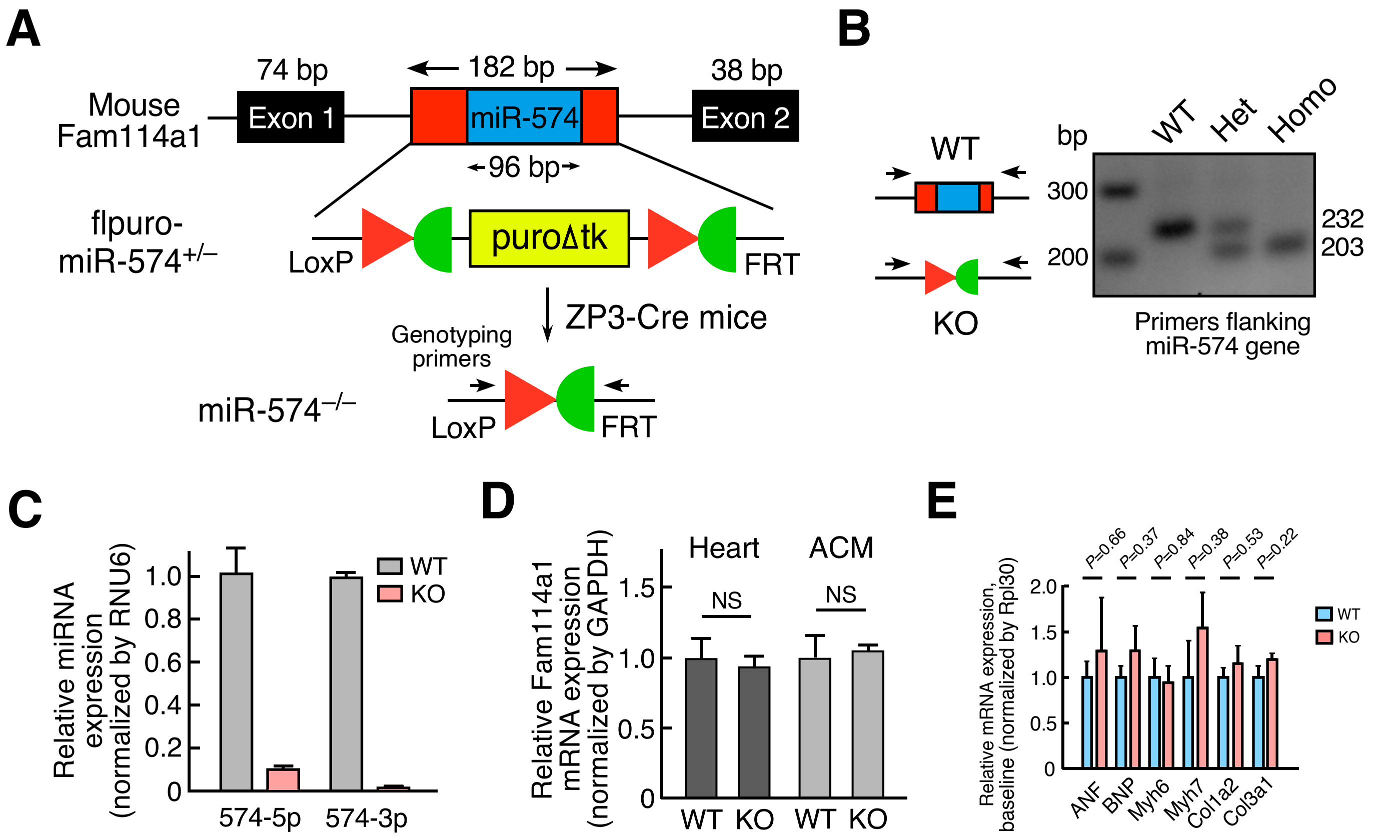

Appendix Figure S1. Generation of miR-574 global knockout mice and phenotypic characterization. (**A**) The schematic of the generation of miR-574$^{-/-}$ mice. (**B**) Genotyping of miR-574$^{-/-}$ mice with removed puromycin resistance cassette. (**C**) miR-574-5p/3p expression in WT and miR-574$^{-/-}$ mice. N=3. (**D**) The expression of host gene *Fam114a1* mRNA in the heart and CMs of WT and miR-574$^{-/-}$ mice. N=3. NS: not significant. (**E**) RT-qPCR of fetal cardiac genes in WT and miR-574$^{-/-}$ mice at baseline. Data were presented as mean ± SEM. N=3. P values were calculated by unpaired two-tailed Student *t* test (D, E).

A

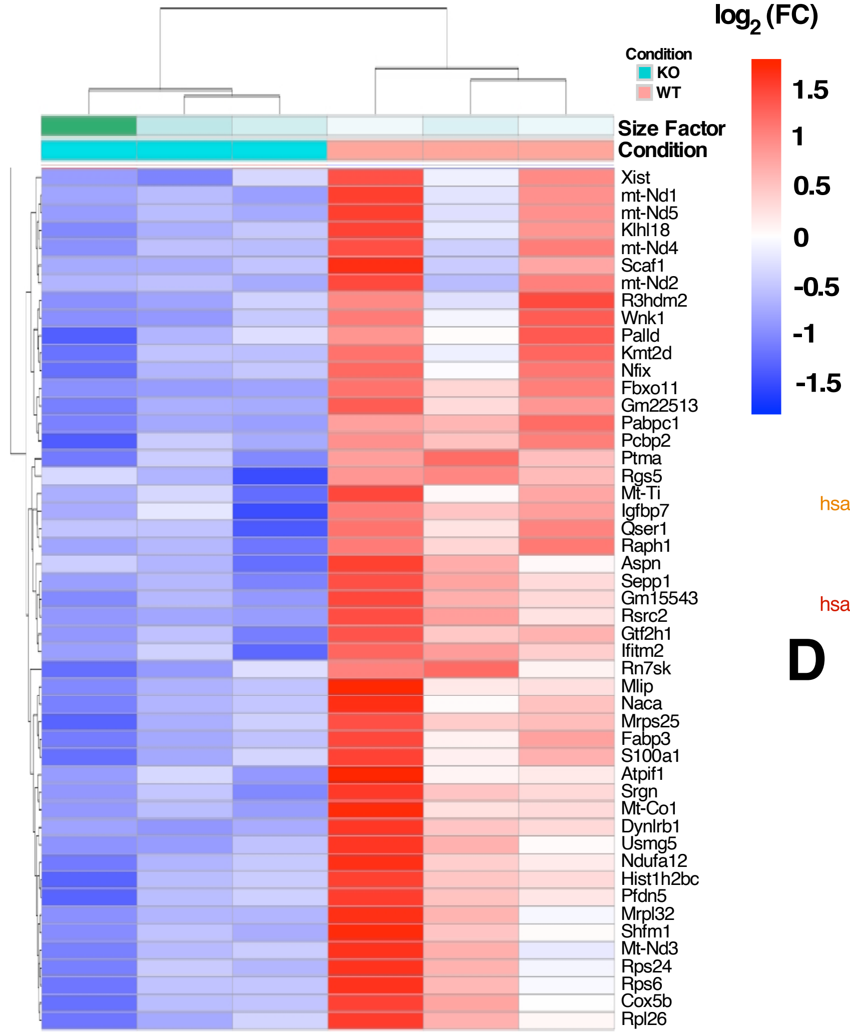

B

Downregulated genes

Gene enriched pathway	Gene NO.	FDR q-value
Respiratory electron transport complex	7	4.90 e-9
ATP synthesis & TCA cycle	7	1.02 e-7
Oxidative phosphorylation	6	2.53 e-6

C

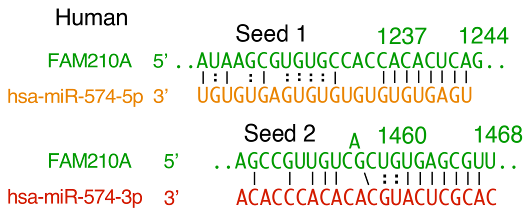

D

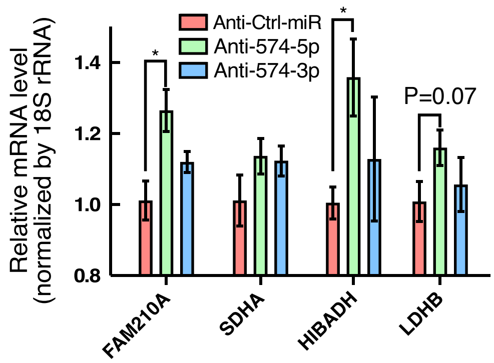

Appendix Figure S2. Downregulated genes in miR-574 KO versus WT mouse heart. (**A**) Heatmap of significantly downregulated genes in miR-574$^{-/-}$ mice at baseline analyzed by RNA-Seq. P60 male mice, n=3 per group, $p_{adj}<0.05$. (**B**) Gene Ontology analysis of enriched pathways of downregulated genes in RNA-Seq. The top three pathways are listed with enriched gene sets. (**C**) Seed sequence sites of miR-574-5p and miR-574-3p in human FAM210A mRNA. (**D**) Validation of miR-574-5p target genes using RT-qPCR analysis of mRNA expression in AC16 cells transfected with control anti-miR or anti-miR-574-5p/3p inhibitors. N=6. The statistical calculation is performed in comparison with the control anti-miR transfection sample. Data were presented as mean ± SEM. *P* values were calculated by Student *t* test (D).

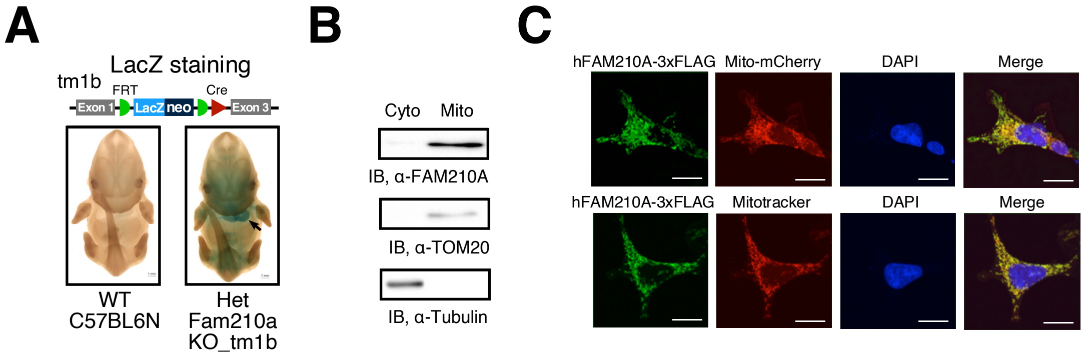

Appendix Figure S3. Organ-specific expression and cellular localization of FAM210A. (**A**) Expression of LacZ reporter in E12.5 murine embryo of heterozygous global Fam210a KO mice. Heart-specific high expression of LacZ was observed in all of 9 heterozygous KO mice (IMPC). Tm1b allele: LacZ tagged null allele. (**B**) Cellular fractionation and immunoblot for FAM210A protein in AC16 cells. TOM20 and α-Tubulin are marker proteins for mitochondria and cytoplasm, respectively. A representative image is shown in replicated experiments. (**C**) Cellular localization of recombinant FAM210A in HEK293T cells. FAM210A-3xFLAG expression plasmid was transfected into HEK293T cells for 48 hrs. Anti-FLAG antibody was used for IF. Mito-mCherry or mitotracker were used to label mitochondria. Scale bar: 10 μm.

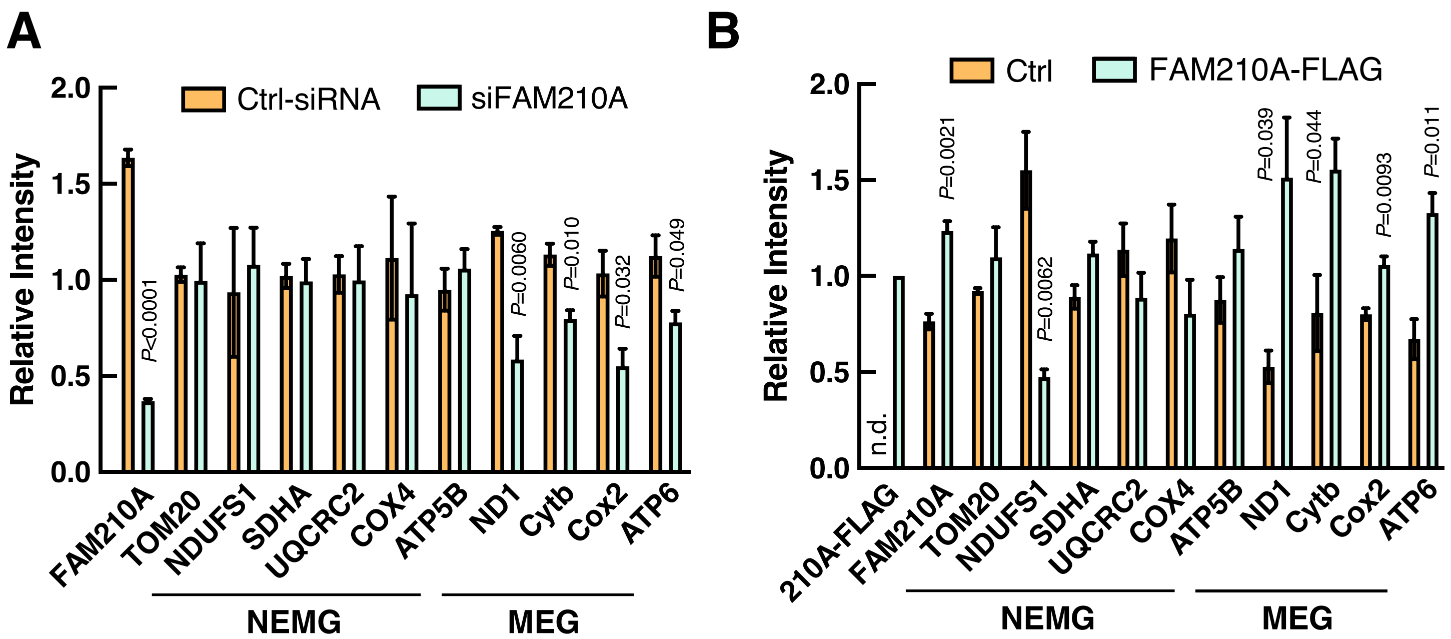

Appendix Figure S4. (**A**) and (**B**) Quantitative analysis of Western blot data from Figure 7F, G. n.d., not detected. Each experiment was done in triplicates. Data were presented as mean ± SEM. *P* values were calculated by unpaired two-tailed Student *t* test.

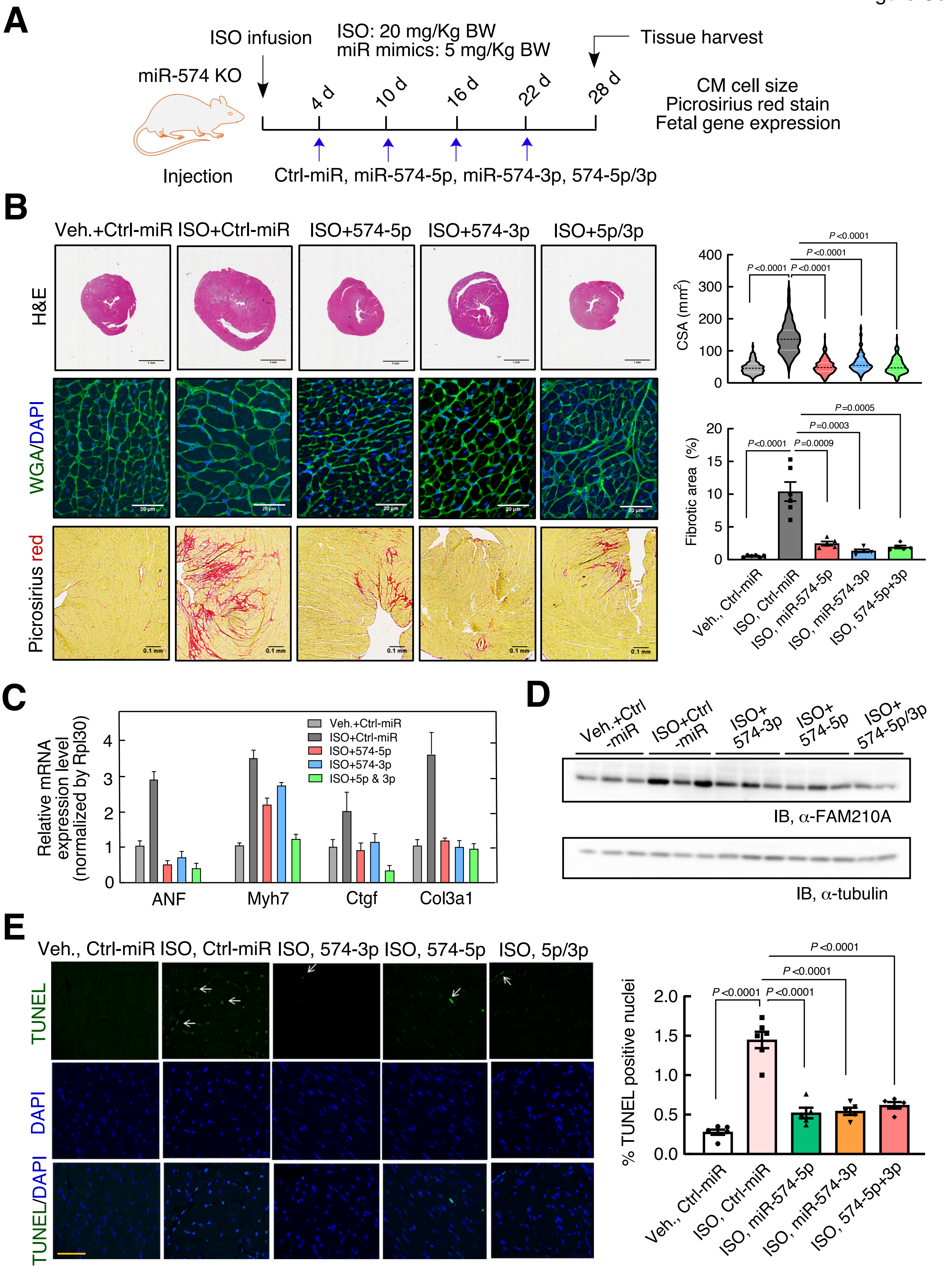
A
ISO infusion
ISO: 20 mg/Kg BW
miR mimics: 5 mg/Kg BW
Tissue harvest
miR-574 KO
4 d
10 d
16 d
22 d
28 d
CM cell size
Picrosirius red stain
Fetal gene expression
Injection
Ctrl-miR, miR-574-5p, miR-574-3p, 574-5p/3p
B
Veh.+Ctrl-miR
ISO+Ctrl-miR
ISO+574-5p
ISO+574-3p
ISO+5p/3p
H&E
WGA/DAPI
Picrosirius red
CSA (mm2)
P<0.0001
Fibrotic area (%)
P=0.0005
P=0.0003
P=0.0009
Veh., Ctrl-miR
ISO, Ctrl-miR
ISO, miR-574-5p
ISO, miR-574-3p
ISO, 574-5p+3p
C
Relative mRNA expression level (normalized by Rpl30)
Veh.+Ctrl-miR
ISO+Ctrl-miR
ISO+574-5p
ISO+574-3p
ISO+5p & 3p
ANF
Myh7
Ctgf
Col3a1
D
Veh.+Ctrl -miR
ISO+Ctrl -miR
ISO+ 574-3p
ISO+ 574-5p
ISO+ 574-5p/3p
IB, α-FAM210A
IB, α-tubulin
E
Veh., Ctrl-miR
ISO, Ctrl-miR
ISO, 574-3p
ISO, 574-5p
ISO, 5p/3p
TUNEL
DAPI
TUNEL/DAPI
% TUNEL positive nuclei
P<0.0001
Veh., Ctrl-miR
ISO, Ctrl-miR
ISO, miR-574-5p
ISO, miR-574-3p
ISO, 574-5p+3p

Appendix Figure S5. miR-574-5p/3p injection reduces the cardiac pathological remodelling in ISO-treated miR-574 null mice. (**A**) The schematic of the ISO infusion in miR-574$^{-/-}$ mice using miRNA mimics followed by phenotypic characterizations. NC, negative control. (**B**) H&E (scale bar: 1 mm), WGA staining (scale bar: 20 um; n=200-250 CMs), and picrosirius red staining (scale bar: 0.1 mm) of murine hearts in the rescue models. n=5-6 mice per group. (**C**) RT-qPCR of hypertrophy and fibrosis marker genes in the hearts of miR-574$^{-/-}$ mice with ISO treatment. N=4 mice/group. (**D**) Western blotting of FAM210A in the hearts of rescue mouse models. (**E**) TUNEL assay of murine hearts in the rescue models. Scale bar: 30 um. N=5-6 mice/group. Data were presented as mean ± SEM. P values were calculated by Non-parametric two tailed Mann Whitney test (WGA staining), unpaired two-tailed Student t test (Picrosirius red and TUNEL staining).

Appendix Table S1. 34 upregulated and 49 downregulated genes in miR-574 KO vs. WT hearts

FC>1.25 or <0.8, padj<0.05

	base Mean	Fold Change	log2 Fold Change	lfcSE	stat	pvalue	padj
Etfb	408097.1982	1.673691228	0.743033396	0.131863196	5.634880814	1.75E-08	2.16E-05
Tmem59	11731.83352	1.567118047	0.648113858	0.163491787	3.964198265	7.36E-05	0.009388216
Afg3l2	43112.10403	1.566069624	0.647148353	0.151607351	4.268581614	1.97E-05	0.006060611
Psmc5	7324.357243	1.559143586	0.640753796	0.167778157	3.819053739	0.000133965	0.014566678
Psmd3	7320.889973	1.539625167	0.622579159	0.166571813	3.737602108	0.000185784	0.019078949
Atp5g3	5605.518323	1.526409247	0.610141816	0.171296245	3.561910049	0.000368167	0.031103114
Bcat2	3943.561087	1.491658328	0.576917117	0.174562254	3.304936221	0.00094998	0.046887226
Htra1	36387.89581	1.487301986	0.572697606	0.136641007	4.191257214	2.77E-05	0.007245358
Tspyl1	10300.1618	1.44649547	0.532561805	0.134251702	3.966890525	7.28E-05	0.009388216
Unc45b	95697.92432	1.423694471	0.509639573	0.110778387	4.600532539	4.21E-06	0.00223323
Akr1b3	38758.30326	1.422762279	0.508694631	0.119875702	4.243517427	2.20E-05	0.006257692
Psap	3523.189032	1.421601178	0.507516782	0.155718662	3.259190495	0.001117306	0.049909774
Rpl3l	5043.463944	1.419956731	0.505846969	0.143722341	3.519612648	0.000432177	0.032607349
Etfa	3899.821924	1.414437221	0.500228145	0.151449409	3.302938895	0.000956772	0.046887226
Hspa12a	4420.826456	1.411729372	0.497463551	0.145780291	3.412419799	0.000643889	0.039674276
Gnpat	22504.31057	1.404701208	0.490263289	0.120543094	4.067120487	4.76E-05	0.008379451
Dazap2	15223.56913	1.396923518	0.482253035	0.119911834	4.02173011	5.78E-05	0.008928648
Ldhb	99706.91872	1.392624504	0.477806314	0.103874887	4.599825137	4.23E-06	0.00223323
Cpe	16182.68515	1.388892553	0.473934994	0.117060707	4.048625765	5.15E-05	0.008657575
Dgat2	11888.17609	1.372273991	0.456568562	0.115134449	3.965525241	7.32E-05	0.009388216
Susd6	6390.752474	1.370705571	0.454918712	0.125604977	3.621820745	0.000292537	0.027730989
Aqp1	33778.80465	1.352598942	0.43573413	0.105597548	4.126365979	3.69E-05	0.008217666
Sdha	17920.4262	1.35110067	0.434135173	0.106465952	4.077690235	4.55E-05	0.008379451
Ivd	99922.39785	1.350049285	0.433012075	0.093741618	4.619208444	3.85E-06	0.00223323
Pfkm	202581.0027	1.337062629	0.419067044	0.081043742	5.17087481	2.33E-07	0.000215351
Actr1a	4118.106403	1.329713099	0.411115001	0.124915083	3.291155802	0.000997766	0.046887226
Atp1a2	45436.551	1.32873395	0.410052265	0.095168776	4.308684881	1.64E-05	0.005519559
Lamb2	3774.693454	1.327018216	0.408188175	0.123054009	3.317146486	0.000909419	0.046887226
Arf1	5177.915777	1.312976717	0.392841333	0.110996063	3.539236645	0.000401286	0.032251177
Vcp	5068.806828	1.304921108	0.383962588	0.108894875	3.525993203	0.000421898	0.03249492
Slc25a3	59542.69283	1.271758857	0.346825142	0.0771586	4.494964176	6.96E-06	0.00285826
Hibadh	4883.496771	1.271149643	0.346133878	0.099726775	3.470821928	0.000518868	0.036001036
Got1	3584.018639	1.267605925	0.342106308	0.104845446	3.262958211	0.001102558	0.049909774
Fam210a	3784.178761	1.253394845	0.325840965	0.09904018	3.289987593	0.001001918	0.046887226
Sepp1	4783.591678	0.803027138	-0.316479351	0.091384152	-3.463175466	0.00053384	0.036001036
Rgs5	4298.750953	0.795467044	-0.330125934	0.097361388	-3.390727482	0.000697074	0.04090606
Ptma	3651.52794	0.787335899	-0.344948835	0.104602893	-3.297698808	0.000974806	0.046887226
Cox5b	3937.243267	0.740911762	-0.432626359	0.131029385	-3.301750661	0.000960834	0.046887226
Gm15543	4267.733975	0.734277944	-0.445601829	0.13285328	-3.354089768	0.000796265	0.045289118
Igfbp7	4327.136784	0.730014056	-0.454003852	0.133696186	-3.395787619	0.000684314	0.040805002
Ifitm2	3602.310337	0.72217354	-0.469582534	0.143049747	-3.282651966	0.001028355	0.047522874
Rsrc2	7119.896748	0.71726765	-0.479416531	0.128115264	-3.74207192	0.000182509	0.019078949
Wnk1	6572.658329	0.708668549	-0.496817071	0.136676467	-3.634986213	0.000277996	0.027423325
Rps24	3635.108291	0.706299117	-0.501648803	0.152431936	-3.290969187	0.000998428	0.046887226
Qser1	4830.372136	0.703455591	-0.507468746	0.146685257	-3.459575676	0.000541027	0.036001036
Raph1	7729.475802	0.701265926	-0.511966465	0.132015282	-3.87808486	0.000105282	0.012555727
Gtf2h1	4270.291531	0.694987789	-0.524940466	0.155633443	-3.372928441	0.000743733	0.042962189
Mlip	3770.114831	0.693889934	-0.527221257	0.160018463	-3.294752673	0.000985083	0.046887226
Rps6	3609.874701	0.688330871	-0.538825879	0.163697238	-3.291600301	0.000996191	0.046887226
Rpl26	5780.169463	0.687701489	-0.540145626	0.150788166	-3.582148661	0.00034078	0.029996733
Xist	4667.5674	0.68738803	-0.540803366	0.157745209	-3.428334655	0.000607296	0.038053806
Hist1h2bc	4398.137985	0.68393169	-0.548075857	0.161206113	-3.399845362	0.00067424	0.040805002
Pfdn5	4879.341557	0.681814531	-0.552548749	0.159814164	-3.457445419	0.000545323	0.036001036
Palld	3696.238678	0.680980478	-0.554314655	0.16821062	-3.295360639	0.000982954	0.046887226
Mrpl32	3718.909584	0.680794897	-0.554707872	0.167560659	-3.310489922	0.000931328	0.046887226
Srgn	4742.961963	0.676981458	-0.562811774	0.163339534	-3.445655568	0.000569676	0.036948983
Fabp3	38484.13233	0.670568288	-0.576543838	0.137992246	-4.17808865	2.94E-05	0.007245358

Ndufa12	7091.741933	0.669944508	-0.577886494	0.159135429	-3.631413166	0.000281874	0.027423325
Shfm1	5125.078175	0.668533439	-0.580928371	0.164468908	-3.532147064	0.0004122	0.03242348
Mrps25	5463.563305	0.667887991	-0.58232192	0.16355144	-3.560481768	0.000370175	0.031103114
Fbxo11	30566.47047	0.667838322	-0.582429215	0.141345475	-4.120607435	3.78E-05	0.008217666
Pcbp2	20905.02604	0.665711315	-0.587031406	0.143585763	-4.088367782	4.34E-05	0.008379451
mt-Ti	4461.158506	0.660150118	-0.599133964	0.174598846	-3.431488691	0.000600278	0.038053806
mt-Co1	7655.509742	0.658434236	-0.602888742	0.156010615	-3.864408481	0.000111359	0.012865414
R3hdm2	3635.169161	0.651105628	-0.619036486	0.186667581	-3.316250643	0.00091234	0.046887226
Aspn	5347.737722	0.650041603	-0.62139604	0.174929279	-3.552270053	0.000381923	0.031377069
mt-Nd3	4195.03296	0.646728151	-0.628768684	0.188143649	-3.341960724	0.000831888	0.04659835
Klhl18	3564.307969	0.642401267	-0.638453357	0.195942013	-3.258379087	0.001120506	0.049909774
S100a1	14263.13091	0.634414726	-0.656501836	0.163270106	-4.020955528	5.80E-05	0.008928648
Usmg5	17293.69612	0.633847026	-0.657793395	0.161328903	-4.077343744	4.56E-05	0.008379451
Dynlrb1	58877.11683	0.630946656	-0.664410058	0.150251638	-4.421982131	9.78E-06	0.003615649
mt-Nd1	9812.006214	0.619464192	-0.690907205	0.177325134	-3.896273406	9.77E-05	0.012037939
mt-Nd4	5822.489045	0.617240614	-0.696095101	0.193078857	-3.605237329	0.000311868	0.028121347
Atpif1	4904.522188	0.613382932	-0.705140072	0.203882162	-3.458566774	0.000543058	0.036001036
Naca	7356.185561	0.612454834	-0.707324639	0.18380298	-3.84827623	0.000118952	0.013326215
Nfix	14158.01338	0.610954291	-0.710863646	0.17733106	-4.008680977	6.11E-05	0.009029381
mt-Nd2	4743.910206	0.608407484	-0.716890195	0.206721245	-3.467907683	0.000524527	0.036001036
Rn7sk	90390.71577	0.589228353	-0.763101241	0.169052446	-4.513991134	6.36E-06	0.00285826
mt-Nd5	4917.865453	0.587345229	-0.767719358	0.219314018	-3.500548507	0.000464302	0.03433047
Kmt2d	12805.42761	0.57889339	-0.788630412	0.197618606	-3.990668829	6.59E-05	0.009368657
Scaf1	6383.038706	0.576686482	-0.794140891	0.219737724	-3.614039845	0.000301463	0.02786269
Pabpc1	823138.3637	0.554150678	-0.851649784	0.130341579	-6.533983937	6.40E-11	1.18E-07
Gm22513	5103305.093	0.372954078	-1.422930094	0.202500498	-7.026798004	2.11E-12	7.81E-09

Appendix Table S2. FAM210A interactome capture by IP-mass spectrometry

			SEQUEST Score		# of Peptides		# of PSMs	
Accession	Description	MW [kDa]	Fam210a	IgG	Fam210a	IgG	Fam210a	IgG
P13533	Myosin-6 OS=Homo sapiens GN=MYH6 PE=1 SV=5 - [MYH6_HUMAN]	223.6	135.23		51		59	
P12883	Myosin-7 OS=Homo sapiens GN=MYH7 PE=1 SV=5 - [MYH7_HUMAN]	223.0	117.61		42		50	
P48735	Isocitrate dehydrogenase [NADP], mitochondrial OS=Homo sapiens GN=IDH2 PE=1 SV=2 - [IDHP_HUMAN]	50.9	86.55	6.75	22	2	35	4
P25705	ATP synthase subunit alpha, mitochondrial OS=Homo sapiens GN=ATP5A1 PE=1 SV=1 - [ATPA_HUMAN]	59.7	69.14	7.22	20	3	28	3
A3KMH1	von Willebrand factor A domain-containing protein 8 OS=Homo sapiens GN=VWA8 PE=1 SV=2 - [VWA8_HUMAN]	214.7	58.47		21		26	
P16615	Sarcoplasmic/endoplasmic reticulum calcium ATPase 2 OS=Homo sapiens GN=ATP2A2 PE=1 SV=1 - [AT2A2_HUMAN]	114.7	41.80		19		21	
Q9Y623	Myosin-4 OS=Homo sapiens GN=MYH4 PE=2 SV=2 - [MYH4_HUMAN]	222.9	39.59		17		18	
Q16891	MICOS complex subunit MIC60 OS=Homo sapiens GN=IMMT PE=1 SV=1 - [MIC60_HUMAN]	83.6	37.28	4.08	12	3	14	3
P50213	Isocitrate dehydrogenase [NAD] subunit alpha, mitochondrial OS=Homo sapiens GN=IDH3A PE=1 SV=1 - [IDH3A_HUMAN]	39.6	35.31		11		13	
P63267	Actin, gamma-enteric smooth muscle OS=Homo sapiens GN=ACTG2 PE=1 SV=1 - [ACTH_HUMAN]	41.8	34.41	1.63	11	1	15	1
P10809	60 kDa heat shock protein, mitochondrial OS=Homo sapiens GN=HSPD1 PE=1 SV=2 - [CH60_HUMAN]	61.0	32.77		14		15	
P60709	Actin, cytoplasmic 1 OS=Homo sapiens GN=ACTB PE=1 SV=1 - [ACTB_HUMAN]	41.7	32.75	3.47	10	2	14	2
P06576	ATP synthase subunit beta, mitochondrial OS=Homo sapiens GN=ATP5B PE=1 SV=3 - [ATPB_HUMAN]	56.5	32.65	2.27	12	1	15	1
P49411	**Elongation factor Tu, mitochondrial OS=Homo sapiens GN=TUFM PE=1 SV=2 - [EFTU_HUMAN]**	49.5	32.04		12		14	
P08238	Heat shock protein HSP 90-beta OS=Homo sapiens GN=HSP90AB1 PE=1 SV=4 - [HS90B_HUMAN]	83.2	27.00		11		12	
Q02218	2-oxoglutarate dehydrogenase, mitochondrial OS=Homo sapiens GN=OGDH PE=1 SV=3 - [ODO1_HUMAN]	115.9	25.81	1.71	11	1	12	1
Q96ND0	Protein FAM210A OS=Homo sapiens GN=FAM210A PE=1 SV=2 - [F210A_HUMAN]	30.8	24.66		6		13	
Q02978	Mitochondrial 2-oxoglutarate/malate carrier protein OS=Homo sapiens GN=SLC25A11 PE=1 SV=3 - [M2OM_HUMAN]	34.0	23.88		7		10	
Q9UJS0	Calcium-binding mitochondrial carrier protein Aralar2 OS=Homo sapiens GN=SLC25A13 PE=1 SV=2 - [CMC2_HUMAN]	74.1	23.49		9		11	
P38646	Stress-70 protein, mitochondrial OS=Homo sapiens GN=HSPA9 PE=1 SV=2 - [GRP75_HUMAN]	73.6	23.15		9		9	
O75746	Calcium-binding mitochondrial carrier protein Aralar1 OS=Homo sapiens GN=SLC25A12 PE=1 SV=2 - [CMC1_HUMAN]	74.7	22.93		9		11	
P10515	Dihydrolipoyllysine-residue acetyltransferase component of pyruvate dehydrogenase complex, mitochondrial OS=Homo sapiens GN=DLAT PE=1 SV=3 - [ODP2_HUMAN]	69.0	20.31		8		10	
P35609	Alpha-actinin-2 OS=Homo sapiens GN=ACTN2 PE=1 SV=1 - [ACTN2_HUMAN]	103.8	20.27		10		10	
Q99623	Prohibitin-2 OS=Homo sapiens GN=PHB2 PE=1 SV=2 - [PHB2_HUMAN]	33.3	19.66		10		10	
P05091	Aldehyde dehydrogenase, mitochondrial OS=Homo sapiens GN=ALDH2 PE=1 SV=2 - [ALDH2_HUMAN]	56.3	19.53		8		9	
P05141	ADP/ATP translocase 2 OS=Homo sapiens GN=SLC25A5 PE=1 SV=7 - [ADT2_HUMAN]	32.8	19.16	3.85	11	3	14	3
P11182	Lipoamide acyltransferase component of branched-chain alpha-keto acid dehydrogenase complex, mitochondrial OS=Homo sapiens GN=DBT PE=1 SV=3 - [ODB2_HUMAN]	53.5	19.13		7		11	
P12235	ADP/ATP translocase 1 OS=Homo sapiens GN=SLC25A4 PE=1 SV=4 - [ADT1_HUMAN]	33.0	18.59	7.55	11	5	14	5
Q86YZ3	Hornerin OS=Homo sapiens GN=HRNR PE=1 SV=2 - [HORN_HUMAN]	282.2	18.00	5.48	6	2	6	2
Q99798	Aconitate hydratase, mitochondrial OS=Homo sapiens GN=ACO2 PE=1 SV=2 - [ACON_HUMAN]	85.4	17.74		6		7	
P08559	Pyruvate dehydrogenase E1 component subunit alpha, somatic form, mitochondrial OS=Homo sapiens GN=PDHA1 PE=1 SV=3 - [ODPA_HUMAN]	43.3	17.17		8		11	
P36542	ATP synthase subunit gamma, mitochondrial OS=Homo sapiens GN=ATP5C1 PE=1 SV=1 - [ATPG_HUMAN]	33.0	16.37	0.00	3	1	8	1
Q9NX63	MICOS complex subunit MIC19 OS=Homo sapiens GN=CHCHD3 PE=1 SV=1 - [MIC19_HUMAN]	26.1	16.28	1.94	8	1	9	1
P02768	Serum albumin OS=Homo sapiens GN=ALB PE=1 SV=2 - [ALBU_HUMAN]	69.3	16.12	31.44	5	6	7	16
P13804	Electron transfer flavoprotein subunit alpha, mitochondrial OS=Homo sapiens GN=ETFA PE=1 SV=1 - [ETFA_HUMAN]	35.1	15.95		5		7	
P17661	Desmin OS=Homo sapiens GN=DES PE=1 SV=3 - [DESM_HUMAN]	53.5	15.72		7		7	
P40939	Trifunctional enzyme subunit alpha, mitochondrial OS=Homo sapiens GN=HADHA PE=1 SV=2 - [ECHA_HUMAN]	82.9	14.44	4.18	7	2	10	2
P11142	Heat shock cognate 71 kDa protein OS=Homo sapiens GN=HSPA8 PE=1 SV=1 - [HSP7C_HUMAN]	70.9	13.05	4.06	5	1	5	1
Q9ULD0	2-oxoglutarate dehydrogenase-like, mitochondrial OS=Homo sapiens GN=OGDHL PE=1 SV=3 - [OGDHL_HUMAN]	114.4	12.74	1.71	7	1	8	1
Q9P2R7	Succinate--CoA ligase [ADP-forming] subunit beta, mitochondrial OS=Homo sapiens GN=SUCLA2 PE=1 SV=3 - [SUCB1_HUMAN]	50.3	12.48		6		6	
P11177	Pyruvate dehydrogenase E1 component subunit beta, mitochondrial OS=Homo sapiens GN=PDHB PE=1 SV=3 - [ODPB_HUMAN]	39.2	12.20		5		6	
P12694	2-oxoisovalerate dehydrogenase subunit alpha, mitochondrial OS=Homo sapiens GN=BCKDHA PE=1 SV=2 - [ODBA_HUMAN]	50.4	11.99		5		6	
O00330	Pyruvate dehydrogenase protein X component, mitochondrial OS=Homo sapiens GN=PDHX PE=1 SV=3 - [ODPX_HUMAN]	54.1	11.92		5		5	
P08670	Vimentin OS=Homo sapiens GN=VIM PE=1 SV=4 - [VIME_HUMAN]	53.6	11.85	0.00	8	1	8	1
P42704	Leucine-rich PPR motif-containing protein, mitochondrial OS=Homo sapiens GN=LRPPRC PE=1 SV=3 - [LPPRC_HUMAN]	157.8	11.00		7		9	
P31040	Succinate dehydrogenase [ubiquinone] flavoprotein subunit, mitochondrial OS=Homo sapiens GN=SDHA PE=1 SV=2 - [SDHA_HUMAN]	72.6	10.97		5		5	
Q16836	Hydroxyacyl-coenzyme A dehydrogenase, mitochondrial OS=Homo sapiens GN=HADH PE=1 SV=3 - [HCDH_HUMAN]	34.3	10.87		4		5	
Q15365	Poly(rC)-binding protein 1 OS=Homo sapiens GN=PCBP1 PE=1 SV=2 - [PCBP1_HUMAN]	37.5	10.84		8		9	
Q9Y512	Sorting and assembly machinery component 50 homolog OS=Homo sapiens GN=SAMM50 PE=1 SV=3 - [SAM50_HUMAN]	51.9	10.79		6		6	
P18206	Vinculin OS=Homo sapiens GN=VCL PE=1 SV=4 - [VINC_HUMAN]	123.7	10.48		6		6	
P17540	Creatine kinase S-type, mitochondrial OS=Homo sapiens GN=CKMT2 PE=1 SV=2 - [KCRS_HUMAN]	47.5	10.15		3		4	
Q16851	UTP--glucose-1-phosphate uridylyltransferase OS=Homo sapiens GN=UGP2 PE=1 SV=5 - [UGPA_HUMAN]	56.9	9.94		5		5	
P38117	Electron transfer flavoprotein subunit beta OS=Homo sapiens GN=ETFB PE=1 SV=3 - [ETFB_HUMAN]	27.8	9.45		3		4	
P55084	Trifunctional enzyme subunit beta, mitochondrial OS=Homo sapiens GN=HADHB PE=1 SV=3 - [ECHB_HUMAN]	51.3	9.43	2.29	7	1	8	1
Q05639	Elongation factor 1-alpha 2 OS=Homo sapiens GN=EEF1A2 PE=1 SV=1 - [EF1A2_HUMAN]	50.4	9.16		5		5	
P36957	Dihydrolipoyllysine-residue succinyltransferase component of 2-oxoglutarate dehydrogenase complex, mitochondrial OS=Homo sapiens GN=DLST PE=1 SV=4 - [ODO2_HUMAN]	48.7	9.11	1.66	4	1	4	1
P62269	40S ribosomal protein S18 OS=Homo sapiens GN=RPS18 PE=1 SV=3 - [RS18_HUMAN]	17.7	8.86	4.71	3	2	4	2
Q00325	Phosphate carrier protein, mitochondrial OS=Homo sapiens GN=SLC25A3 PE=1 SV=2 - [MPCP_HUMAN]	40.1	8.84		5		6	
P33121	Long-chain-fatty-acid--CoA ligase 1 OS=Homo sapiens GN=ACSL1 PE=1 SV=1 - [ACSL1_HUMAN]	77.9	8.72		4		5	
Q96I99	Succinate--CoA ligase [GDP-forming] subunit beta, mitochondrial OS=Homo sapiens GN=SUCLG2 PE=1 SV=2 - [SUCB2_HUMAN]	46.5	8.69		4		4	
P45880	Voltage-dependent anion-selective channel protein 2 OS=Homo sapiens GN=VDAC2 PE=1 SV=2 - [VDAC2_HUMAN]	31.5	8.32		3		3	
Q9H223	EH domain-containing protein 4 OS=Homo sapiens GN=EHD4 PE=1 SV=1 - [EHD4_HUMAN]	61.1	8.30		5		6	
O00429	Dynamin-1-like protein OS=Homo sapiens GN=DNM1L PE=1 SV=2 - [DNM1L_HUMAN]	81.8	8.20		6		6	
O60313	Dynamin-like 120 kDa protein, mitochondrial OS=Homo sapiens GN=OPA1 PE=1 SV=3 - [OPA1_HUMAN]	111.6	8.16		4		4	
Q96KK5	Histone H2A type 1-H OS=Homo sapiens GN=HIST1H2AH PE=1 SV=3 - [H2A1H_HUMAN]	13.9	8.15	9.15	3	1	3	2
Q12931	Heat shock protein 75 kDa, mitochondrial OS=Homo sapiens GN=TRAP1 PE=1 SV=3 - [TRAP1_HUMAN]	80.1	8.06		4		4	
P28331	NADH-ubiquinone oxidoreductase 75 kDa subunit, mitochondrial OS=Homo sapiens GN=NDUFS1 PE=1 SV=3 - [NDUS1_HUMAN]	79.4	7.79		6		6	
P42765	3-ketoacyl-CoA thiolase, mitochondrial OS=Homo sapiens GN=ACAA2 PE=1 SV=2 - [THIM_HUMAN]	41.9	7.55		3		3	
P53597	Succinate--CoA ligase [ADP/GDP-forming] subunit alpha, mitochondrial OS=Homo sapiens GN=SUCLG1 PE=1 SV=4 - [SUCA_HUMAN]	36.2	7.53		4		4	
P50461	Cysteine and glycine-rich protein 3 OS=Homo sapiens GN=CSRP3 PE=1 SV=1 - [CSRP3_HUMAN]	21.0	7.17		2		2	
Q9Y6N5	Sulfide:quinone oxidoreductase, mitochondrial OS=Homo sapiens GN=SQRDL PE=1 SV=1 - [SQRD_HUMAN]	49.9	6.98		4		4	
P00505	Aspartate aminotransferase, mitochondrial OS=Homo sapiens GN=GOT2 PE=1 SV=3 - [AATM_HUMAN]	47.5	6.92		3		3	
P49748	Very long-chain specific acyl-CoA dehydrogenase, mitochondrial OS=Homo sapiens GN=ACADVL PE=1 SV=1 - [ACADV_HUMAN]	70.3	6.86		3		3	
O75323	Protein NipSnap homolog 2 OS=Homo sapiens GN=GBAS PE=1 SV=1 - [NIPS2_HUMAN]	33.7	6.79		2		3	
P22695	Cytochrome b-c1 complex subunit 2, mitochondrial OS=Homo sapiens GN=UQCRC2 PE=1 SV=3 - [QCR2_HUMAN]	48.4	6.61		2		3	
P35232	Prohibitin OS=Homo sapiens GN=PHB PE=1 SV=1 - [PHB_HUMAN]	29.8	6.45		3		3	
Q14896	Myosin-binding protein C, cardiac-type OS=Homo sapiens GN=MYBPC3 PE=1 SV=4 - [MYPC3_HUMAN]	140.7	6.33		3		3	
P20073	Annexin A7 OS=Homo sapiens GN=ANXA7 PE=1 SV=3 - [ANXA7_HUMAN]	52.7	6.32		3		3	
Q92523	Carnitine O-palmitoyltransferase 1, muscle isoform OS=Homo sapiens GN=CPT1B PE=1 SV=2 - [CPT1B_HUMAN]	87.7	6.14		2		3	
P04075	Fructose-bisphosphate aldolase A OS=Homo sapiens GN=ALDOA PE=1 SV=2 - [ALDOA_HUMAN]	39.4	6.05		3		3	
P48047	ATP synthase subunit O, mitochondrial OS=Homo sapiens GN=ATP5O PE=1 SV=1 - [ATPO_HUMAN]	23.3	5.95	2.73	2	1	2	1
Q6YN16	Hydroxysteroid dehydrogenase-like protein 2 OS=Homo sapiens GN=HSDL2 PE=1 SV=1 - [HSDL2_HUMAN]	45.4	5.69		2		2	
P61247	40S ribosomal protein S3a OS=Homo sapiens GN=RPS3A PE=1 SV=2 - [RS3A_HUMAN]	29.9	5.65		3		3	
Q16777	Histone H2A type 2-C OS=Homo sapiens GN=HIST2H2AC PE=1 SV=4 - [H2A2C_HUMAN]	14.0	5.64	4.84	3	1	3	1
P62805	Histone H4 OS=Homo sapiens GN=HIST1H4A PE=1 SV=2 - [H4_HUMAN]	11.4	5.62		3		3	
O43707	Alpha-actinin-4 OS=Homo sapiens GN=ACTN4 PE=1 SV=2 - [ACTN4_HUMAN]	104.8	5.50		3		3	
P45954	Short/branched chain specific acyl-CoA dehydrogenase, mitochondrial OS=Homo sapiens GN=ACADSB PE=1 SV=1 - [ACDSB_HUMAN]	47.5	5.47		2		2	
P24752	Acetyl-CoA acetyltransferase, mitochondrial OS=Homo sapiens GN=ACAT1 PE=1 SV=1 - [THIL_HUMAN]	45.2	5.31		2		3	
O95822	Malonyl-CoA decarboxylase, mitochondrial OS=Homo sapiens GN=MLYCD PE=1 SV=3 - [DCMC_HUMAN]	55.0	5.25		3		3	
P04899	Guanine nucleotide-binding protein G(i) subunit alpha-2 OS=Homo sapiens GN=GNAI2 PE=1 SV=3 - [GNAI2_HUMAN]	40.4	5.25		2		2	
Q16134	Electron transfer flavoprotein-ubiquinone oxidoreductase, mitochondrial OS=Homo sapiens GN=ETFDH PE=1 SV=2 - [ETFD_HUMAN]	68.5	5.21		2		2	
Q92506	Estradiol 17-beta-dehydrogenase 8 OS=Homo sapiens GN=HSD17B8 PE=1 SV=2 - [DHB8_HUMAN]	27.0	5.14		2		2	
P0CG48	Polyubiquitin-C OS=Homo sapiens GN=UBC PE=1 SV=3 - [UBC_HUMAN]	77.0	5.06	2.91	2	1	2	1
P31930	Cytochrome b-c1 complex subunit 1, mitochondrial OS=Homo sapiens GN=UQCRC1 PE=1 SV=3 - [QCR1_HUMAN]	52.6	4.97		3		3	
P81605	Dermcidin OS=Homo sapiens GN=DCD PE=1 SV=2 - [DCD_HUMAN]	11.3	4.83	5.04	2	2	2	2
P09622	Dihydrolipoyl dehydrogenase, mitochondrial OS=Homo sapiens GN=DLD PE=1 SV=2 - [DLDH_HUMAN]	54.1	4.80		2		2	
Q7Z3D6	UPF0317 protein C14orf159, mitochondrial OS=Homo sapiens GN=C14orf159 PE=1 SV=2 - [CN159_HUMAN]	66.4	4.79		2		2	
O95831	Apoptosis-inducing factor 1, mitochondrial OS=Homo sapiens GN=AIFM1 PE=1 SV=1 - [AIFM1_HUMAN]	66.9	4.70		2		2	
P21796	Voltage-dependent anion-selective channel protein 1 OS=Homo sapiens GN=VDAC1 PE=1 SV=2 - [VDAC1_HUMAN]	30.8	4.63		2		2	
Q9UKU7	Isobutyryl-CoA dehydrogenase, mitochondrial OS=Homo sapiens GN=ACAD8 PE=1 SV=1 - [ACAD8_HUMAN]	45.0	4.63		2		2	
O76031	ATP-dependent Clp protease ATP-binding subunit clpX-like, mitochondrial OS=Homo sapiens GN=CLPX PE=1 SV=2 - [CLPX_HUMAN]	69.2	4.63		2		2	
P62701	40S ribosomal protein S4, X isoform OS=Homo sapiens GN=RPS4X PE=1 SV=2 - [RS4X_HUMAN]	29.6	4.59	6.35	2	3	2	3
Q8NI60	Atypical kinase COQ8A, mitochondrial OS=Homo sapiens GN=COQ8A PE=1 SV=1 - [COQ8A_HUMAN]	71.9	4.44		3		3	
P62277	40S ribosomal protein S13 OS=Homo sapiens GN=RPS13 PE=1 SV=2 - [RS13_HUMAN]	17.2	4.36	3.02	3	2	4	2
P61981	14-3-3 protein gamma OS=Homo sapiens GN=YWHAG PE=1 SV=2 - [1433G_HUMAN]	28.3	4.30		4		4	
P61978	Heterogeneous nuclear ribonucleoprotein K OS=Homo sapiens GN=HNRNPK PE=1 SV=1 - [HNRPK_HUMAN]	50.9	4.24		2		2	
O95299	NADH dehydrogenase [ubiquinone] 1 alpha subcomplex subunit 10, mitochondrial OS=Homo sapiens GN=NDUFA10 PE=1 SV=1 - [NDUAA_HUMAN]	40.7	4.14		2		2	
P61626	Lysozyme C OS=Homo sapiens GN=LYZ PE=1 SV=1 - [LYSC_HUMAN]	16.5	4.01	2.03	3	1	3	1
P14923	Junction plakoglobin OS=Homo sapiens GN=JUP PE=1 SV=3 - [PLAK_HUMAN]	81.7	4.00	1.95	3	1	3	1
O00483	Cytochrome c oxidase subunit NDUFA4 OS=Homo sapiens GN=NDUFA4 PE=1 SV=1 - [NDUA4_HUMAN]	9.4	3.98		2		2	
P63104	14-3-3 protein zeta/delta OS=Homo sapiens GN=YWHAZ PE=1 SV=1 - [1433Z_HUMAN]	27.7	3.97		3		3	
Q96RQ3	Methylcrotonoyl-CoA carboxylase subunit alpha, mitochondrial OS=Homo sapiens GN=MCCC1 PE=1 SV=3 - [MCCA_HUMAN]	80.4	3.68		2		2	

P01857	Immunoglobulin heavy constant gamma 1 OS=Homo sapiens GN=IGHG1 PE=1 SV=1 - [IGHG1_HUMAN]	36.1	3.63	12.59	2	3	5	9
O43837	Isocitrate dehydrogenase [NAD] subunit beta, mitochondrial OS=Homo sapiens GN=IDH3B PE=1 SV=2 - [IDH3B_HUMAN]	42.2	3.54		2		2	
P15924	Desmoplakin OS=Homo sapiens GN=DSP PE=1 SV=3 - [DESP_HUMAN]	331.6	3.43	1.73	2	2	2	2
P49821	NADH dehydrogenase [ubiquinone] flavoprotein 1, mitochondrial OS=Homo sapiens GN=NDUFV1 PE=1 SV=4 - [NDUV1_HUMAN]	50.8	3.26		1		1	
O75390	Citrate synthase, mitochondrial OS=Homo sapiens GN=CS PE=1 SV=2 - [CISY_HUMAN]	51.7	3.13		1		1	
Q8N983	39S ribosomal protein L43, mitochondrial OS=Homo sapiens GN=MRPL43 PE=1 SV=1 - [RM43_HUMAN]	23.4	2.93		1		1	
P22033	Methylmalonyl-CoA mutase, mitochondrial OS=Homo sapiens GN=MUT PE=1 SV=4 - [MUTA_HUMAN]	83.1	2.92		4		4	
Q9HCC0	Methylcrotonoyl-CoA carboxylase beta chain, mitochondrial OS=Homo sapiens GN=MCCC2 PE=1 SV=1 - [MCCB_HUMAN]	61.3	2.90		1		1	
P21953	2-oxoisovalerate dehydrogenase subunit beta, mitochondrial OS=Homo sapiens GN=BCKDHB PE=1 SV=2 - [ODBB_HUMAN]	43.1	2.86		1		1	
P13637	Sodium/potassium-transporting ATPase subunit alpha-3 OS=Homo sapiens GN=ATP1A3 PE=1 SV=3 - [AT1A3_HUMAN]	111.7	2.81		1		1	
Q9NVI7	**ATPase family AAA domain-containing protein 3A OS=Homo sapiens GN=ATAD3A PE=1 SV=2 - [ATD3A_HUMAN]**	71.3	2.71		1		1	
Q02413	Desmoglein-1 OS=Homo sapiens GN=DSG1 PE=1 SV=2 - [DSG1_HUMAN]	113.7	2.64	2.28	1	1	1	1
Q96AN5	Transmembrane protein 143 OS=Homo sapiens GN=TMEM143 PE=2 SV=1 - [TM143_HUMAN]	51.7	2.55		1		1	
A6NMY6	Putative annexin A2-like protein OS=Homo sapiens GN=ANXA2P2 PE=5 SV=2 - [AXA2L_HUMAN]	38.6	2.54	2.15	1	1	1	1
Q9BQE3	Tubulin alpha-1C chain OS=Homo sapiens GN=TUBA1C PE=1 SV=1 - [TBA1C_HUMAN]	49.9	2.54		1		1	
P62917	60S ribosomal protein L8 OS=Homo sapiens GN=RPL8 PE=1 SV=2 - [RL8_HUMAN]	28.0	2.51		1		1	
P61088	Ubiquitin-conjugating enzyme E2 N OS=Homo sapiens GN=UBE2N PE=1 SV=1 - [UBE2N_HUMAN]	17.1	2.49		2		2	
P19429	Troponin I, cardiac muscle OS=Homo sapiens GN=TNNI3 PE=1 SV=3 - [TNNI3_HUMAN]	24.0	2.48		1		1	
P68371	Tubulin beta-4B chain OS=Homo sapiens GN=TUBB4B PE=1 SV=1 - [TBB4B_HUMAN]	49.8	2.48		1		1	
P68366	Tubulin alpha-4A chain OS=Homo sapiens GN=TUBA4A PE=1 SV=1 - [TBA4A_HUMAN]	49.9	2.47		1		1	
Q16698	2,4-dienoyl-CoA reductase, mitochondrial OS=Homo sapiens GN=DECR1 PE=1 SV=1 - [DECR_HUMAN]	36.0	2.43		1		1	
P11310	Medium-chain specific acyl-CoA dehydrogenase, mitochondrial OS=Homo sapiens GN=ACADM PE=1 SV=1 - [ACADM_HUMAN]	46.6	2.37		3		3	
O60814	Histone H2B type 1-K OS=Homo sapiens GN=HIST1H2BK PE=1 SV=3 - [H2B1K_HUMAN]	13.9	2.31		1		1	
P40926	Malate dehydrogenase, mitochondrial OS=Homo sapiens GN=MDH2 PE=1 SV=3 - [MDHM_HUMAN]	35.5	2.29		1		1	
P61026	Ras-related protein Rab-10 OS=Homo sapiens GN=RAB10 PE=1 SV=1 - [RAB10_HUMAN]	22.5	2.28		1		1	
Q8WUM4	Programmed cell death 6-interacting protein OS=Homo sapiens GN=PDCD6IP PE=1 SV=1 - [PDC6I_HUMAN]	96.0	2.28		1		1	
P31937	3-hydroxyisobutyrate dehydrogenase, mitochondrial OS=Homo sapiens GN=HIBADH PE=1 SV=2 - [3HIDH_HUMAN]	35.3	2.26		1		1	
Q9NUB1	Acetyl-coenzyme A synthetase 2-like, mitochondrial OS=Homo sapiens GN=ACSS1 PE=1 SV=2 - [ACS2L_HUMAN]	74.8	2.22		2		2	
Q8IWX7	Protein unc-45 homolog B OS=Homo sapiens GN=UNC45B PE=1 SV=1 - [UN45B_HUMAN]	103.7	2.21		1		1	
Q86TX2	Acyl-coenzyme A thioesterase 1 OS=Homo sapiens GN=ACOT1 PE=1 SV=1 - [ACOT1_HUMAN]	46.2	2.18		1		1	
Q9BQ69	O-acetyl-ADP-ribose deacetylase MACROD1 OS=Homo sapiens GN=MACROD1 PE=1 SV=2 - [MACD1_HUMAN]	35.5	2.11		1		1	
P0C0L4	Complement C4-A OS=Homo sapiens GN=C4A PE=1 SV=2 - [CO4A_HUMAN]	192.7	2.10		2		2	
Q96DV4	39S ribosomal protein L38, mitochondrial OS=Homo sapiens GN=MRPL38 PE=1 SV=2 - [RM38_HUMAN]	44.6	2.10		1		1	
P48059	LIM and senescent cell antigen-like-containing domain protein 1 OS=Homo sapiens GN=LIMS1 PE=1 SV=4 - [LIMS1_HUMAN]	37.2	2.10		1		1	
P47985	Cytochrome b-c1 complex subunit Rieske, mitochondrial OS=Homo sapiens GN=UQCRFS1 PE=1 SV=2 - [UCRI_HUMAN]	29.6	2.08		1		1	
P30042	ES1 protein homolog, mitochondrial OS=Homo sapiens GN=C21orf33 PE=1 SV=3 - [ES1_HUMAN]	28.2	2.08		1		1	
P49406	39S ribosomal protein L19, mitochondrial OS=Homo sapiens GN=MRPL19 PE=1 SV=2 - [RM19_HUMAN]	33.5	2.07		1		1	
P06858	Lipoprotein lipase OS=Homo sapiens GN=LPL PE=1 SV=1 - [LIPL_HUMAN]	53.1	2.07		1		1	
P04040	Catalase OS=Homo sapiens GN=CAT PE=1 SV=3 - [CATA_HUMAN]	59.7	2.06		1		1	
Q96MF6	Coenzyme Q-binding protein COQ10 homolog A, mitochondrial OS=Homo sapiens GN=COQ10A PE=2 SV=2 - [CQ10A_HUMAN]	27.7	2.06		1		1	
Q9BYD6	39S ribosomal protein L1, mitochondrial OS=Homo sapiens GN=MRPL1 PE=1 SV=2 - [RM01_HUMAN]	36.9	2.05		1		1	
P05109	Protein S100-A8 OS=Homo sapiens GN=S100A8 PE=1 SV=1 - [S10A8_HUMAN]	10.8	2.05	2.41	1	1	1	1
Q6ZMU5	Tripartite motif-containing protein 72 OS=Homo sapiens GN=TRIM72 PE=1 SV=2 - [TRI72_HUMAN]	52.7	2.05		1		1	
Q15119	[Pyruvate dehydrogenase (acetyl-transferring)] kinase isozyme 2, mitochondrial OS=Homo sapiens GN=PDK2 PE=1 SV=2 - [PDK2_HUMAN]	46.1	2.02		1		1	
P23786	Carnitine O-palmitoyltransferase 2, mitochondrial OS=Homo sapiens GN=CPT2 PE=1 SV=2 - [CPT2_HUMAN]	73.7	2.01		1		1	
P60842	Eukaryotic initiation factor 4A-I OS=Homo sapiens GN=EIF4A1 PE=1 SV=1 - [IF4A1_HUMAN]	46.1	1.99		2		2	
P30084	Enoyl-CoA hydratase, mitochondrial OS=Homo sapiens GN=ECHS1 PE=1 SV=4 - [ECHM_HUMAN]	31.4	1.98		2		2	
P30153	Serine/threonine-protein phosphatase 2A 65 kDa regulatory subunit A alpha isoform OS=Homo sapiens GN=PPP2R1A PE=1 SV=4 - [2AAA_HUMAN]	65.3	1.97		2		2	
Q00577	Transcriptional activator protein Pur-alpha OS=Homo sapiens GN=PURA PE=1 SV=2 - [PURA_HUMAN]	34.9	1.94		1		1	
Q49AM1	Transcription termination factor 2, mitochondrial OS=Homo sapiens GN=MTERF2 PE=1 SV=2 - [MTEF2_HUMAN]	44.4	1.94		1		1	
P30049	ATP synthase subunit delta, mitochondrial OS=Homo sapiens GN=ATP5D PE=1 SV=2 - [ATPD_HUMAN]	17.5	1.92		1		1	
Q13418	Integrin-linked protein kinase OS=Homo sapiens GN=ILK PE=1 SV=2 - [ILK_HUMAN]	51.4	1.91		1		1	
O14958	Calsequestrin-2 OS=Homo sapiens GN=CASQ2 PE=1 SV=2 - [CASQ2_HUMAN]	46.4	1.88		1		1	
Q9BRJ2	39S ribosomal protein L45, mitochondrial OS=Homo sapiens GN=MRPL45 PE=1 SV=2 - [RM45_HUMAN]	35.3	1.88		1		1	
O14874	[3-methyl-2-oxobutanoate dehydrogenase [lipoamide]] kinase, mitochondrial OS=Homo sapiens GN=BCKDK PE=1 SV=2 - [BCKD_HUMAN]	46.3	1.86		1		1	
Q5T653	39S ribosomal protein L2, mitochondrial OS=Homo sapiens GN=MRPL2 PE=1 SV=2 - [RM02_HUMAN]	33.3	1.85		1		1	
P62906	60S ribosomal protein L10a OS=Homo sapiens GN=RPL10A PE=1 SV=2 - [RL10A_HUMAN]	24.8	1.85		1		1	
O94826	Mitochondrial import receptor subunit TOM70 OS=Homo sapiens GN=TOMM70 PE=1 SV=1 - [TOM70_HUMAN]	67.4	1.85		1		1	
O75439	Mitochondrial-processing peptidase subunit beta OS=Homo sapiens GN=PMPCB PE=1 SV=2 - [MPPB_HUMAN]	54.3	1.84		3		3	
Q9Y4W6	AFG3-like protein 2 OS=Homo sapiens GN=AFG3L2 PE=1 SV=2 - [AFG32_HUMAN]	88.5	1.80		2		2	
Q99496	E3 ubiquitin-protein ligase RING2 OS=Homo sapiens GN=RNF2 PE=1 SV=1 - [RING2_HUMAN]	37.6	1.80		1		1	
P16219	Short-chain specific acyl-CoA dehydrogenase, mitochondrial OS=Homo sapiens GN=ACADS PE=1 SV=1 - [ACADS_HUMAN]	44.3	1.78		1		1	
Q02338	D-beta-hydroxybutyrate dehydrogenase, mitochondrial OS=Homo sapiens GN=BDH1 PE=1 SV=3 - [BDH_HUMAN]	38.1	1.75		1		1	
Q9BTZ2	Dehydrogenase/reductase SDR family member 4 OS=Homo sapiens GN=DHRS4 PE=1 SV=3 - [DHRS4_HUMAN]	29.5	1.75		1		1	
O75155	Cullin-associated NEDD8-dissociated protein 2 OS=Homo sapiens GN=CAND2 PE=1 SV=3 - [CAND2_HUMAN]	135.2	1.74		2		2	
Q14315	Filamin-C OS=Homo sapiens GN=FLNC PE=1 SV=3 - [FLNC_HUMAN]	290.8	1.72		1		1	
P10599	Thioredoxin OS=Homo sapiens GN=TXN PE=1 SV=3 - [THIO_HUMAN]	11.7	1.69		1		1	
Q969S9	Ribosome-releasing factor 2, mitochondrial OS=Homo sapiens GN=GFM2 PE=1 SV=1 - [RRF2M_HUMAN]	86.5	1.67		1		1	
Q709F0	Acyl-CoA dehydrogenase family member 11 OS=Homo sapiens GN=ACAD11 PE=1 SV=2 - [ACD11_HUMAN]	87.2	1.65		1		1	
P56381	ATP synthase subunit epsilon, mitochondrial OS=Homo sapiens GN=ATP5E PE=1 SV=2 - [ATP5E_HUMAN]	5.8	1.63		1		1	
Q9BYD2	39S ribosomal protein L9, mitochondrial OS=Homo sapiens GN=MRPL9 PE=1 SV=2 - [RM09_HUMAN]	30.2	1.61		2		2	
P21333	Filamin-A OS=Homo sapiens GN=FLNA PE=1 SV=4 - [FLNA_HUMAN]	280.6	0.00		1		1	
Q5D862	Filaggrin-2 OS=Homo sapiens GN=FLG2 PE=1 SV=1 - [FILA2_HUMAN]	247.9	0.00		1		1	
Q96EY1	DnaJ homolog subfamily A member 3, mitochondrial OS=Homo sapiens GN=DNAJA3 PE=1 SV=2 - [DNJA3_HUMAN]	52.5	0.00		1		1	
P04406	Glyceraldehyde-3-phosphate dehydrogenase OS=Homo sapiens GN=GAPDH PE=1 SV=3 - [G3P_HUMAN]	36.0	0.00		1		1	
Q96G01	Protein bicaudal D homolog 1 OS=Homo sapiens GN=BICD1 PE=1 SV=3 - [BICD1_HUMAN]	110.7	0.00		1		1	
P28330	Long-chain specific acyl-CoA dehydrogenase, mitochondrial OS=Homo sapiens GN=ACADL PE=1 SV=2 - [ACADL_HUMAN]	47.6	0.00		1		1	
O95433	Activator of 90 kDa heat shock protein ATPase homolog 1 OS=Homo sapiens GN=AHSA1 PE=1 SV=1 - [AHSA1_HUMAN]	38.3	0.00		1		1	
Q8TDM6	Disks large homolog 5 OS=Homo sapiens GN=DLG5 PE=1 SV=4 - [DLG5_HUMAN]	213.7	0.00		1		1	
Q08554	Desmocollin-1 OS=Homo sapiens GN=DSC1 PE=1 SV=2 - [DSC1_HUMAN]	99.9	0.00		1		1	
Q9H4M9	EH domain-containing protein 1 OS=Homo sapiens GN=EHD1 PE=1 SV=2 - [EHD1_HUMAN]	60.6	0.00		1		1	
P16520	Guanine nucleotide-binding protein G(I)/G(S)/G(T) subunit beta-3 OS=Homo sapiens GN=GNB3 PE=1 SV=1 - [GBB3_HUMAN]	37.2	0.00		1		1	
P68431	Histone H3.1 OS=Homo sapiens GN=HIST1H3A PE=1 SV=2 - [H31_HUMAN]	15.4	0.00		1		1	
Q6NVY1	3-hydroxyisobutyryl-CoA hydrolase, mitochondrial OS=Homo sapiens GN=HIBCH PE=1 SV=2 - [HIBCH_HUMAN]	43.5	0.00		1		1	
P51553	Isocitrate dehydrogenase [NAD] subunit gamma, mitochondrial OS=Homo sapiens GN=IDH3G PE=1 SV=1 - [IDH3G_HUMAN]	42.8	0.00		2		2	
Q9BR39	Junctophilin-2 OS=Homo sapiens GN=JPH2 PE=1 SV=2 - [JPH2_HUMAN]	74.2	0.00		2		2	
O43772	Mitochondrial carnitine/acylcarnitine carrier protein OS=Homo sapiens GN=SLC25A20 PE=1 SV=1 - [MCAT_HUMAN]	32.9	0.00		2		2	
Q02252	Methylmalonate-semialdehyde dehydrogenase [acylating], mitochondrial OS=Homo sapiens GN=ALDH6A1 PE=1 SV=2 - [MMSA_HUMAN]	57.8	0.00		2		2	
Q15746	Myosin light chain kinase, smooth muscle OS=Homo sapiens GN=MYLK PE=1 SV=4 - [MYLK_HUMAN]	210.6	0.00		1		1	
P37198	Nuclear pore glycoprotein p62 OS=Homo sapiens GN=NUP62 PE=1 SV=3 - [NUP62_HUMAN]	53.2	0.00		1		1	
Q15166	Serum paraoxonase/lactonase 3 OS=Homo sapiens GN=PON3 PE=1 SV=3 - [PON3_HUMAN]	39.6	0.00		1		1	
P11216	Glycogen phosphorylase, brain form OS=Homo sapiens GN=PYGB PE=1 SV=5 - [PYGB_HUMAN]	96.6	0.00		2		2	
P39023	60S ribosomal protein L3 OS=Homo sapiens GN=RPL3 PE=1 SV=2 - [RL3_HUMAN]	46.1	0.00		1		1	
Q9NYK5	39S ribosomal protein L39, mitochondrial OS=Homo sapiens GN=MRPL39 PE=1 SV=3 - [RM39_HUMAN]	38.7	0.00		1		1	
P62280	40S ribosomal protein S11 OS=Homo sapiens GN=RPS11 PE=1 SV=3 - [RS11_HUMAN]	18.4	0.00		3		3	
P82650	28S ribosomal protein S22, mitochondrial OS=Homo sapiens GN=MRPS22 PE=1 SV=1 - [RT22_HUMAN]	41.3	0.00		1		1	
Q9NR31	GTP-binding protein SAR1a OS=Homo sapiens GN=SAR1A PE=1 SV=1 - [SAR1A_HUMAN]	22.4	0.00		1		1	
Q86TD4	Sarcalumenin OS=Homo sapiens GN=SRL PE=2 SV=2 - [SRCA_HUMAN]	100.7	0.00		1		1	
Q15031	Probable leucine--tRNA ligase, mitochondrial OS=Homo sapiens GN=LARS2 PE=1 SV=2 - [SYLM_HUMAN]	101.9	0.00		2		2	
P55851	Mitochondrial uncoupling protein 2 OS=Homo sapiens GN=UCP2 PE=1 SV=1 - [UCP2_HUMAN]	33.2	0.00		1		1	
Q9Y277	Voltage-dependent anion-selective channel protein 3 OS=Homo sapiens GN=VDAC3 PE=1 SV=1 - [VDAC3_HUMAN]	30.6	0.00		1		1	
P04004	Vitronectin OS=Homo sapiens GN=VTN PE=1 SV=1 - [VTNC_HUMAN]	54.3	0.00		1		1	
O15498	Synaptobrevin homolog YKT6 OS=Homo sapiens GN=YKT6 PE=1 SV=1 - [YKT6_HUMAN]	22.4	0.00		1		1	
P29508	Serpin B3 OS=Homo sapiens GN=SERPINB3 PE=1 SV=2 - [SPB3_HUMAN]	44.5		0.00		2		2
P32119	Peroxiredoxin-2 OS=Homo sapiens GN=PRDX2 PE=1 SV=5 - [PRDX2_HUMAN]	21.9		2.33		1		1
P05089	Arginase-1 OS=Homo sapiens GN=ARG1 PE=1 SV=2 - [ARGI1_HUMAN]	34.7		1.61		1		1
Q6BAA4	Fc receptor-like B OS=Homo sapiens GN=FCRLB PE=1 SV=1 - [FCRLB_HUMAN]	46.9		0.00		1		1
Q8NHJ6	Leukocyte immunoglobulin-like receptor subfamily B member 4 OS=Homo sapiens GN=LILRB4 PE=1 SV=3 - [LIRB4_HUMAN]	49.3		0.00		1		1
Q7L590	Protein MCM10 homolog OS=Homo sapiens GN=MCM10 PE=1 SV=2 - [MCM10_HUMAN]	98.1		0.00		1		1
Q03181	Peroxisome proliferator-activated receptor delta OS=Homo sapiens GN=PPARD PE=1 SV=1 - [PPARD_HUMAN]	49.9		1.76		1		1
A4D1P6	WD repeat-containing protein 91 OS=Homo sapiens GN=WDR91 PE=1 SV=2 - [WDR91_HUMAN]	83.3		0.00		1		1
P01619	Immunoglobulin kappa variable 3-20 OS=Homo sapiens GN=IGKV3-20 PE=1 SV=2 - [KV320_HUMAN]	12.5		0.00		1		1
K7EP34	Uncharacterized protein (Fragment) OS=Homo sapiens PE=4 SV=1 - [K7EP34_HUMAN]	10.6		0.00		1		1
A0A075B6I1	Immunoglobulin lambda variable 4-60 OS=Homo sapiens GN=IGLV4-60 PE=3 SV=1 - [LV460_HUMAN]	13.0		2.30		1		1

Appendix References:

Aguayo A, Estey E, Kantarjian H, Mansouri T, Gidel C, Keating M, Giles F, Estrov Z, Barlogie B, Albitar M (1999) Cellular vascular endothelial growth factor is a predictor of outcome in patients with acute myeloid leukemia. *Blood* 94: 3717-3721

Bartel DP (2004) MicroRNAs: genomics, biogenesis, mechanism, and function. *Cell* 116: 281-297

Choksi KB, Nuss JE, Boylston WH, Rabek JP, Papaconstantinou J (2007) Age-related increases in oxidatively damaged proteins of mouse kidney mitochondrial electron transport chain complexes. *Free Radic Biol Med* 43: 1423-1438

Faul F, Erdfelder E, Lang AG, Buchner A (2007) G*Power 3: a flexible statistical power analysis program for the social, behavioral, and biomedical sciences. *Behav Res Methods* 39: 175-191

Jafarifar F, Yao P, Eswarappa SM, Fox PL (2011) Repression of VEGFA by CA-rich element-binding microRNAs is modulated by hnRNP L. *EMBO J* 30: 1324-1334

Pallotti F, Lenaz G (2007) Isolation and subfractionation of mitochondria from animal cells and tissue culture lines. *Methods Cell Biol* 80: 3-44

Prosser HM, Koike-Yusa H, Cooper JD, Law FC, Bradley A (2011) A resource of vectors and ES cells for targeted deletion of microRNAs in mice. *Nat Biotechnol* 29: 840-845

Spinazzi M, Casarin A, Pertegato V, Salviati L, Angelini C (2012) Assessment of mitochondrial respiratory chain enzymatic activities on tissues and cultured cells. *Nat Protoc* 7: 1235-1246

Wang Z, Zhang XJ, Ji YX, Zhang P, Deng KQ, Gong J, Ren S, Wang X, Chen I, Wang H *et al* (2016) The long noncoding RNA Chaer defines an epigenetic checkpoint in cardiac hypertrophy. *Nat Med* 22: 1131-1139

Wu J, Subbaiah KCV, Xie LH, Jiang F, Khor ES, Mickelsen D, Myers JR, Tang WHW, Yao P (2020) Glutamyl-Prolyl-tRNA Synthetase Regulates Proline-Rich Pro-Fibrotic Protein Synthesis During Cardiac Fibrosis. *Circ Res* 127: 827-846
