# Supplementary material for: MicroRNA‐574 regulates FAM210A expression and influences pathological cardiac remodeling
Source: EMBO Mol Med. 2020 Dec 28;13(2):e12710. doi: 10.15252/emmm.202012710 (PMC7863409; doi:10.15252/emmm.202012710)
Supplement: Supplementary file 2 — Expanded View Figures PDF [file EMMM-13-e12710-s002.pdf]

Expanded View Figures

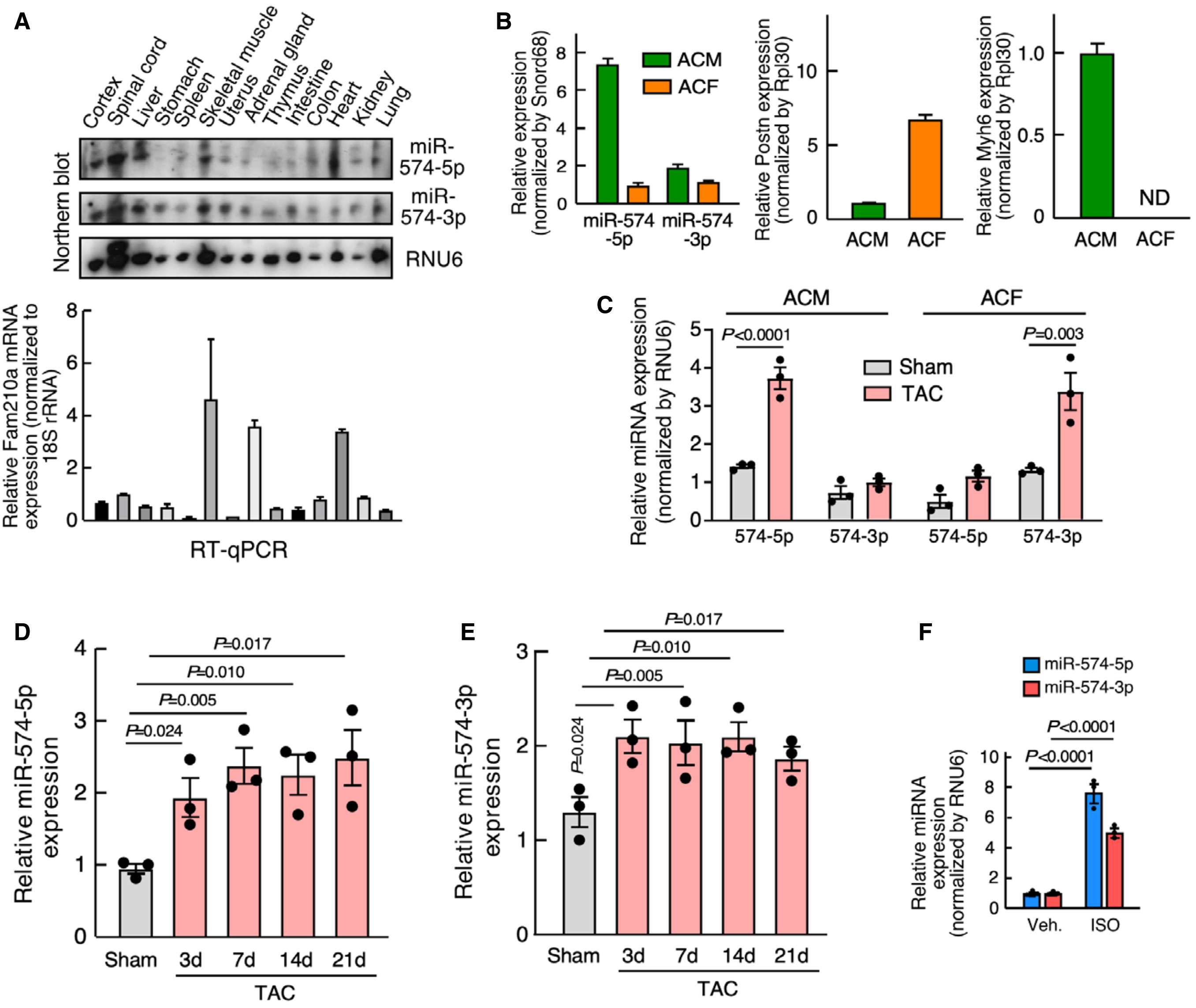

Figure EV1. Expression of miR-574-5p/3p in murine organs and cardiac cells.

A Northern blot detection of miR-574-5p/3p expression and RT–qPCR measurement of *Fam210a* mRNA expression in various murine organs (n = 3).

B miR-574-5p and miR-574-3p expression in adult CM and CF cells isolated from murine hearts of WT mice at baseline. Postn and Myh6 are used as cardiac fibroblast and cardiomyocyte marker, respectively. N = 3 per group.

C miR-574-5p and miR-574-3p expression in adult CM and CF cells isolated from murine hearts of WT mice undergoing TAC surgery after 3 days. N = 3 per group.

D, E Expression of miR-574-5p and miR-574-3p in the heart from mice undergoing TAC surgery at different time points. N = 3 per group.

F ISO induces miR-574-5p and miR-574-3p expression in isolated murine ACMs. N = 3 per group.

Data information: Data were presented as mean ± SEM. P values were calculated by one-way ANOVA with Tukey's multiple comparisons test (C-E) and two-way ANOVA with Tukey's multiple comparisons test (F).
Source data are available online for this figure.

© 2021 The Authors

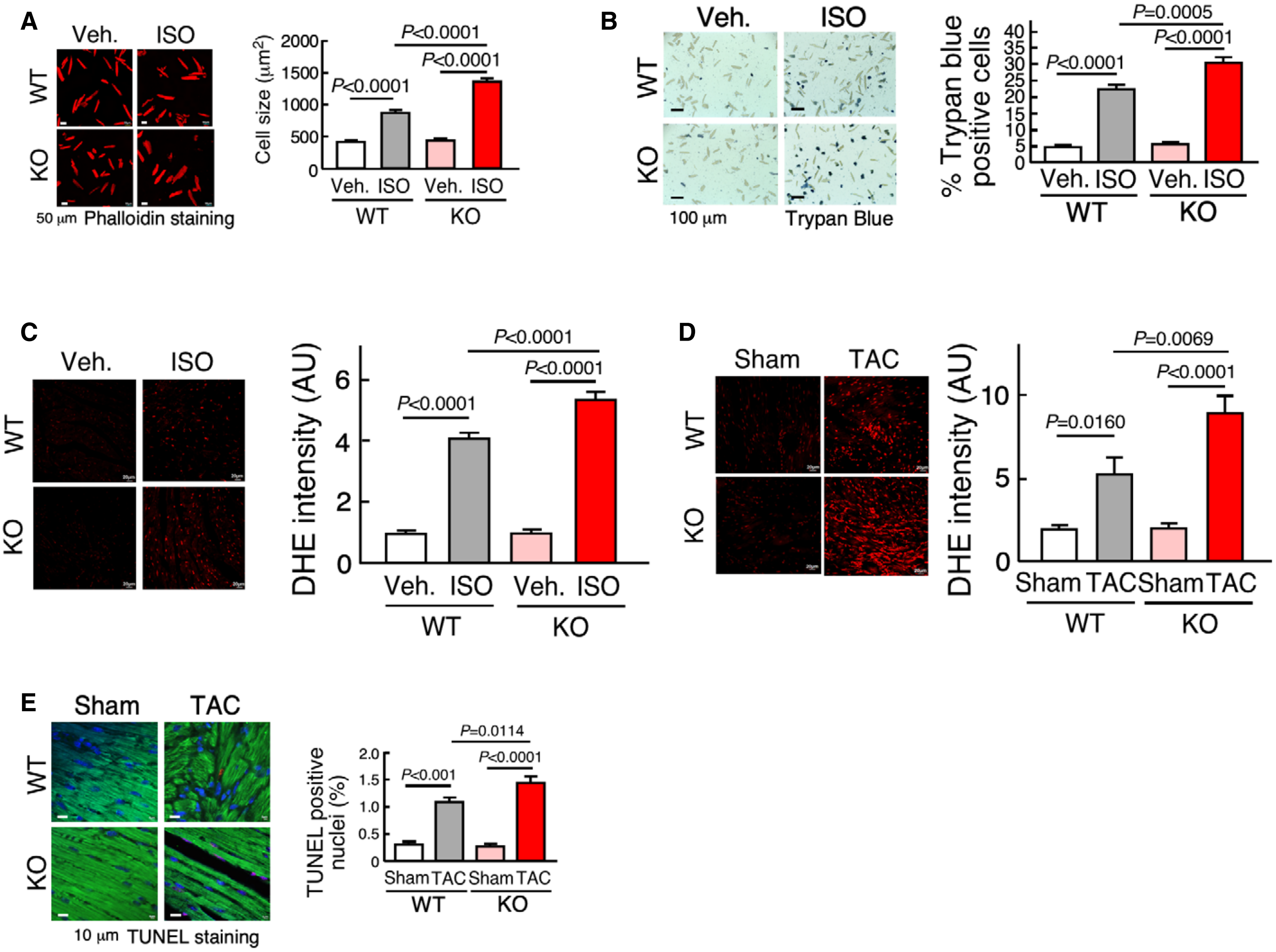

Figure EV2. Phenotypic characterization of isolated CMs and hearts of WT and miR-574$^{-/-}$ mice.

A Phalloidin staining of primary ACMs from WT and miR-574$^{-/-}$ mice in response to isoproterenol (ISO) treatment (10 µM for 24 h). Data are obtained from 3 individual experiments (n> 100 CM cells/group). Scale bar: 50 µm.

B Trypan blue staining of primary ACMs from WT and miR-574$^{-/-}$ mice under ISO (10 µM for 48 h) versus vehicle treatment. N = 5 per group. Scale bar: 100 µm.

C, D DHE staining of frozen sections of hearts from WT and miR-574$^{-/-}$ mice under Veh. and ISO treatment or Sham and TAC operation (4 weeks post-surgery). N = 5 per group for (C) and n = 8 per group for (D). Scale bar: 20 µm.

E TUNEL assay for heart tissue sections from WT and miR-574$^{-/-}$ mice under Sham versus TAC surgery. N = 5 per group. Scale bar: 5 µm.

Data information: Data were presented as mean ± SEM. P values were calculated by Kruskal–Wallis test with Dunn's multiple comparisons test (C) or two-way ANOVA with Tukey's multiple comparisons test (A, B, D, E).
Source data are available online for this figure.

 © 2021 The Authors

Figure EV3. Phenotypic characterization of the therapeutic model using miR-574-5p/3p mimic injection in mice subject to TAC surgery.

A RT–qPCR of miR-574-5p and miR-574-3p after injections of the miRNA mimics for 2 or 4 days. $N = 4$ per group.
B Creatine kinase test and alanine transaminase (ALT) assay in kidneys and livers from miRNA mimic injected mice. $N = 4$ per group. TAC: transverse aortic constriction; Sham is control mock surgery for TAC surgery (no constriction).
C RT–qPCR of hypertrophy and fibrosis marker genes in the hearts of therapeutic mouse models. $N = 5$ per group.
D DHE staining of frozen sections of hearts from WT mice under treatment with miRNA mimics in TAC-induced HF mouse models. Sham operation was used as a control for TAC. $N = 4$ per group. Scale bar: 20 μm.
E ATP level in heart lysates from WT mice under treatment with miRNA mimics in TAC-induced HF mouse models (6 weeks post-surgery). $N = 4$ per group.
F TUNEL assay of murine hearts in the therapeutic models. $N = 4$ per group. Scale bar: 5 μm. Green color: α-actinin immunostaining signal. Red color: TUNEL signal. Blue color: DAPI signal.
G miR-574-5p and miR-574-3p decreased ISO-activated mouse CM hypertrophy. Isolated mouse primary neonatal CMs were stained by FITC phalloidin. The cells were transfected with negative control miRNA, miR-574-3p, and miR-574-5p mimics, followed by ISO (10 μm) treatment for 24 h. N> 100 cells/group. Scale bar: 10 μm. The dashed line in the violin plot shows medium value for the group and the dotted lines represent two quartile lines in each group.
H Mouse primary CF cell proliferation measured by the CyQUANT cell proliferation kit. $N = 4$ per group. NS: not significant (compared to Ctrl-miR group).
I Western blot measurement of α-SMA in TGF-β (10 ng/ml; 24 h) treated mouse primary CF cells. The protein expression was quantified from 4 biological replicates in the right panel. *$P < 0.05$; **$P < 0.01$; ***$P < 0.001$.

Data information: Data were presented as mean ± SEM. All the analyses in (B-F) were performed 6 weeks post-surgery for *in vivo* experiments. *P* values were calculated by one-way ANOVA with Tukey's multiple comparisons test (C-F, H), unpaired two-tailed Student *t* test (G), and two-way ANOVA with Tukey's multiple comparisons test (I). Source data are available online for this figure.

© 2021 The Authors

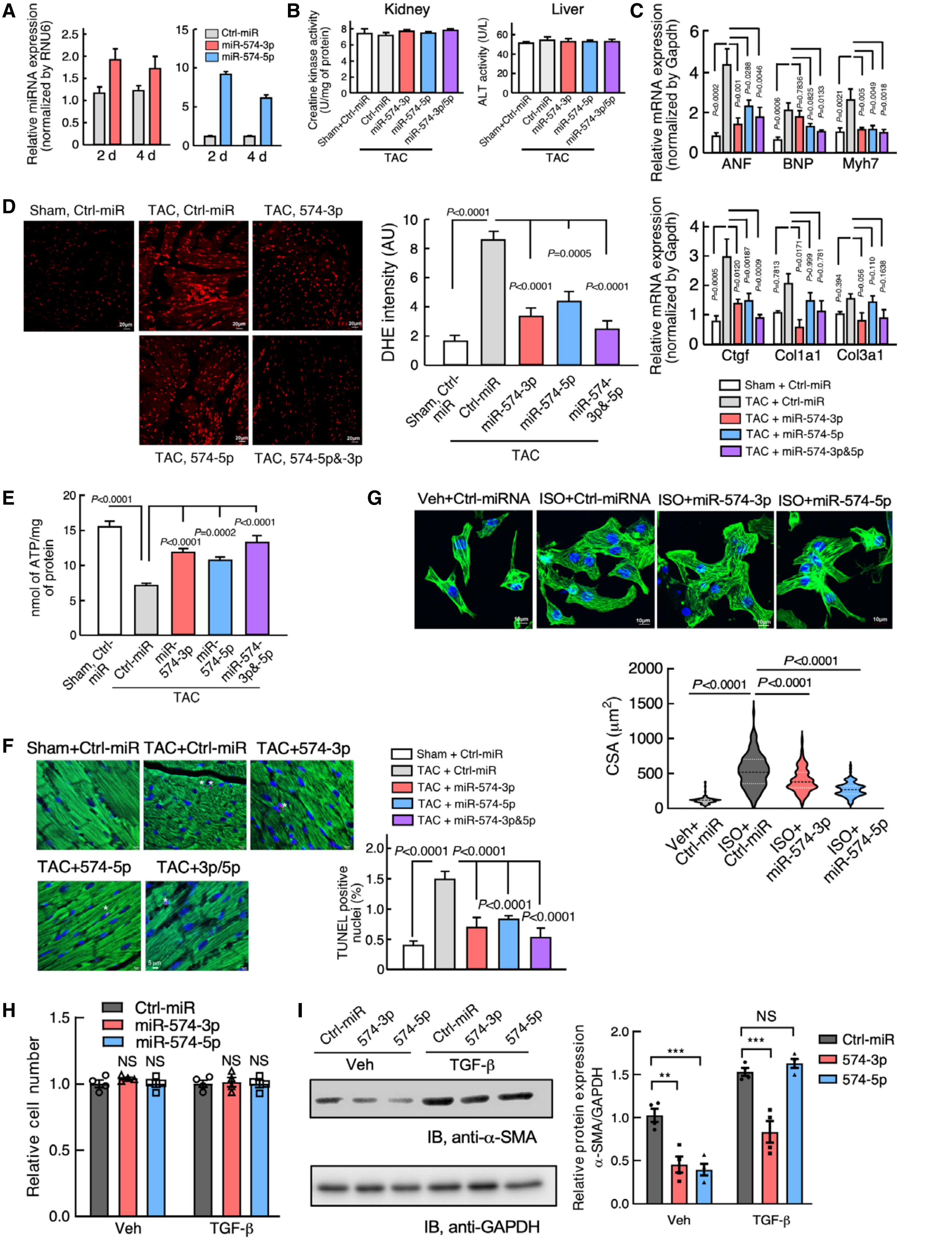

Figure EV3.

© 2021 The Authors

Figure EV4. miR-574-FAM210A axis modulates mitochondrial protein expression and mitochondrial activity in AC16 cardiomyocyte cells.

A, B Quantitative analysis of Western blot data from Fig 8C and D. n.d., not detected. Each experiment was done in triplicates.

C ISO (isoproterenol)-induced cardiomyocyte hypertrophy indicated by phalloidin staining in human AC16 CM cells treated with miRNA mimics (100 nM for 24 h). N> 110 cells/group. Scale bar: 10 μm. The black line in the violin plot shows medium value for the group and the dotted lines represent two quartile lines in each group.

D ISO-induced CM hypertrophy in AC16 cells treated with anti-miR inhibitors (10 μM ISO and 100 nM anti-miR inhibitor treatment for 24 h). $N = 110$–150 cells/group. Scale bar: 10 μm.

E Representative TMRE staining images of AC16 CM cells. Scale bar: 10 μm.

F, G Transmission electron microscopy analysis of mitochondrial surface area and cristae number. 60–150 mitochondria were quantified from 3 hearts. Scale bar: 1 μm and 0.5 μm. $^{**}P < 0.01$.

H Quantitative analysis of Western blot data from Fig 8I. Each experiment was done in triplicates. Control AC16 cells and cells with FAM210A stable overexpression were treated with 10 μM ISO for 24 h, followed by transfection of 100 nM of miRNA mimics. $^{*}P < 0.05$; $^{**}P < 0.01$; $^{***}P < 0.001$.

I Measurement of mitochondrial copy number in AC16 cells transfected with Ctrl-miR, miR-574-5p, miR-574-3p in the presence or absence of FAM210A overexpression. $N = 3$ per group.

Data information: Data were presented as mean ± SEM. P values were calculated by one-way ANOVA (A, B), Kruskal–Wallis test with Dunn's multiple comparisons test (C, D), one-way ANOVA with Tukey's multiple comparisons test (F, G), or two-way ANOVA with Tukey's multiple comparisons test (H).
Source data are available online for this figure.

© 2021 The Authors

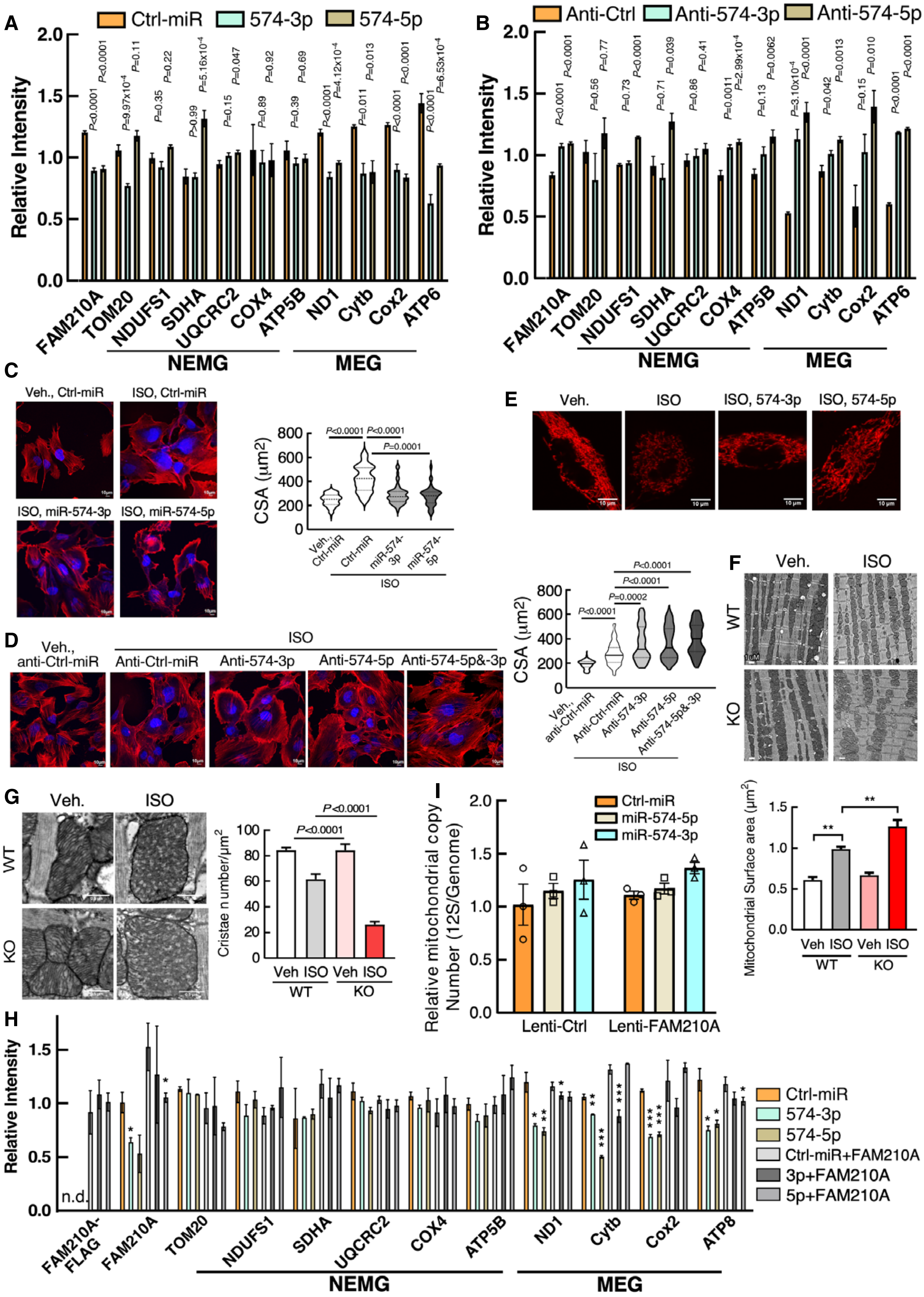

Figure EV4.

© 2021 The Authors

Figure EV5. miR-574-FAM210A axis modulates mitochondrial protein expression and mitochondrial activity in murine hearts.

A, B IF analysis of protein expression of ETC complex protein components in WT and miR-574 KO hearts 3 days after TAC surgery. $n = 5$ hearts per group with> 100 CMs measured per group. Scale bar: 10 μm. The dashed line in the violin plot shows medium value for the group and the dotted lines represent two quartile lines in each group.

C Measurement of mitochondrial copy number in WT and miR-574 KO hearts 3 days after TAC surgery ($n = 4$).

D Measurement of mitochondrial electron transport chain complex enzymatic activities in heart lysates of WT and miR-574 KO mice at 4 weeks after TAC or Sham surgery ($n = 5$ per group).

Data information: Data were presented as mean ± SEM. *P* values were calculated by two-way ANOVA with Tukey's multiple comparisons test (B-D).
Source data are available online for this figure.

© 2021 The Authors

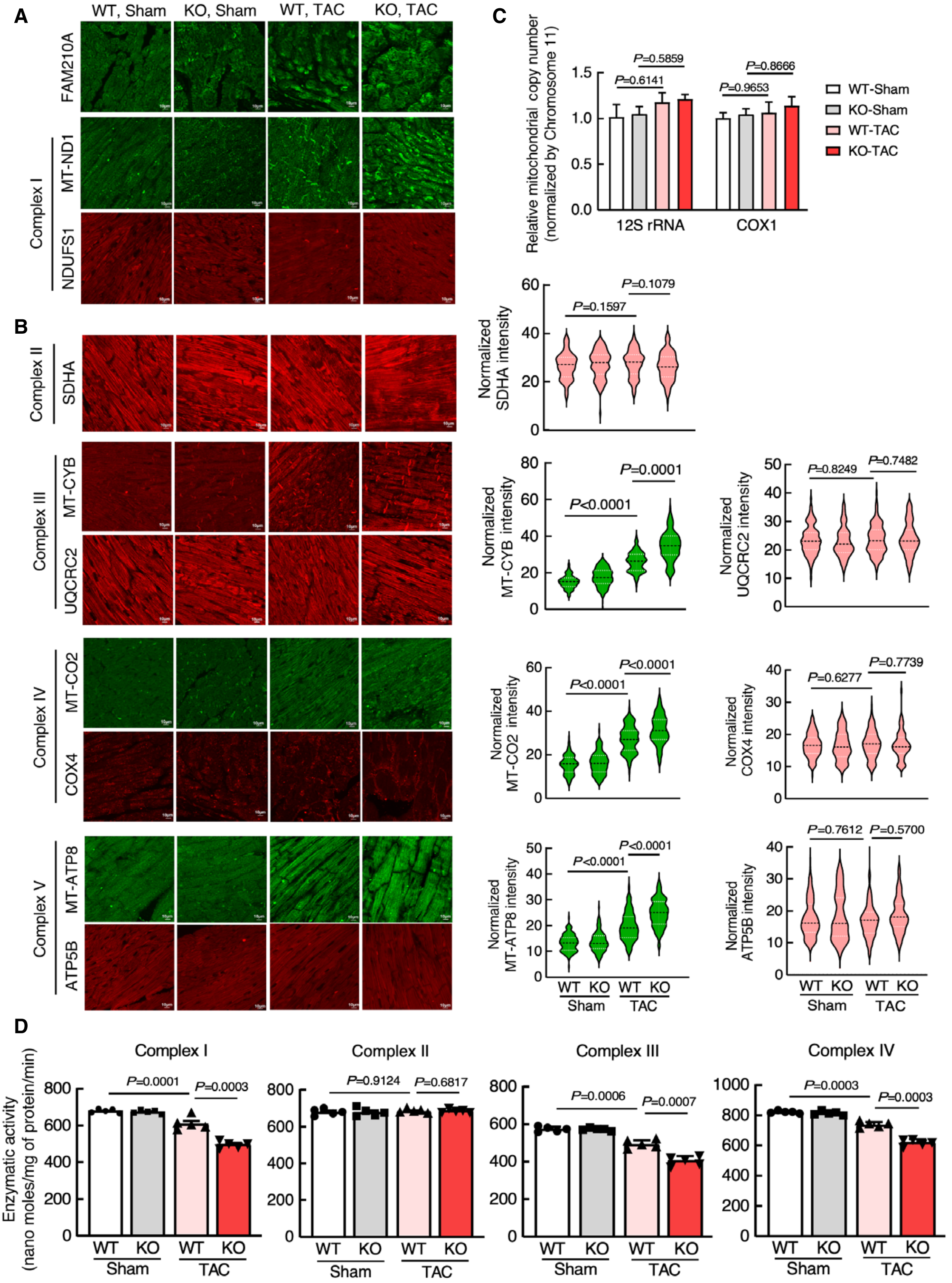

Figure EV5.

 © 2021 The Authors
