# Supplementary figures and images for: MicroRNA‐574 regulates FAM210A expression and influences pathological cardiac remodeling
Source: EMBO Mol Med. 2020 Dec 28;13(2):e12710. doi: 10.15252/emmm.202012710 (PMC7863409; doi:10.15252/emmm.202012710)

## Slide 1
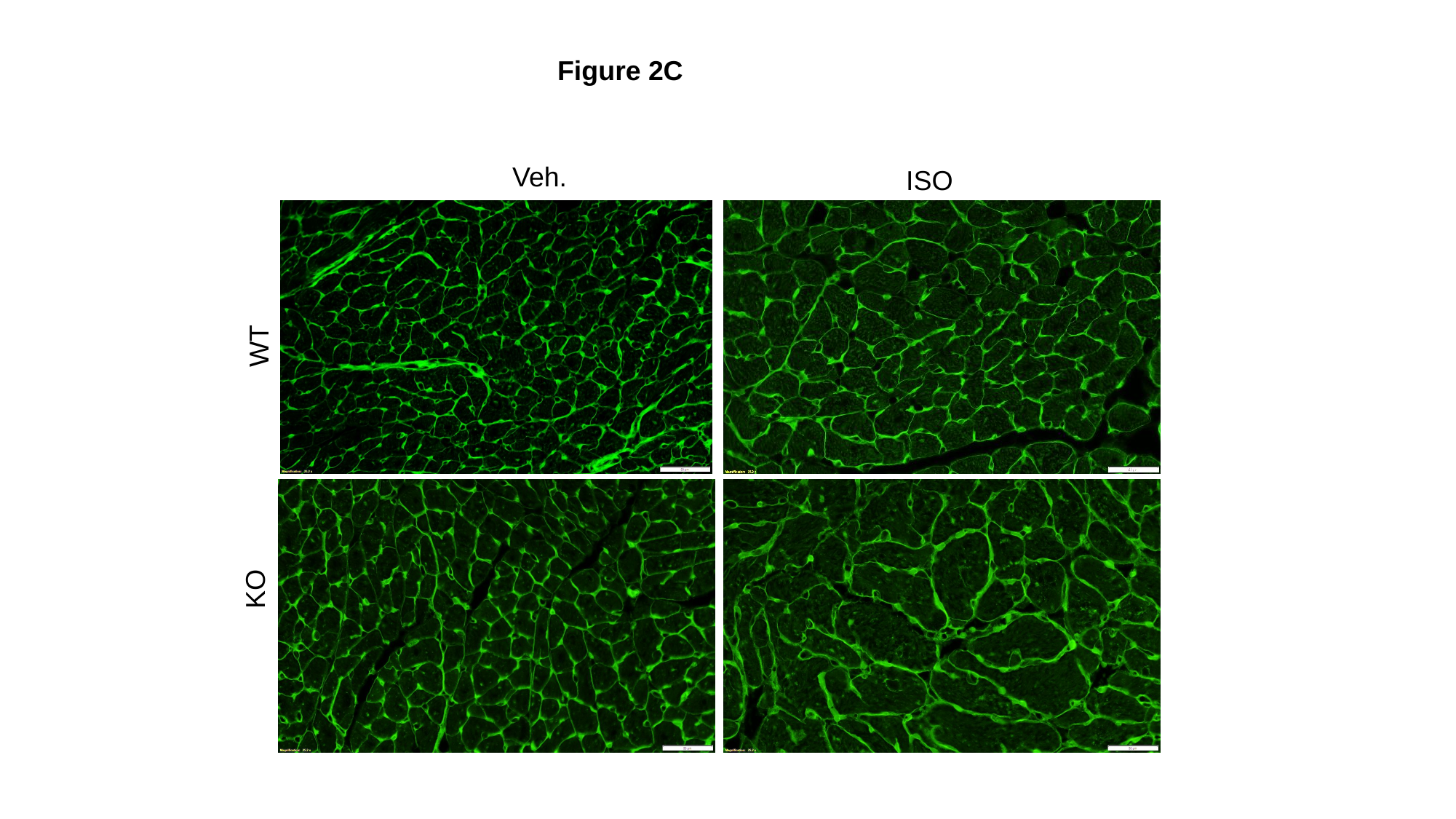

Figure 2C
Veh.
ISO
WT
KO

## Slide 2
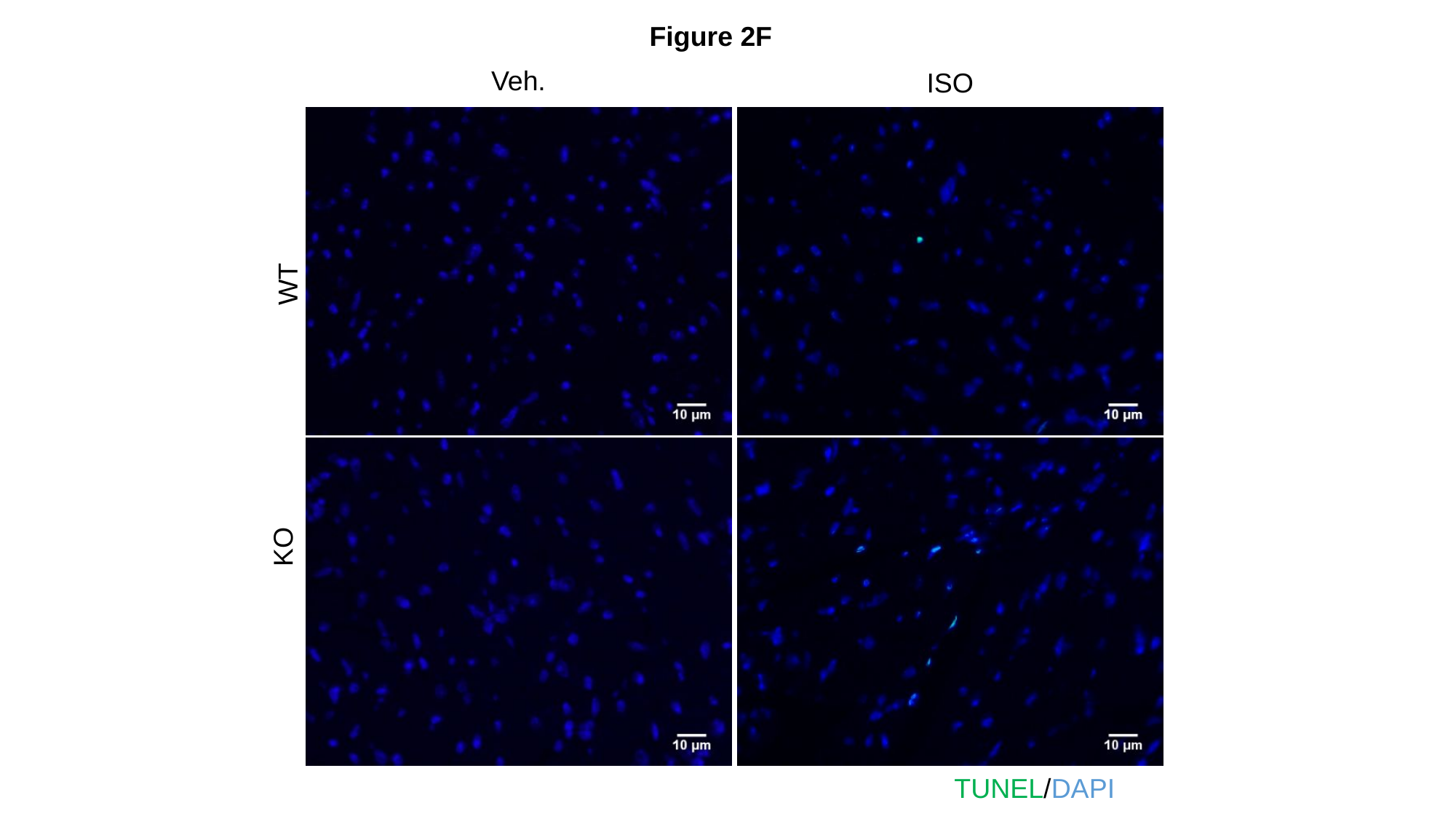

Figure 2F
Veh.
ISO
WT
KO
TUNEL/DAPI

Supplement: Supplementary file 6 — Source Data for Figure 2 [file EMMM-13-e12710-s004.zip › Figure 2.pptx]
