# Supplementary material for: MicroRNA‐574 regulates FAM210A expression and influences pathological cardiac remodeling
Source: EMBO Mol Med. 2020 Dec 28;13(2):e12710. doi: 10.15252/emmm.202012710 (PMC7863409; doi:10.15252/emmm.202012710)
Supplement: Supplementary file 7 — Source Data for Figure 3 [file EMMM-13-e12710-s005.zip › Figure 3.pptx]

## Slide 1
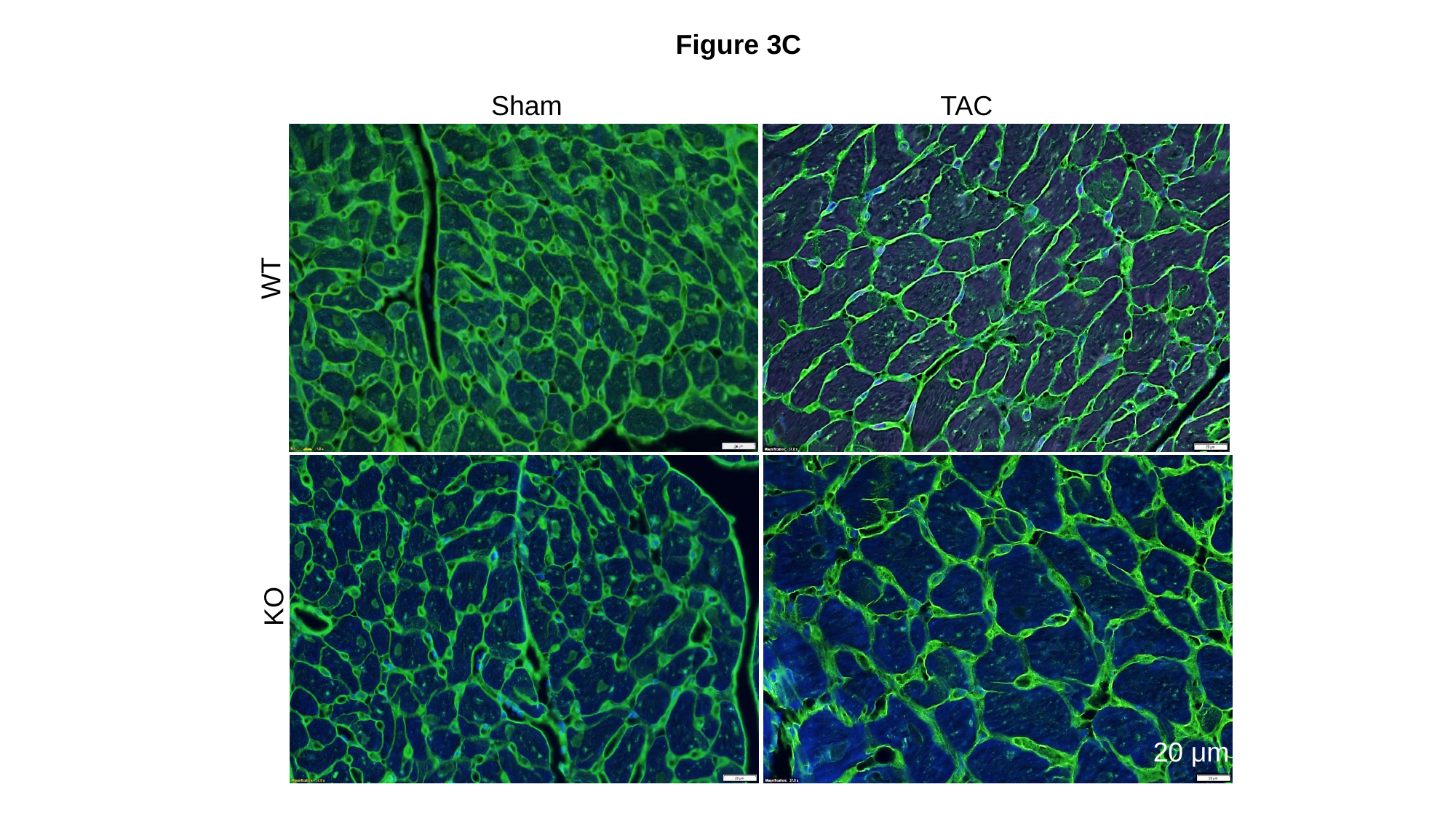

Figure 3C
Sham
TAC
WT
KO
20 μm

## Slide 2
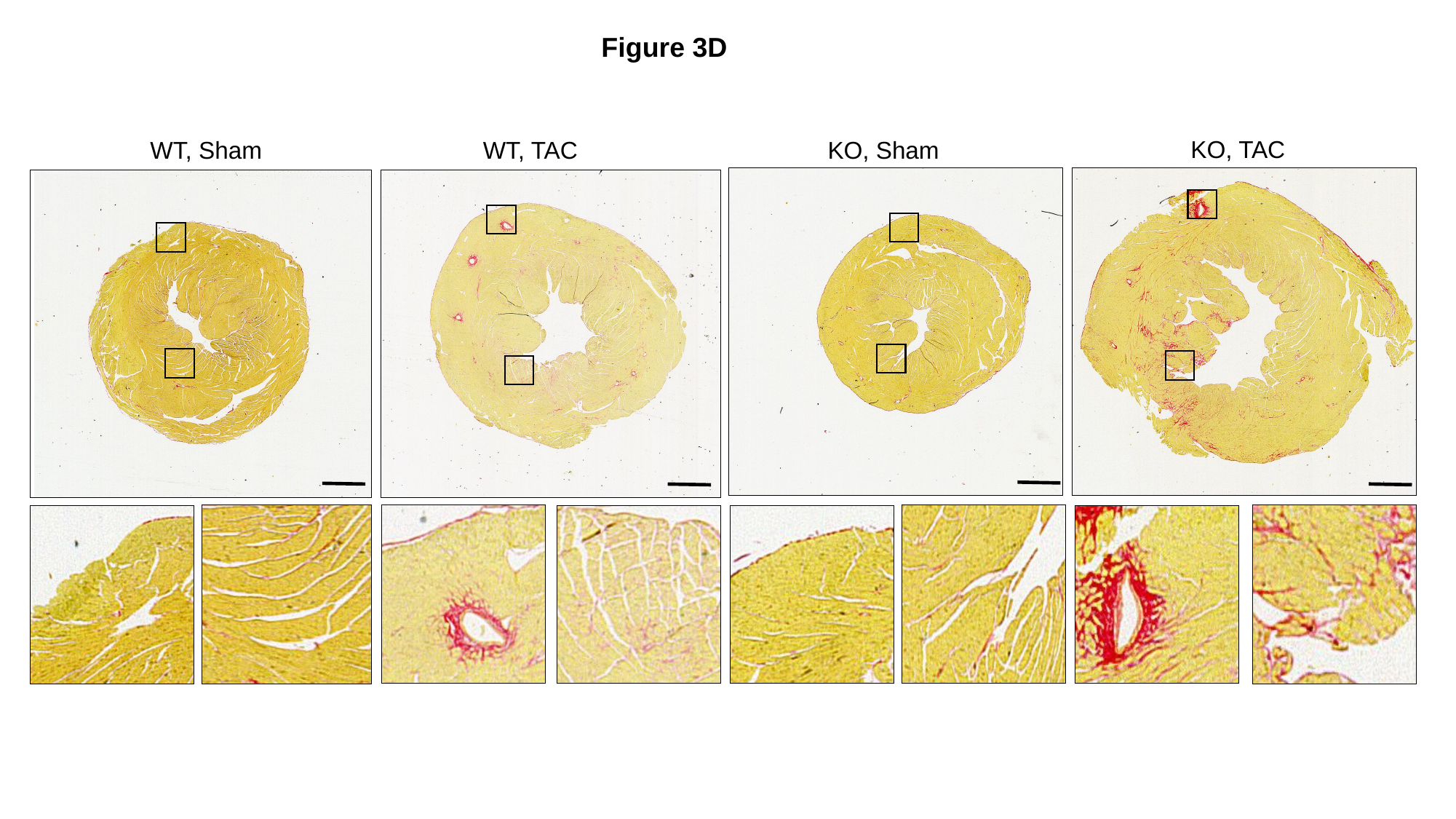

Figure 3D
KO, TAC
WT, Sham
WT, TAC
KO, Sham

## Slide 3
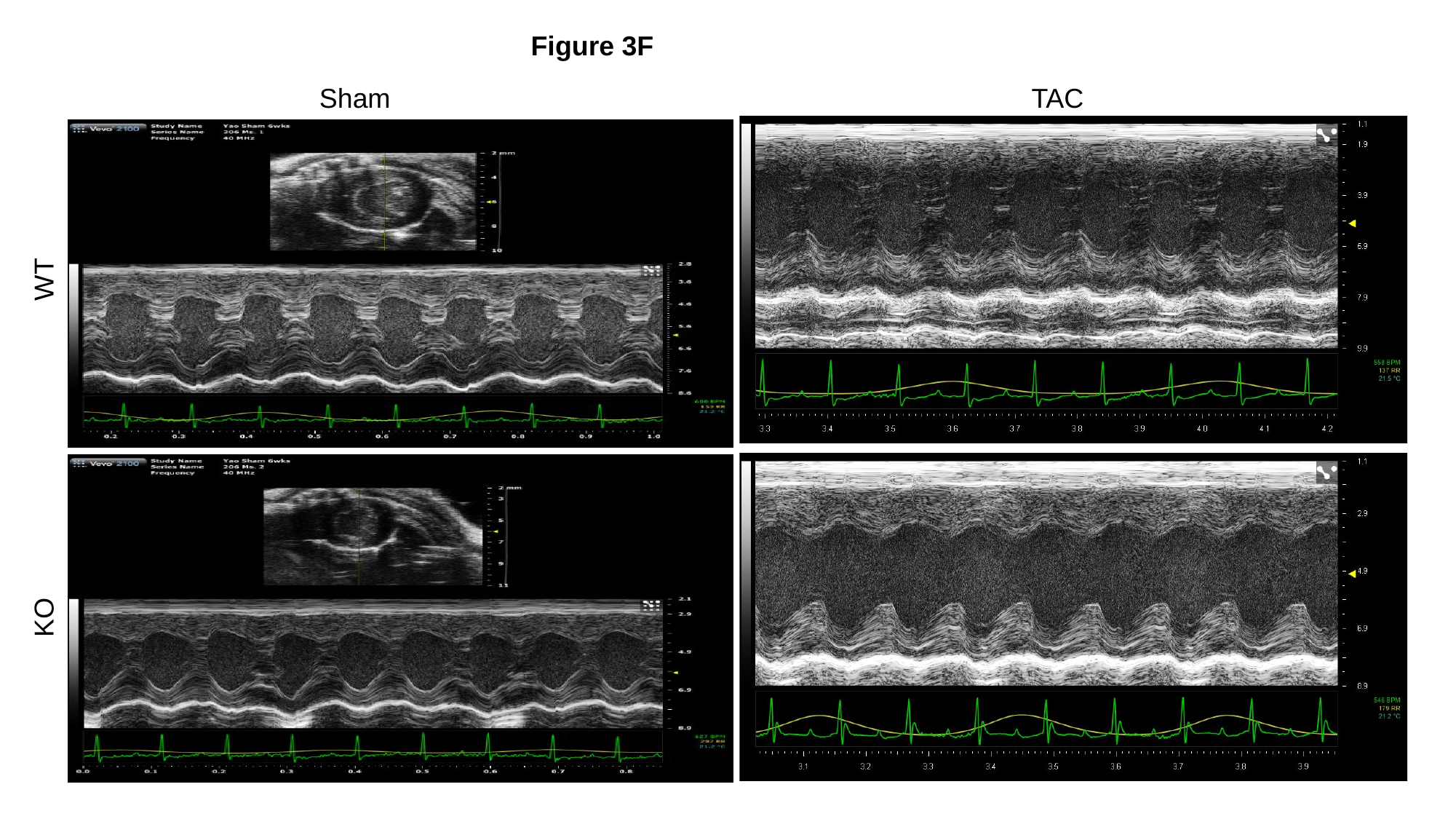

Figure 3F
Sham
TAC
WT
KO
