# Supplementary material for: MicroRNA‐574 regulates FAM210A expression and influences pathological cardiac remodeling
Source: EMBO Mol Med. 2020 Dec 28;13(2):e12710. doi: 10.15252/emmm.202012710 (PMC7863409; doi:10.15252/emmm.202012710)
Supplement: Supplementary file 8 — Source Data for Figure 4 [file EMMM-13-e12710-s006.zip › Figure 4.pptx]

## Slide 1
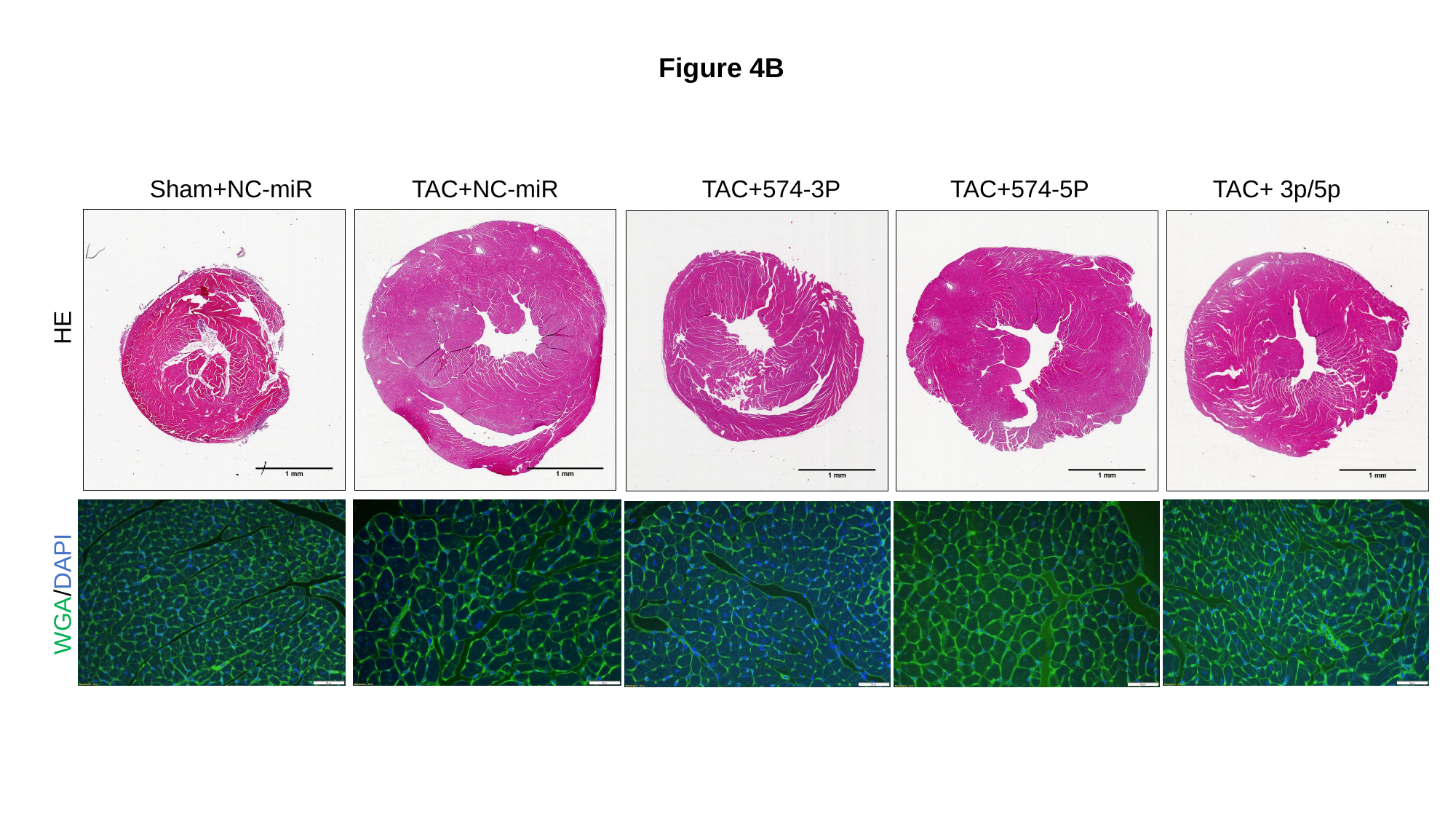

Figure 4B
TAC+574-5P
TAC+ 3p/5p
Sham+NC-miR
TAC+NC-miR
TAC+574-3P
HE
WGA/DAPI

## Slide 2
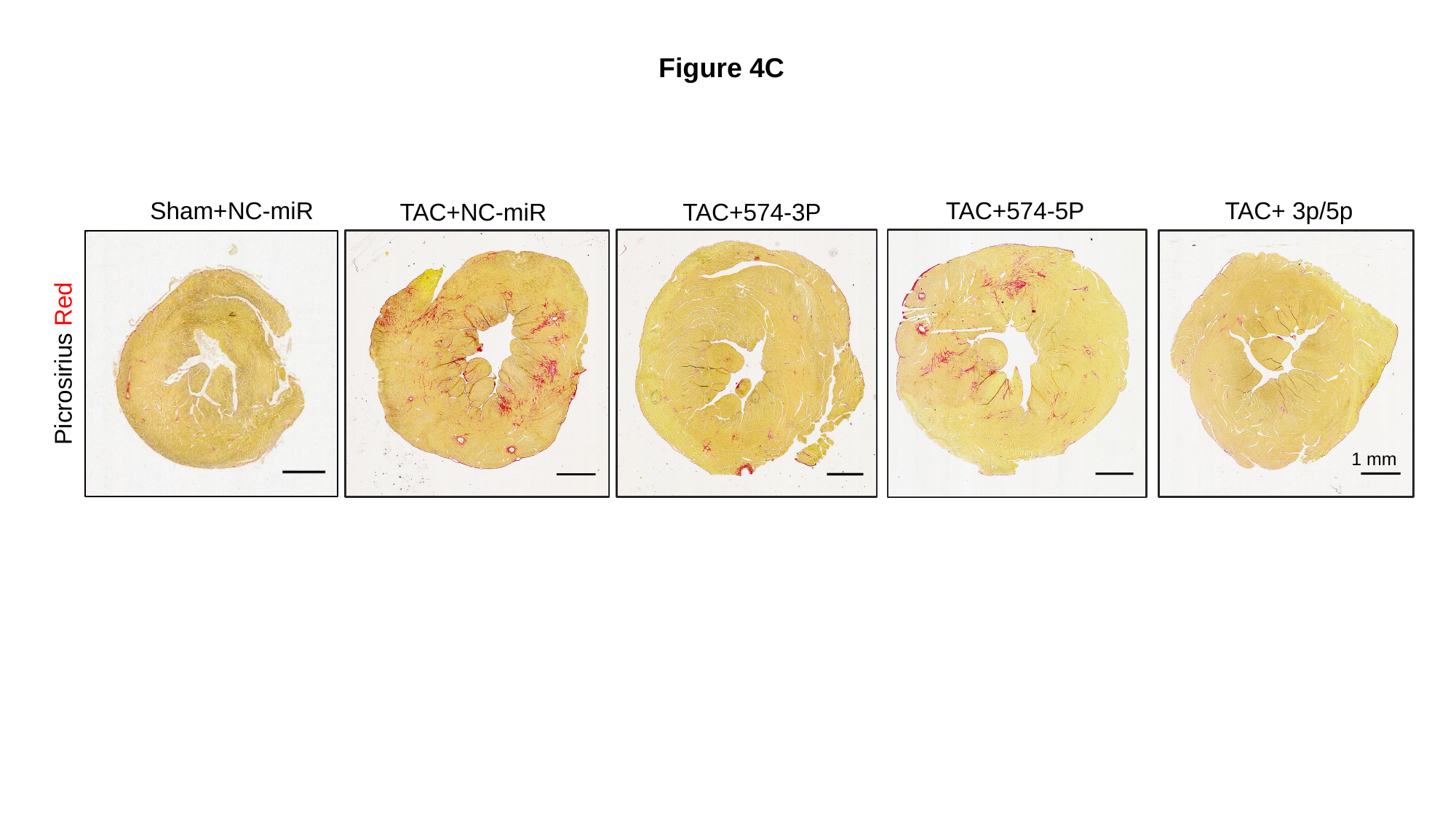

Figure 4C
Sham+NC-miR
TAC+574-5P
TAC+ 3p/5p
TAC+NC-miR
TAC+574-3P
Picrosirius Red
1 mm

## Slide 3
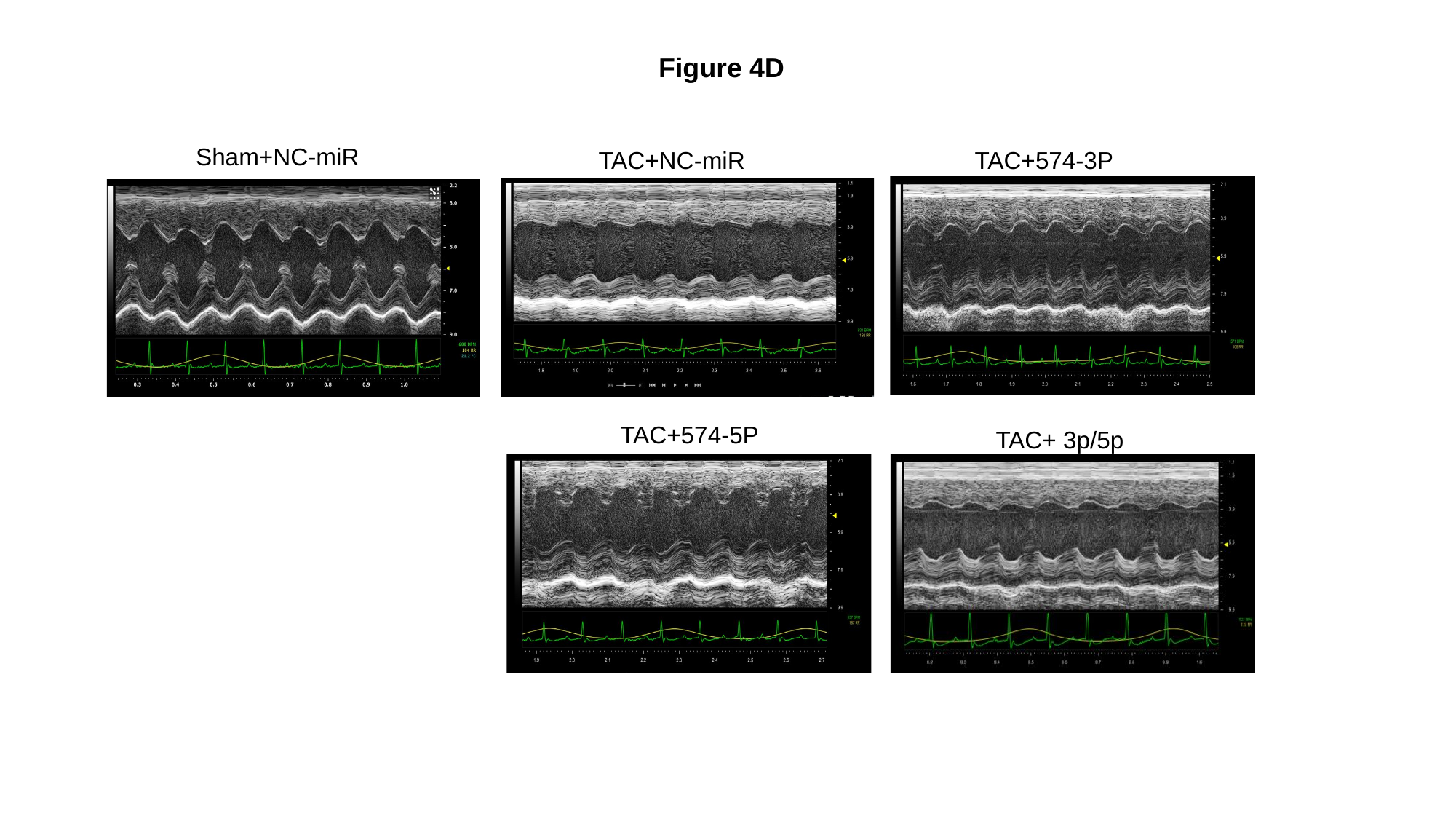

Figure 4D
Sham+NC-miR
TAC+NC-miR
TAC+574-3P
TAC+574-5P
TAC+ 3p/5p
