# Supplementary material for: MicroRNA‐574 regulates FAM210A expression and influences pathological cardiac remodeling
Source: EMBO Mol Med. 2020 Dec 28;13(2):e12710. doi: 10.15252/emmm.202012710 (PMC7863409; doi:10.15252/emmm.202012710)
Supplement: Supplementary file 9 — Source Data for Figure 6 [file EMMM-13-e12710-s007.zip › Figure 6.pptx]

## Slide 1
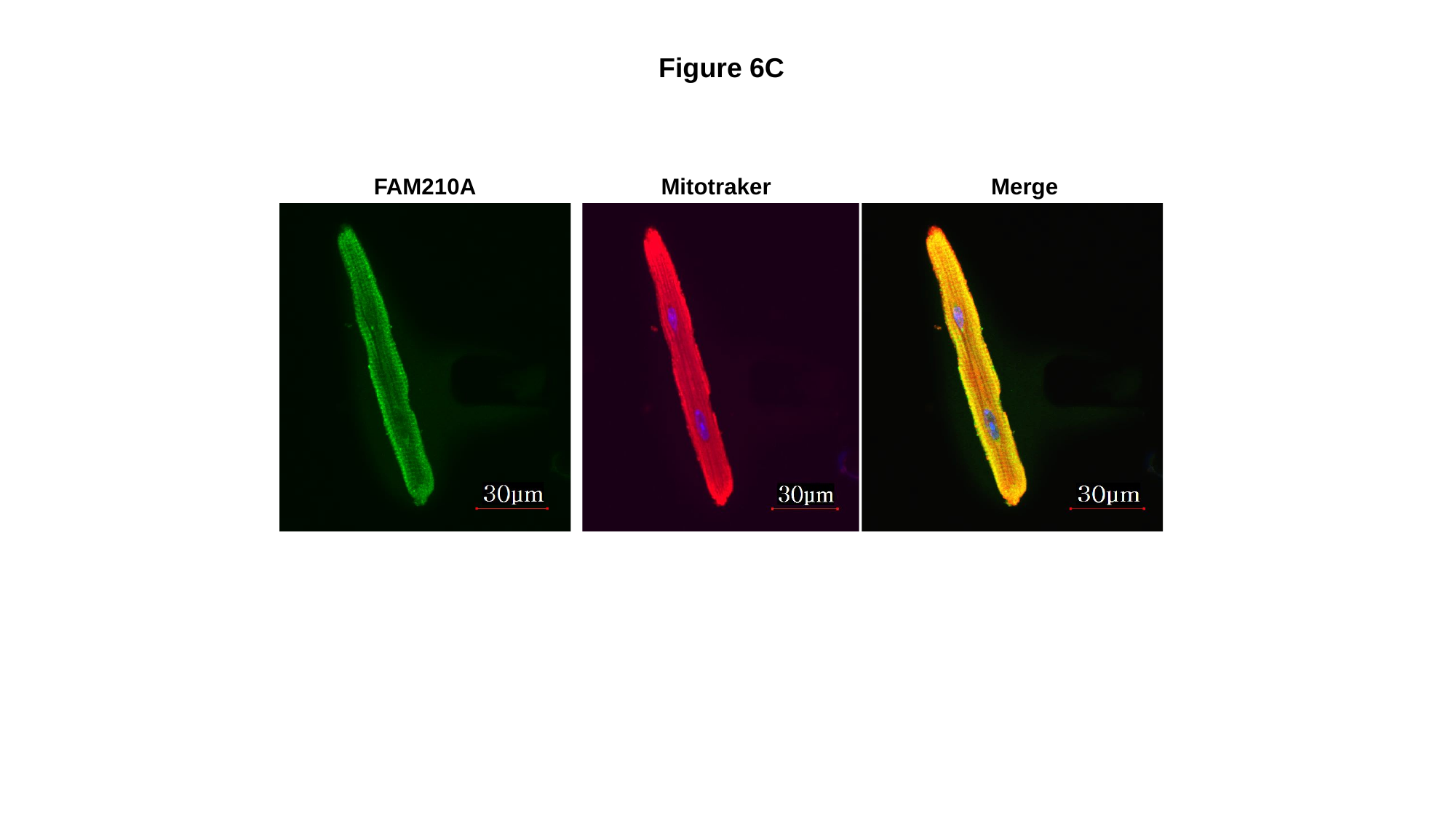

Figure 6C
FAM210A
Mitotraker
Merge

## Slide 2
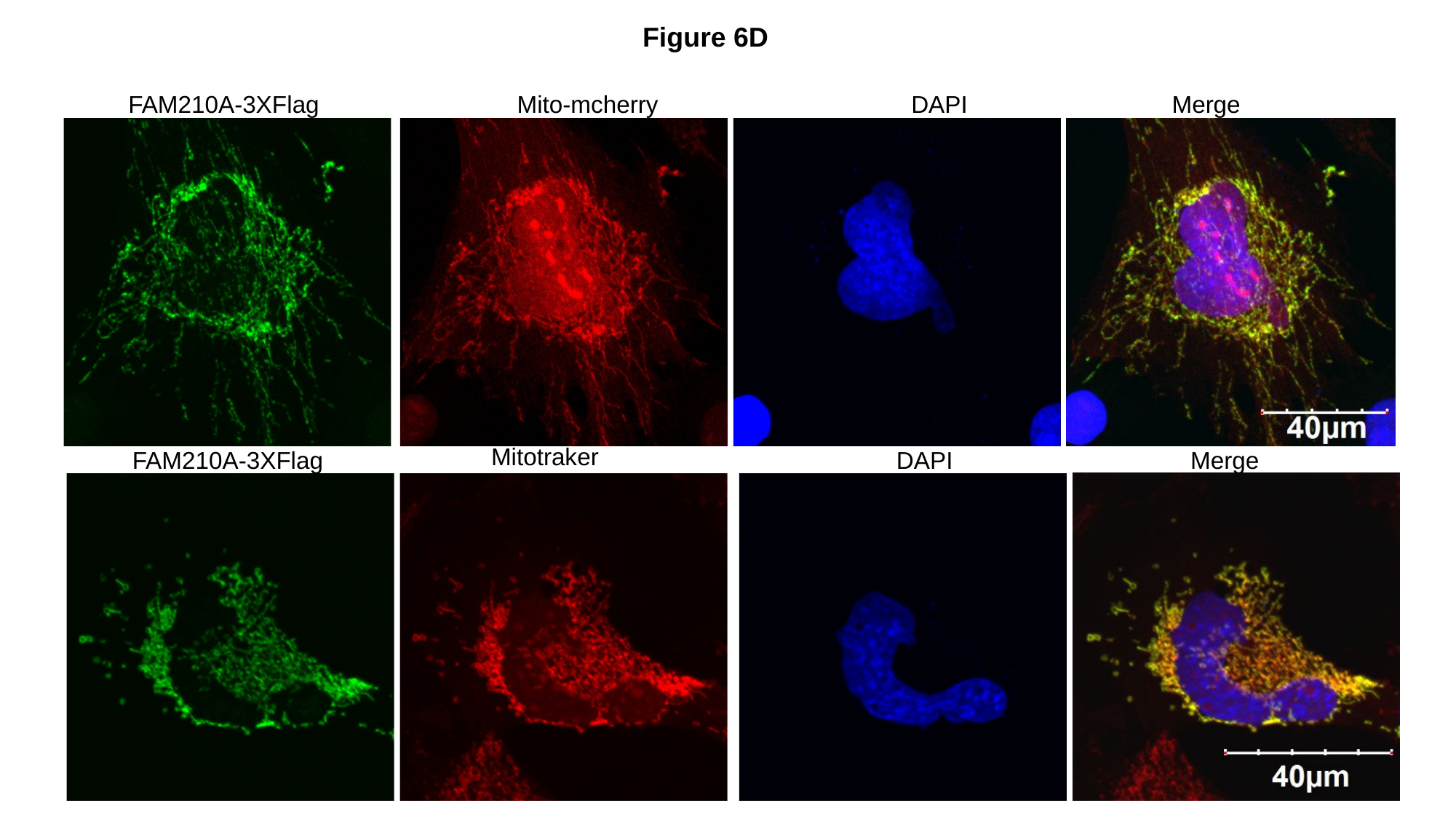

Figure 6D
FAM210A-3XFlag
Mito-mcherry
DAPI
Merge
Mitotraker
FAM210A-3XFlag
DAPI
Merge

## Slide 3
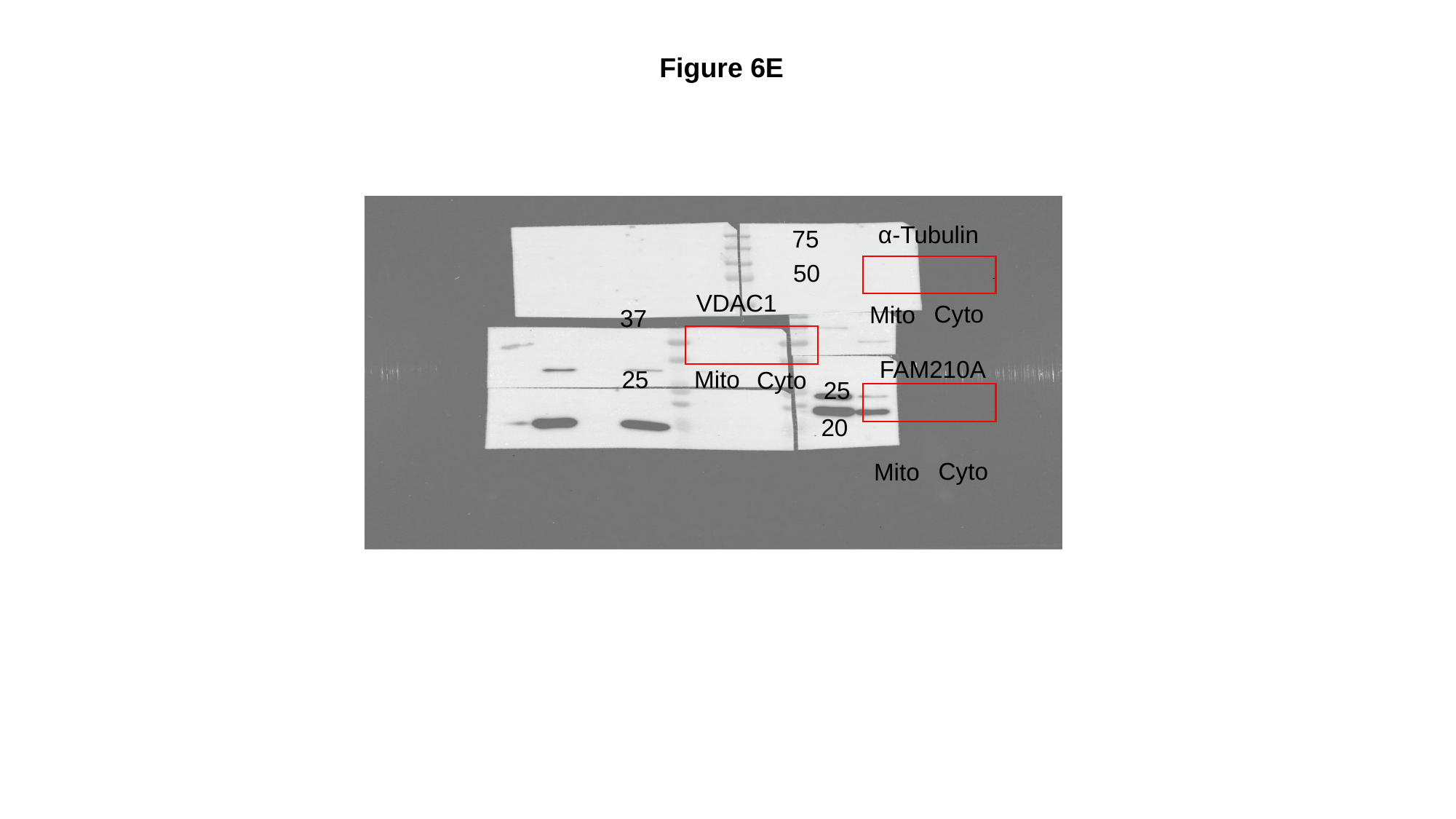

Figure 6E
α-Tubulin
75
50
VDAC1
Cyto
Mito
37
FAM210A
25
Mito
Cyto
25
20
Cyto
Mito

## Slide 4
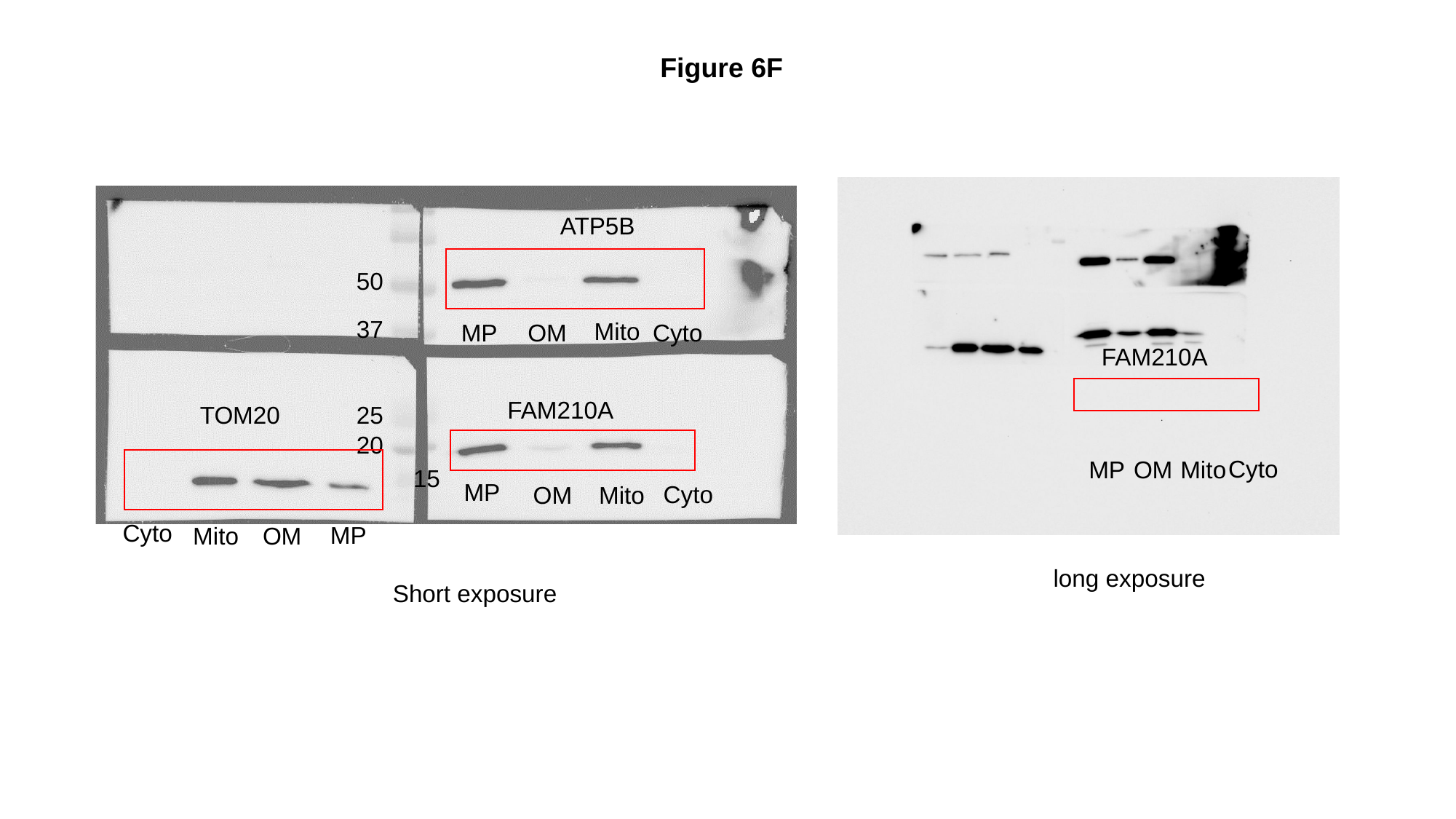

Figure 6F
ATP5B
50
37
Mito
MP
OM
Cyto
FAM210A
FAM210A
TOM20
25
20
Cyto
MP
OM
Mito
15
MP
Cyto
OM
Mito
Cyto
MP
Mito
OM
long exposure
Short exposure
