# Supplementary material for: MicroRNA‐574 regulates FAM210A expression and influences pathological cardiac remodeling
Source: EMBO Mol Med. 2020 Dec 28;13(2):e12710. doi: 10.15252/emmm.202012710 (PMC7863409; doi:10.15252/emmm.202012710)
Supplement: Supplementary file 10 — Source Data for Figure 7 [file EMMM-13-e12710-s008.zip › Figure 7.pptx]

## Slide 1
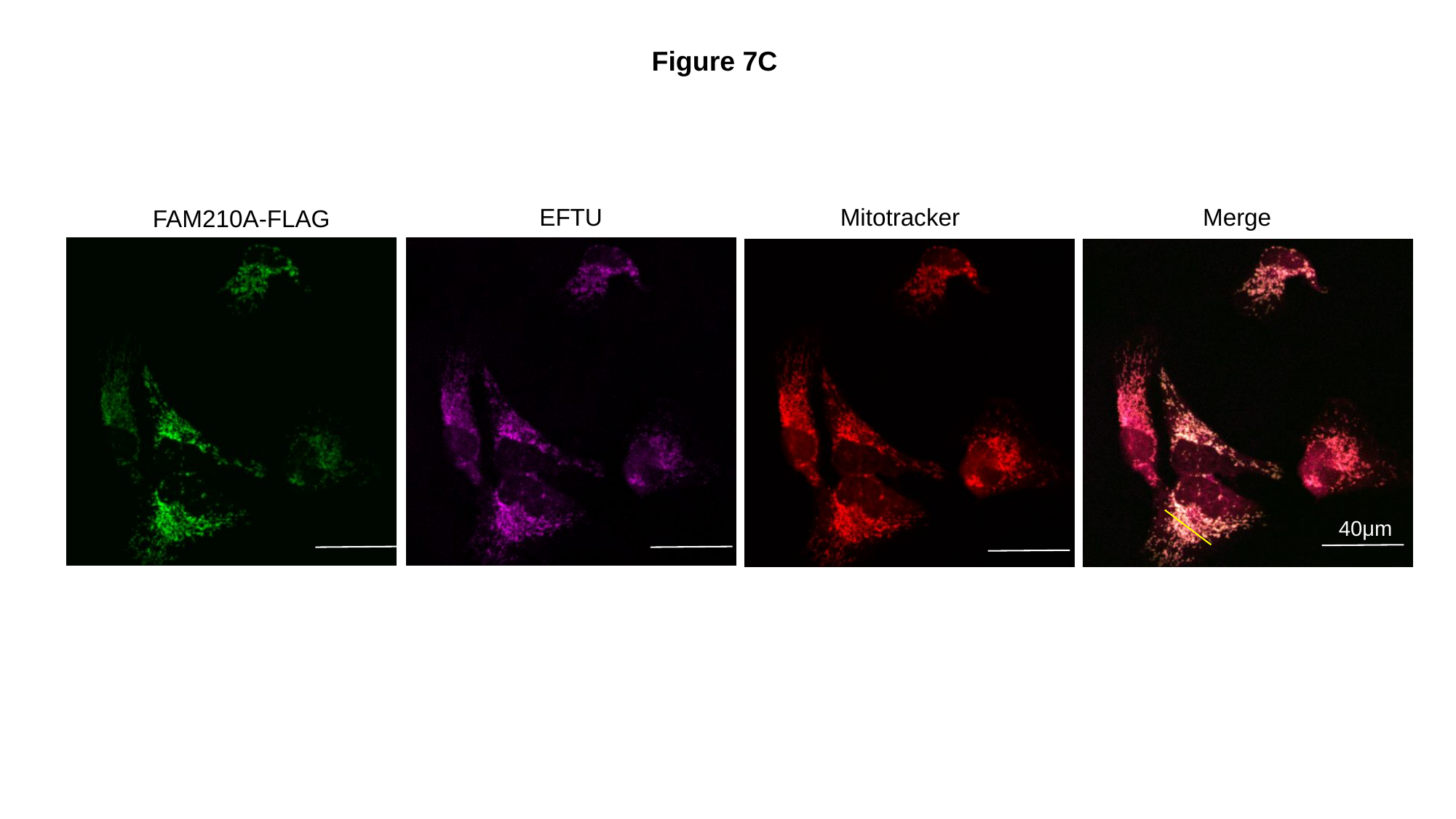

Figure 7C
Merge
EFTU
Mitotracker
FAM210A-FLAG
40μm

## Slide 2
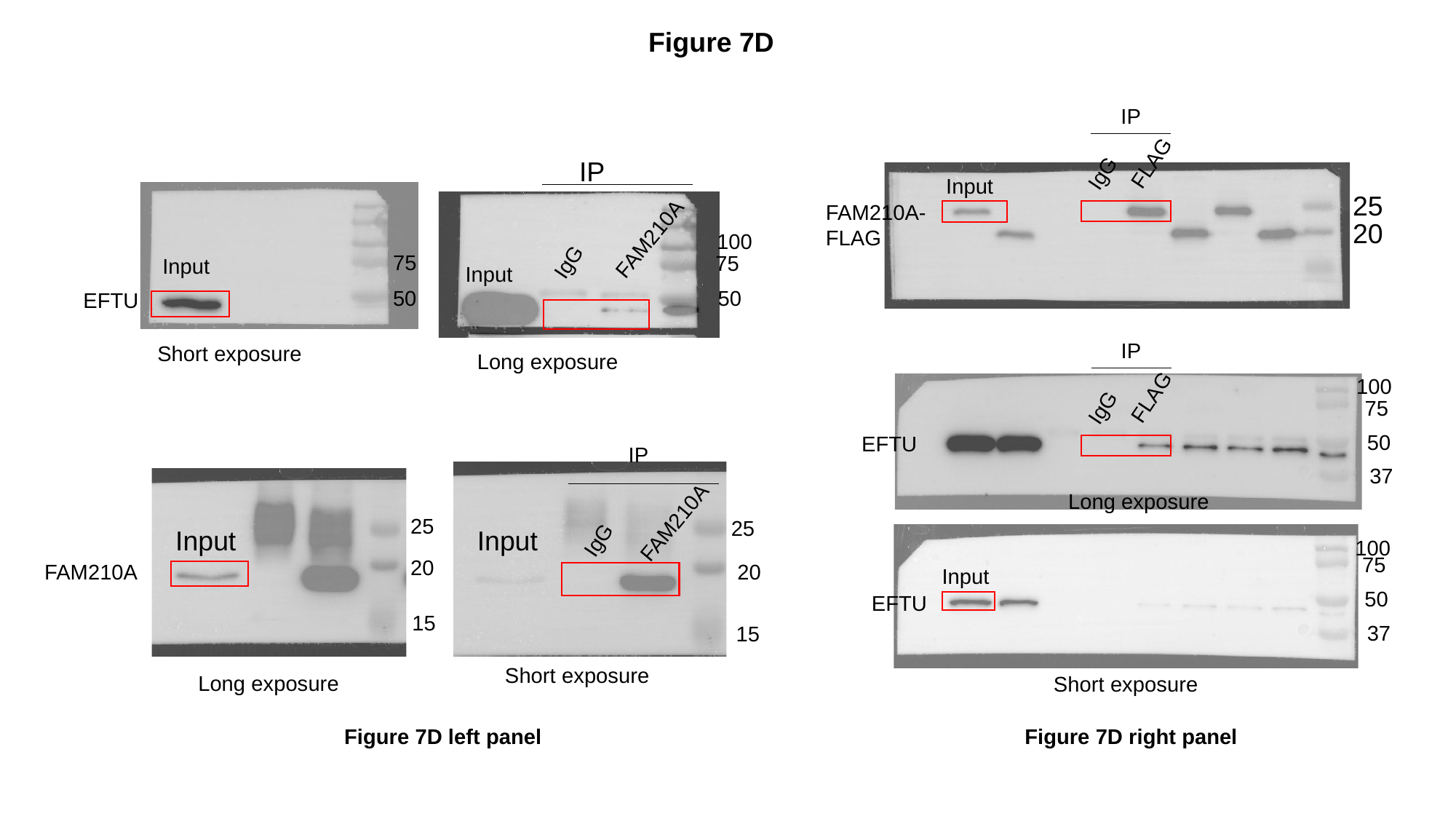

Figure 7D
IP
FLAG
IP
IgG
Input
25
FAM210A-FLAG
20
FAM210A
100
IgG
75
75
Input
Input
50
50
EFTU
IP
Short exposure
Long exposure
100
FLAG
IgG
75
50
EFTU
IP
37
Long exposure
FAM210A
25
25
Input
Input
IgG
100
75
20
FAM210A
20
Input
50
EFTU
15
37
15
Short exposure
Long exposure
Short exposure
Figure 7D left panel
Figure 7D right panel

## Slide 3
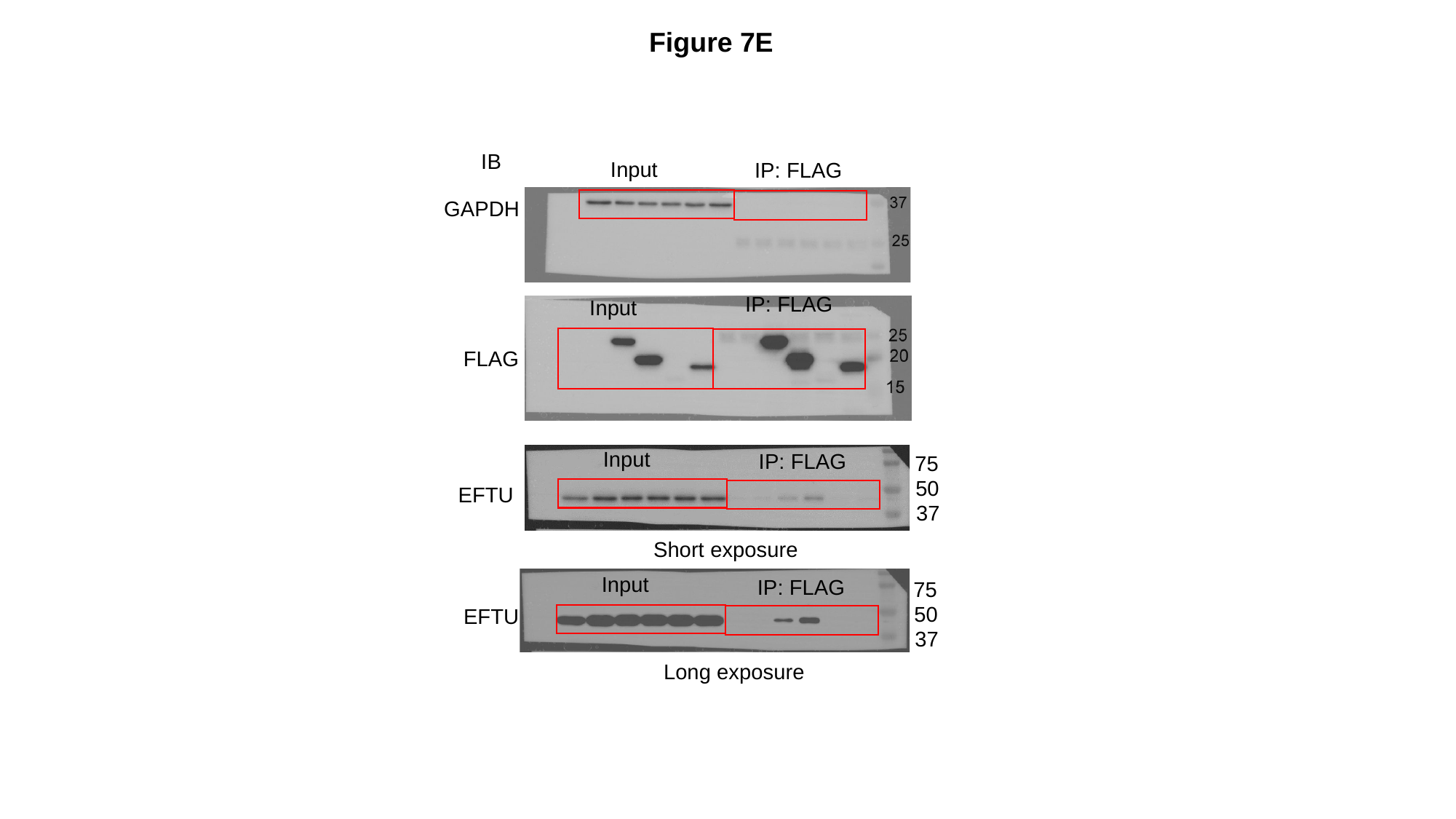

Figure 7E
IB
Input
IP: FLAG
GAPDH
IP: FLAG
Input
FLAG
Input
IP: FLAG
75
50
EFTU
37
Short exposure
Input
IP: FLAG
75
50
EFTU
37
Long exposure

## Slide 4
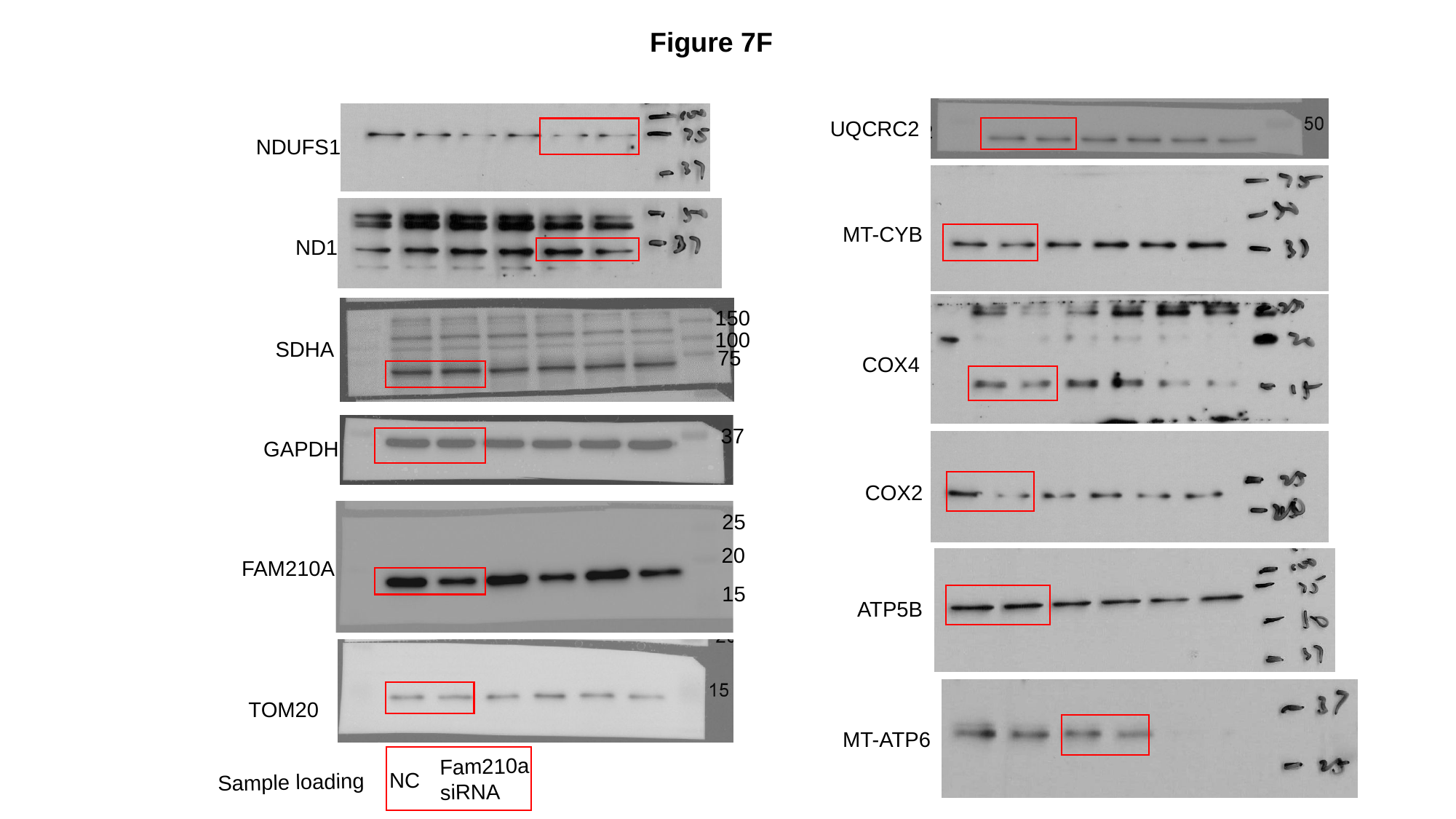

Figure 7F
UQCRC2
NDUFS1
MT-CYB
ND1
150
100
SDHA
75
COX4
37
GAPDH
COX2
25
20
FAM210A
15
ATP5B
TOM20
MT-ATP6
Fam210a
siRNA
NC
Sample loading

## Slide 5
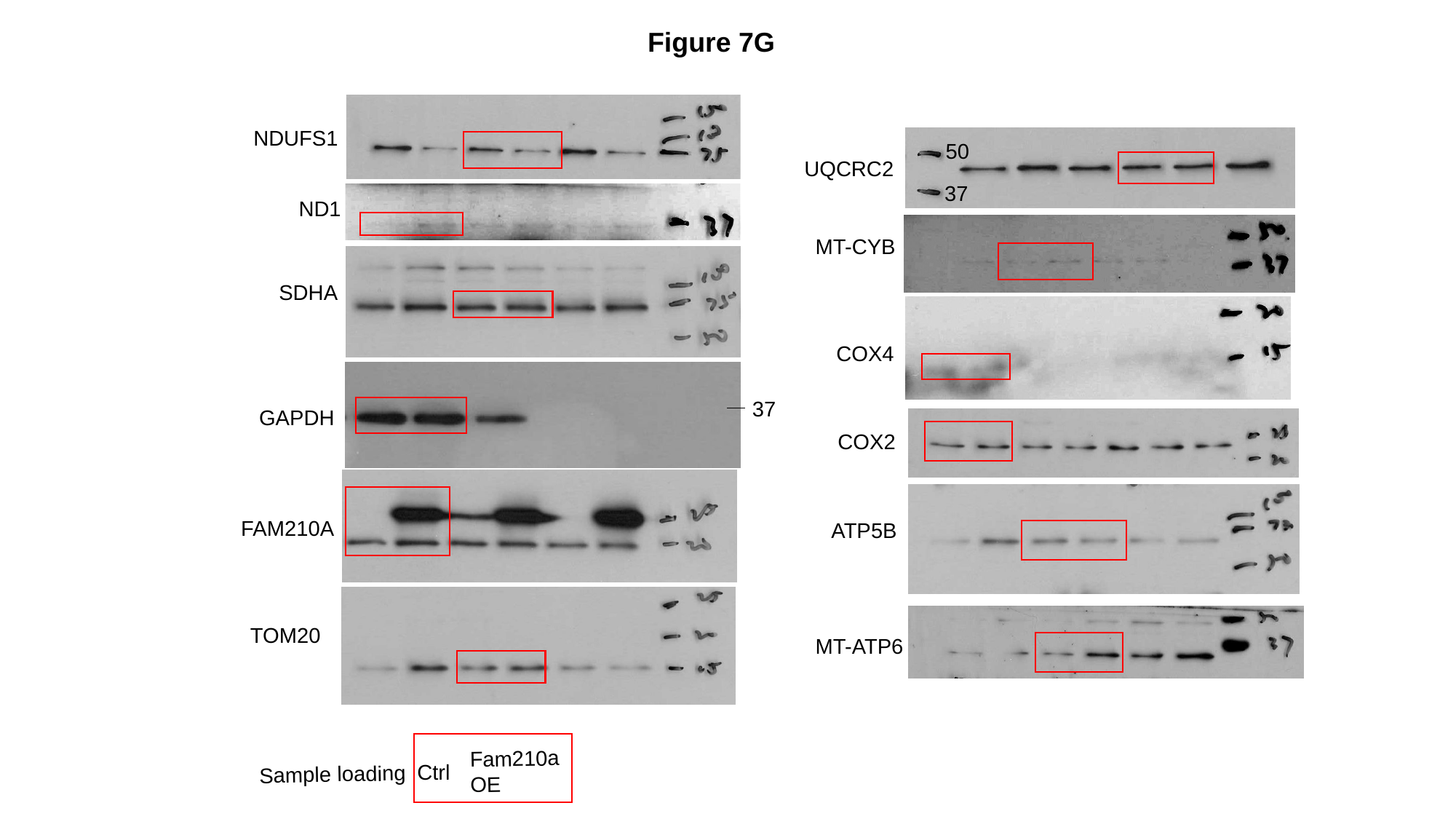

Figure 7G
NDUFS1
50
UQCRC2
37
ND1
MT-CYB
SDHA
COX4
37
GAPDH
COX2
FAM210A
ATP5B
TOM20
MT-ATP6
Fam210a
OE
Ctrl
Sample loading
