# Supplementary material for: MicroRNA‐574 regulates FAM210A expression and influences pathological cardiac remodeling
Source: EMBO Mol Med. 2020 Dec 28;13(2):e12710. doi: 10.15252/emmm.202012710 (PMC7863409; doi:10.15252/emmm.202012710)
Supplement: Supplementary file 11 — Source Data for Figure 8 [file EMMM-13-e12710-s009.zip › Figure 8.pptx]

## Slide 1
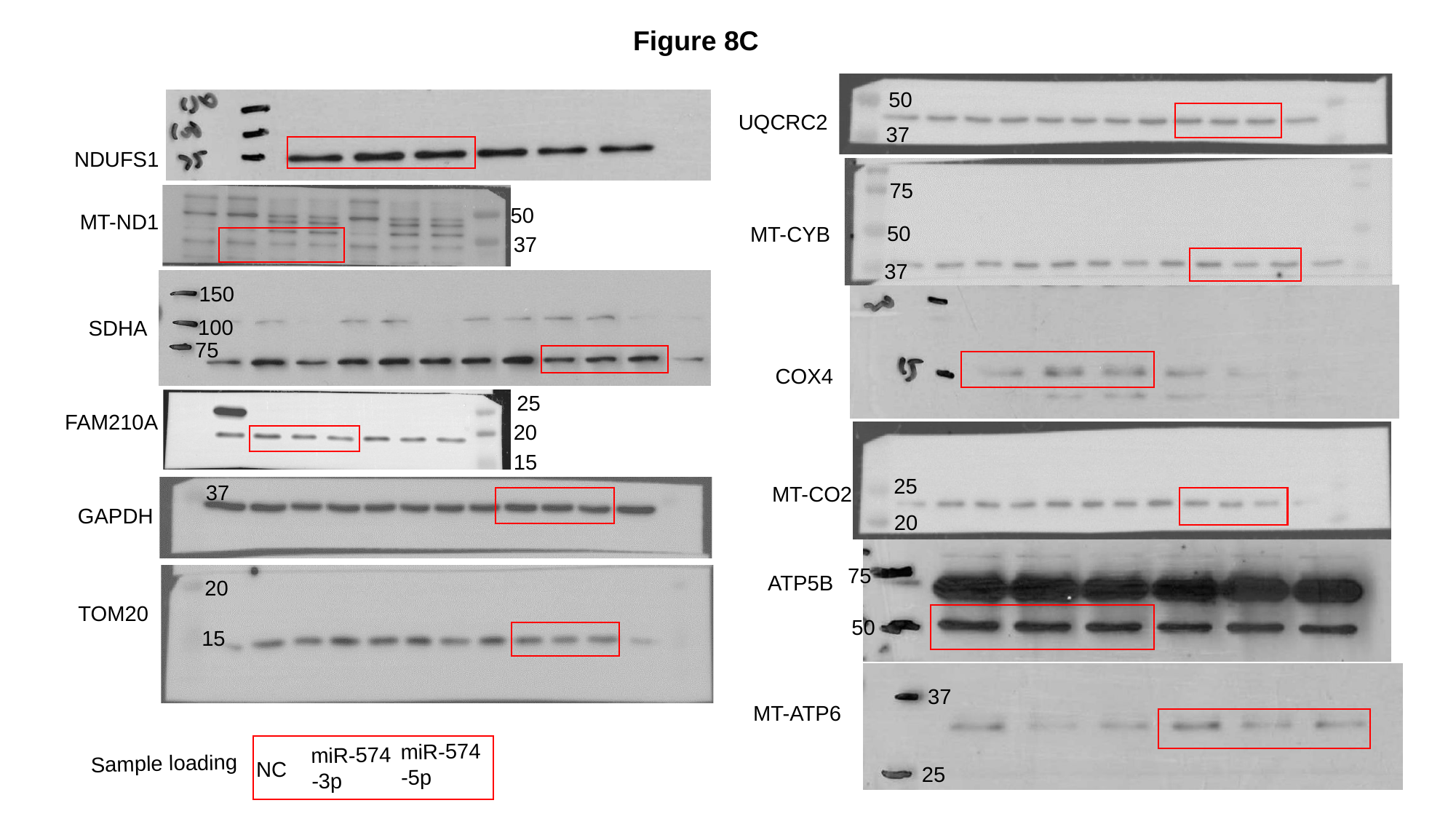

Figure 8C
50
UQCRC2
37
NDUFS1
75
50
MT-ND1
50
MT-CYB
37
37
150
100
SDHA
75
COX4
25
FAM210A
20
15
25
37
MT-CO2
GAPDH
20
75
ATP5B
20
TOM20
50
15
37
MT-ATP6
miR-574
-5p
miR-574
-3p
NC
Sample loading
25

## Slide 2
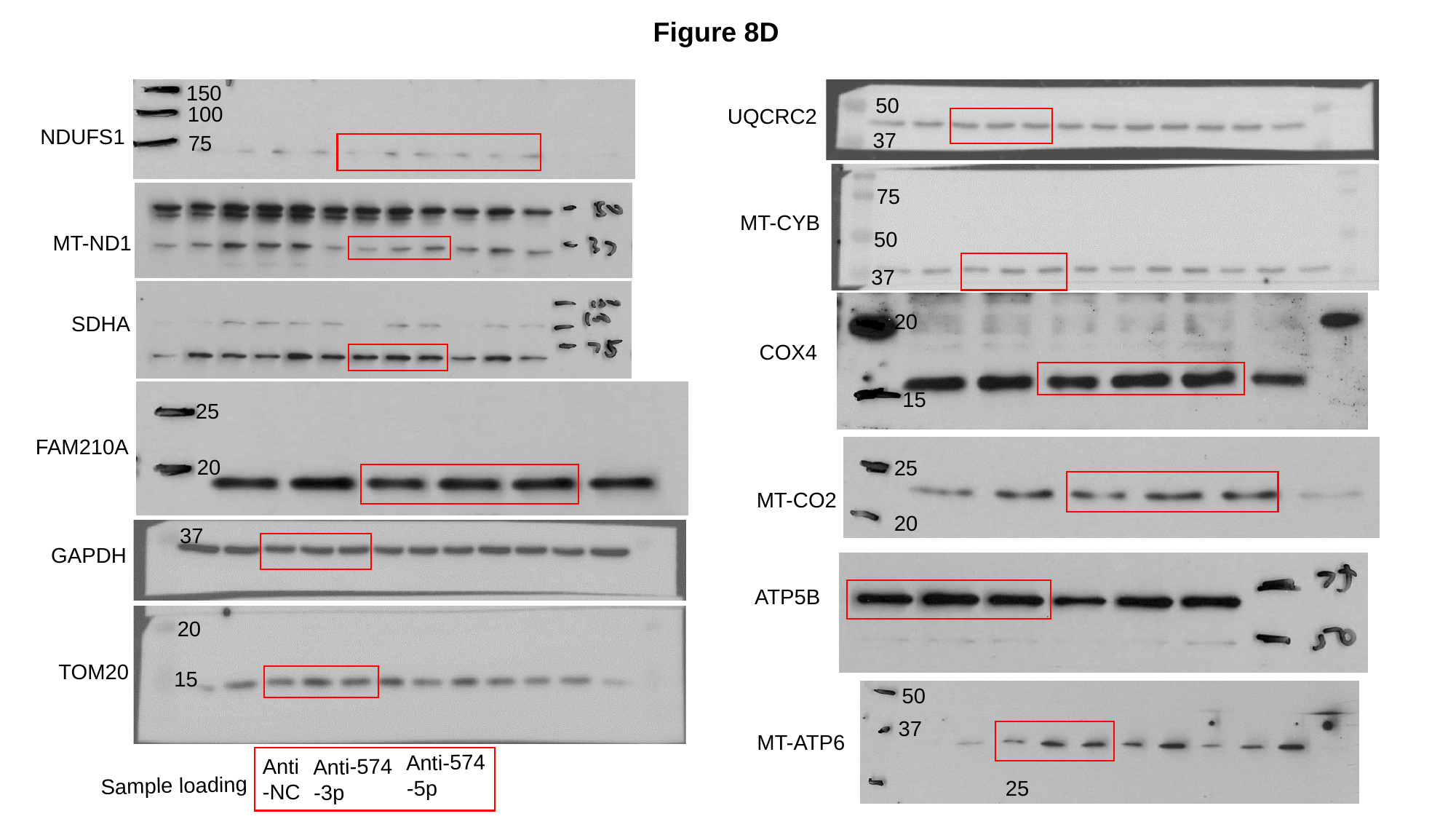

Figure 8D
150
50
100
UQCRC2
NDUFS1
37
75
75
MT-CYB
50
MT-ND1
37
20
SDHA
COX4
15
25
FAM210A
20
25
MT-CO2
20
37
GAPDH
ATP5B
20
TOM20
15
50
37
MT-ATP6
Anti-574
-5p
Anti-574
-3p
Anti-NC
Sample loading
25

## Slide 3
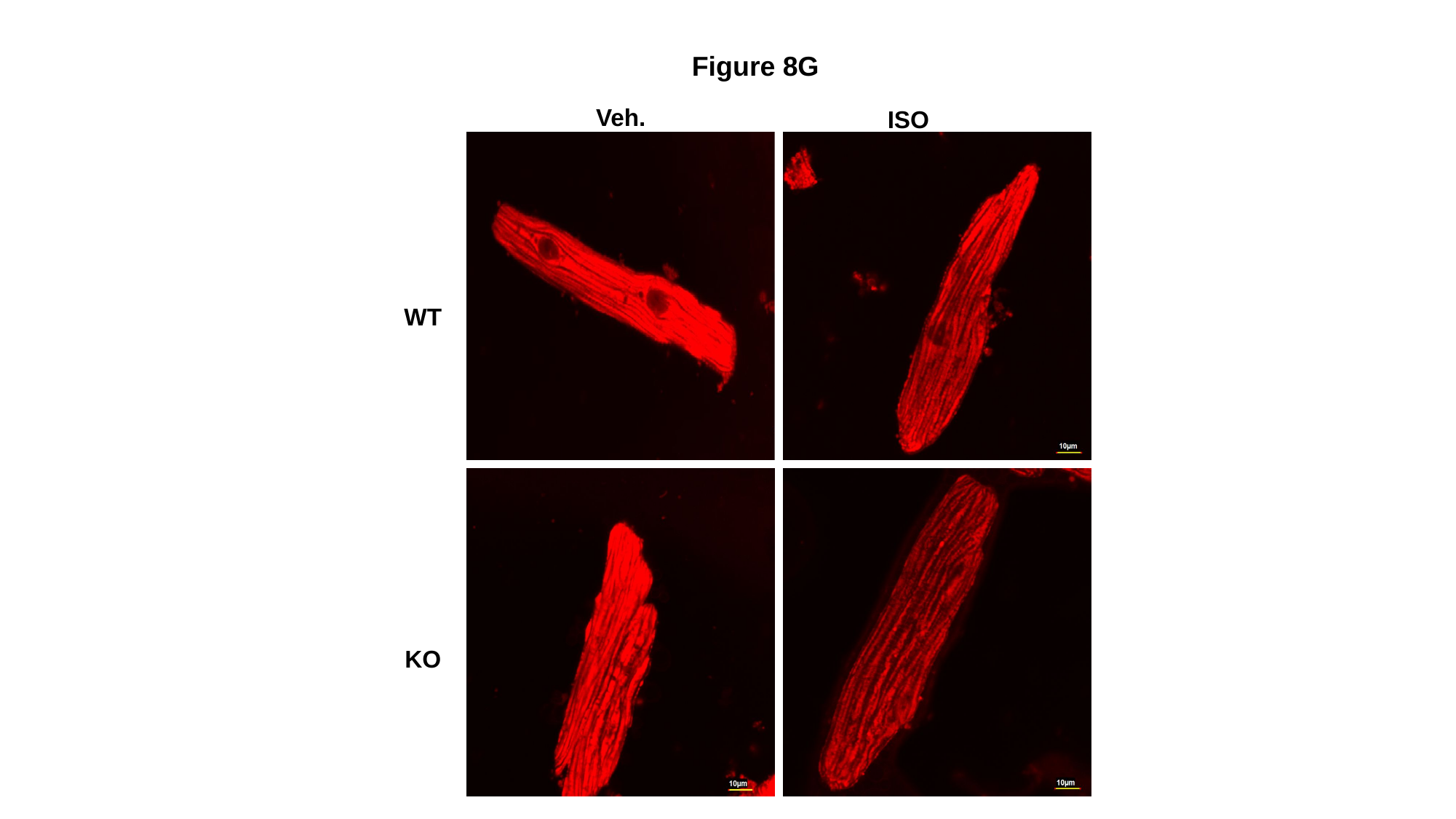

Figure 8G
Veh.
ISO
WT
KO

## Slide 4
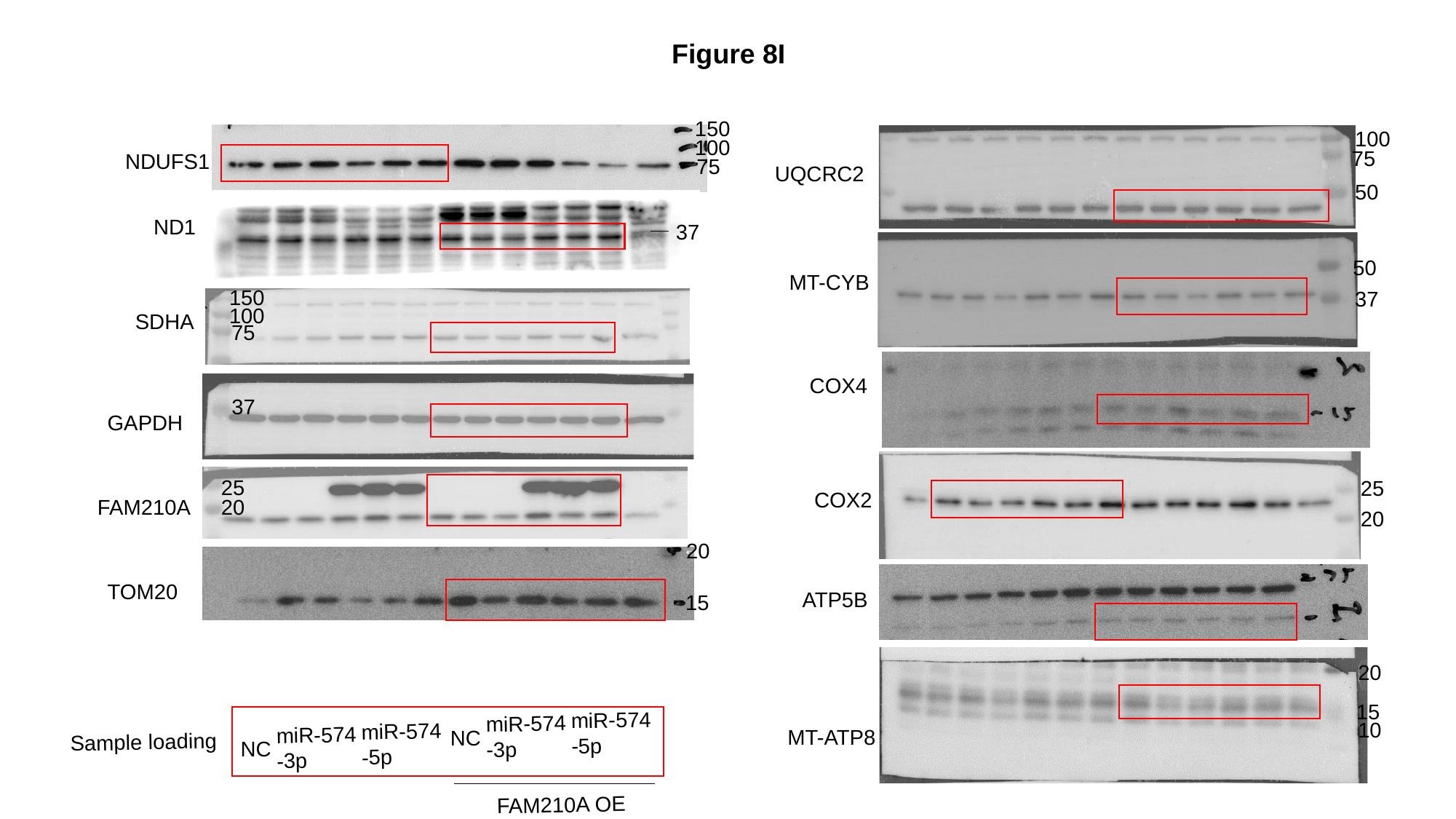

Figure 8I
150
100
100
75
NDUFS1
75
UQCRC2
50
ND1
37
50
MT-CYB
150
37
100
SDHA
75
COX4
37
GAPDH
25
25
COX2
FAM210A
20
20
20
TOM20
ATP5B
15
20
15
miR-574
-5p
miR-574
-3p
miR-574
-5p
miR-574
-3p
NC
NC
FAM210A OE
10
MT-ATP8
Sample loading
